# One-pot four-component sequential synthesis of *S*-alkyl dithiocarbamates using lipase as a biocatalyst

**DOI:** 10.3762/bjoc.22.83

**Published:** 2026-07-10

**Authors:** Mansour Shahedi, Pargol Tahmasebi pour, Zohreh Habibi

**Affiliations:** 1 Department of Organic Chemistry, Shahid Beheshti University, 1983969411 Tehran, Iran

**Keywords:** biocatalysis, dithiocarbamate, four-component, lipase, one-pot

## Abstract

Dithiocarbamates are widely recognized for their versatile applications in both agriculture as effective pesticides and medicine, where they serve as antifungal and anticancer agents. As a result, their synthesis has garnered significant attention in recent years. In this study, we present an efficient one-pot four-component approach for the synthesis of these scaffolds, utilizing aldehydes, ethyl acetoacetate, carbon disulfide (CS_2_), and amines. Initially, α,β-unsaturated carbonyl compounds, serving as Michael acceptors, were generated from aldehydes and ethyl acetoacetate through a decarboxylative Knoevenagel reaction under mild conditions, using lipase as a biocatalyst. These intermediates then sequentially undergo a nucleophilic addition reaction with dithiocarbamate anions, which are generated in situ by reacting CS_2_ with amines. This sequence successfully yields 15 derivatives of *S*-alkylated dithiocarbamates with high to excellent yields ranging from 69% to 96%.

## Introduction

**Figure 1:**
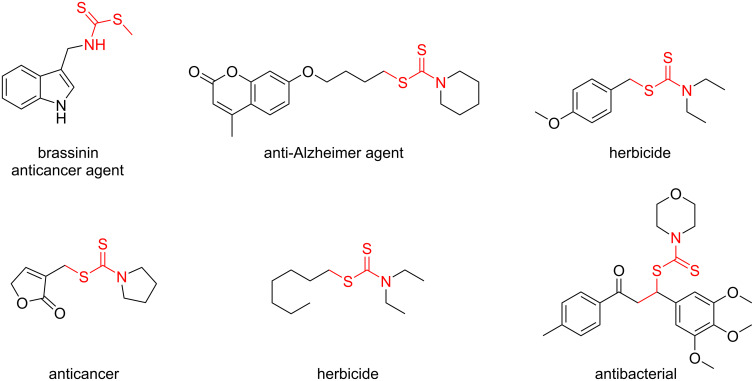
Biologically active *S*-alkyl dithiocarbamates*.*

Dithiocarbamates, a versatile class of organosulfur compounds, have attracted considerable interest due to their wide range of chemical and biological properties [1–2]. These compounds have been extensively studied for their applications in various fields, including pharmaceuticals [3–4], agrochemicals [5], and organic synthesis [6]. Among the different types of dithiocarbamates, *S*-alkyl dithiocarbamates (also known as organic dithiocarbamates) are particularly prominent, owing to their remarkable utility, especially in the pharmaceutical and agrochemical industries [7–8]. *S*-Alkyl dithiocarbamates demonstrate a broad spectrum of bioactivities, including potent anticancer [9], anti-Alzheimer [10–11], antibacterial [12], and antifungal [13–14] properties. In the realm of synthetic organic chemistry, these compounds serve as valuable intermediates, protecting groups, and polymerization agents, further enhancing their importance in chemical synthesis [15–16]. Figure 1 shows a selection of pharmaceutical agents and agrochemicals that incorporate *S*-alkyl dithiocarbamate scaffolds. Given their significant bioactivity and extensive applications, ongoing research into the development of efficient and diverse synthetic methodologies for *S*-alkyl dithiocarbamates remains crucial.

**Scheme 1:**
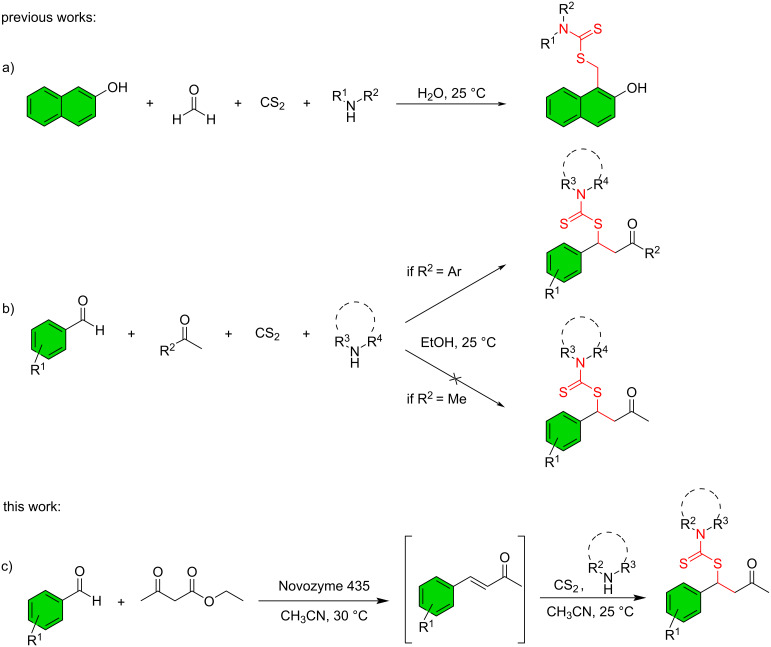
Previous works and this work for the sequential one-pot four-component synthesis of *S*-alkyl dithiocarbamates.

The synthesis of *S*-alkyl dithiocarbamates is primarily achieved through multicomponent reactions [17–18] that involve primary or secondary amines, carbon disulfide (CS_2_), and electrophilic alkyl precursors, such as alkyl halides [16], sulfonium salts [19], diazo compounds [20], methylarenes [21], or α,β-unsaturated compounds [8,22]. While three-component reactions are predominant in the synthesis of *S*-alkyl dithiocarbamates [23–24], the exploration of four-component, one-pot reactions remains limited. Notable advances in this area include a 2020 study by Halimehjani et al. who employed a four-component strategy involving β-naphthol, formaldehyde, amines, and carbon disulfide in water as the solvent to synthesize these compounds (Scheme 1a) [25]. Also, in 2013, Azizi et al. demonstrated a four-component reaction with benzaldehyde derivatives, aryl methyl ketones, amines, and carbon disulfide to form *S*-alkyl dithiocarbamates. However, when acetone was used as the fourth component in this reaction, no dithiocarbamate product was formed, and instead, benzylidene acetone was identified (Scheme 1b) [26].

Although several methodologies for the synthesis of *S*-alkyl dithiocarbamates have been reported, the integration of biocatalysis with sequential multicomponent synthesis remains unexplored. In the present work, a lipase-catalyzed Knoevenagel condensation/decarboxylation is used to generate the Michael acceptor in situ, which subsequently reacts with a dithiocarbamate nucleophile formed in situ from an amine and carbon disulfide, without isolation of intermediates. This sequential one-pot four-component strategy combines the advantages of biocatalysis, multicomponent synthesis, and operational simplicity, providing efficient access to structurally diverse *S*-alkyl dithiocarbamates under mild reaction conditions (Scheme 1c).

Lipases (triacylglycerol hydrolases, EC 3.1.1.3) are among the most widely utilized enzymes in both biotechnology and chemistry [27–29]. These enzymes primarily function to hydrolytically break or form C–O bonds as part of their physiological role [30]. Beyond this, lipases exhibit remarkable catalytic promiscuity, enabling them to catalyze the formation of carbon–carbon and carbon–heteroatom bonds [31–32]. This behavior has been exploited in a variety of reactions, including Michael [33] and Mannich [34] reactions. In 2009, Feng et al. demonstrated the use of this promiscuity in the Knoevenagel condensation/decarboxylation reaction to synthesize α,β-unsaturated compounds with ketone groups [35]. These compounds are valuable as Michael acceptors in the preparation of diverse organic molecules [36]. Building on this research, the present work explores the application of lipase catalytic promiscuity in the synthesis of *S*-alkyl dithiocarbamates, expanding the utility of lipases in the creation of valuable organic compounds.

## Results and Discussion

**Scheme 2:**
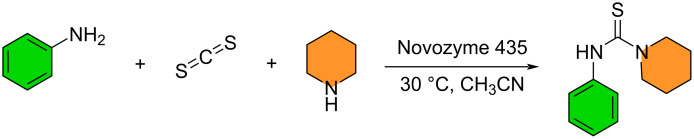
Formation of the thiourea by-product.

Initially, the first step of the selected model reaction, involving the condensation of 0.1 mmol benzaldehyde (**1a**) with 0.15 mmol of ethyl acetoacetate (**2a**), followed by decarboxylation was conducted based on previous studies [35–36]. Acetonitrile was selected as the solvent, and aniline was used as an additive at 30 °C. After confirming the formation of the Michael acceptor **3a**, the reaction was further investigated with varying amounts of amine **5a** and carbon disulfide (CS_2_, **4**) at different temperatures. Initially, 0.1 mmol of amine **5a** and 0.15 mmol of **4** were introduced to the reaction mixture. The progress of the reaction was monitored via thin-layer chromatography and after stirring for 12 h at 30 °C, no further product formation was observed. Under these conditions, the yield of product **6b** was 36% (Table 1, entry 1), with a side product also forming at a yield of 31%. This side product was identified as a thiourea derivative (Scheme 2) resulting from the reaction between aniline, carbon disulfide (**4**), and amine **5a**. To optimize the reaction and minimize side-product formation, after confirming the formation of **3a** at 30 °C, the amine **5a** and carbon disulfide (**4**) were added to the reaction mixture, and the reaction was continued at 25 °C. Monitoring the reaction via TLC revealed a significant reduction in the amount of side product, with the desired product **6b** obtained at a 51% yield after 12 h (Table 1, entry 2). Based on this result, 25 °C was determined to be the optimal temperature for the second step. In the next phase, the amounts of amine and carbon disulfide were varied to identify the optimal quantities. Increasing the amount of amine **5a** to 0.2 mmol and carbon disulfide (**4**) to 0.4 mmol significantly improved the yield to 86% (Table 1, entry 3). Subsequently, we tested a reduced enzyme quantity from 30 mg to 15 mg which resulted in a decreased yield of 54% (Table 1, entry 4). Thus, 30 mg of enzyme was selected as the optimal catalyst amount. Extending the reaction time to 24 hours did not lead to a notable change in yield (Table 1, entry 5). To explore the impact of different solvents, after the formation of the Michael acceptor **3a** in acetonitrile, the solvent was removed under reduced pressure, and various solvents with the amine and CS_2_ were added to the reaction mixture. It was found that the reactions in ethanol (Table 1, entry 6), THF (Table 1, entry 7), and H_2_O (Table 1, entry 8) resulted in yields of 71%, 65%, and 73%, respectively. In contrast, using a non-polar solvent such as *n*-hexane resulted in a significantly lower yield of 32% (Table 1 entry 9).

**Table 1:** Optimization of model reaction.^a^

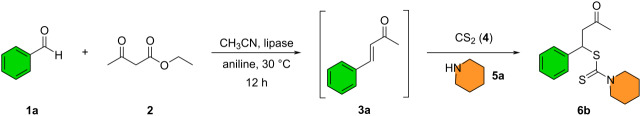							

Entry	Amine (mmol)	CS_2_ (mmol)	Enzyme (mg)	Temperature (°C)	Time (h)	Solvent	Yield (%)

1	0.1	0.15	30	30	12	CH_3_CN	36
2	0.1	0.15	30	25	12	CH_3_CN	51
**3**	**0.2**	**0.4**	**30**	**25**	**12**	**CH** **_3_** **CN**	**86**
4	0.2	0.4	15	25	12	CH_3_CN	54
5	0.2	0.4	30	25	24	CH_3_CN	84
6	0.2	0.4	30	25	12	EtOH^b^	71
7	0.2	0.4	30	25	12	THF^b^	65
8	0.2	0.4	30	25	12	H_2_O^b^	73
9	0.2	0.4	30	25	12	*n*-hexane^b^	32

^a^Model reaction conditions: the mixture of the aldehyde **1a** (0.1 mmol), ethyl acetoacetate (**2**, 0.15 mmol), aniline (0.05 mmol) and lipase (30 or 15 mg) in CH_3_CN was stirred at 30 °C for 12 h, then CS_2_ (**4**) and piperidine (**5a**) were added and stirring was continued at the indicated temperature and time. ^b^After the first step, CH_3_CN was removed under reduced pressure and compounds **4** and **5a** in the indicated solvent were added.

**Scheme 3:**
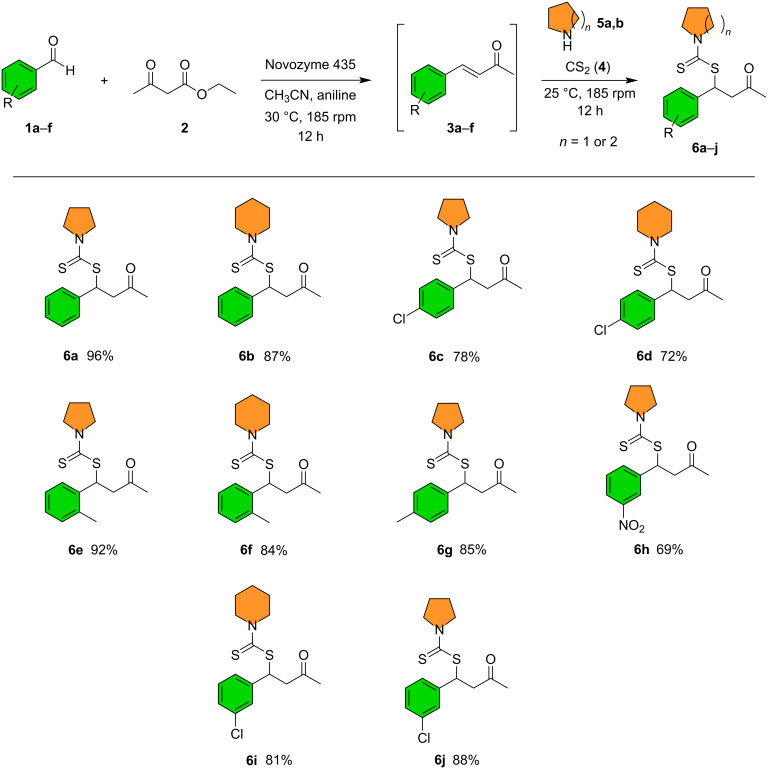
Scope of reaction with aldehydes and cyclic amines. Reaction conditions: **1a–f** (0.1 mmol), **2** (0.15 mmol), Novozyme 435 (30 mg), aniline (0.05 mmol), CH_3_CN (1 mL), 30 °C, 12 h; then, CS_2_ (**4,** 0.4 mmol), cyclic amine **5a,b** (0.2 mmol), 25 °C, 12 h.

**Scheme 4:**
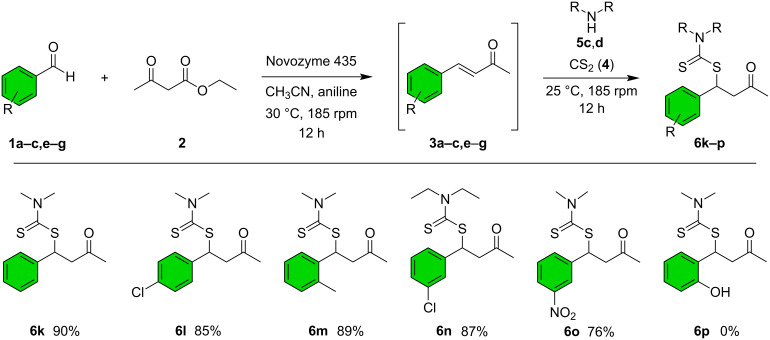
Scope of the reaction with aldehydes and acyclic amines. Reaction conditions: **1a–c**,**e–g** (0.1 mmol), **2** (0.15 mmol), Novozyme 435 (30 mg), aniline (0.05 mmol), CH_3_CN (1 mL), 30 °C, 12 h; then, CS_2_ (**4**, 0.4 mmol), amine **5c**,**d** (0.2 mmol), 25 °C, 12 h.

After having optimized the reaction conditions, the scope of the reaction was investigated using various aldehydes and amines, as presented in Scheme 3 and Scheme 4. As shown in Scheme 3, the reaction with cyclic amines such as pyrrolidine resulted in a higher product yield (**6a**, 96%) compared to piperidine (**6b**, 87%). Additionally, both benzaldehyde (**6a**, 96%) and 2-methylbenzaldehyde (**6e**, 92%) afforded the desired products in excellent yields, indicating that the reaction tolerates moderate steric effects. It is also noteworthy that aldehydes bearing electron-withdrawing substituents, such as 4-Cl (**6c**, 72%), 3-NO_2_ (**6h**, 69%), and 3-Cl (**6i**, 81%) led to decreased yields.

Additionally, acyclic amines such as dimethylamine and diethylamine were treated with various Michael acceptors to yield products **6k–o** (Scheme 4). Aldehydes bearing electron-withdrawing substituents, such as 4-chloro (**6l**, 85%) and 3-NO_2_ (**6o**, 76%) also afforded the desired products in good to excellent yields. These results indicate that the methodology is compatible with electronically diverse aldehydes. Notably, the reaction with salicylaldehyde resulted in no product formation (**6p**, 0%). It should be noted that, in principle, the formation of a stereogenic center in the product raises the possibility of enantioselectivity in the presence of a biocatalyst. However, in previously reported lipase-catalyzed promiscuous reactions, significant stereochemical induction has generally not been observed or discussed [36–38].

**Scheme 5:**
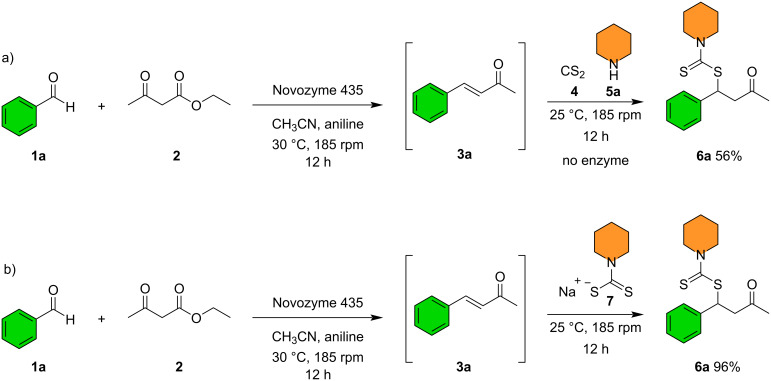
Control reactions.

To investigate the reaction mechanism and the catalytic role of the enzyme in enhancing the efficiency of the second step, control reactions were conducted (Scheme 5). For instance, after confirming the formation of the Michael acceptor **3a**, the enzyme was removed from the reaction mixture using filter paper, and then the amine and carbon disulfide were introduced. After 12 hours, the yield dropped to 56%, indicating a decrease in reaction efficiency in the absence of the enzyme. This result highlights the crucial catalytic role of lipase in the second step (Scheme 5a). Additionally, for the thia-Michael reaction step, the dithiocarbamate anion **7** was prepared separately and added to the reaction vessel. However, this did not affect the reaction efficiency when compared to the optimized method (Scheme 5b).

**Scheme 6:**
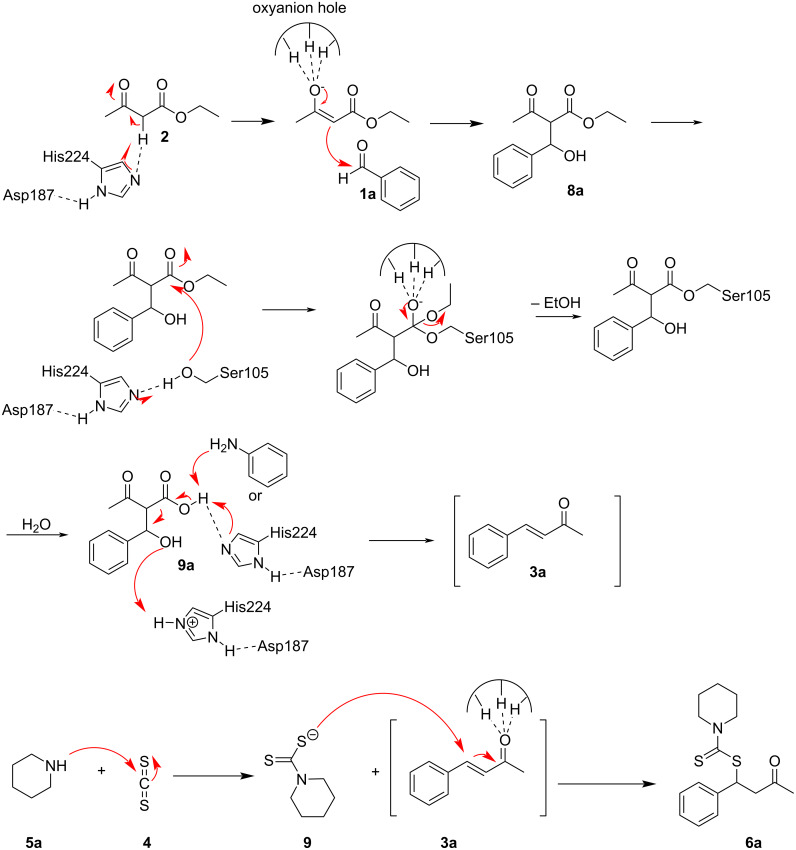
Proposed mechanism.

Based on the control reactions and previous studies a mechanism is proposed (Scheme 6). The reaction begins with a nucleophilic attack by the activated ketoester, facilitated by the triad amino acids of lipase, on the carbonyl group of the aldehyde, resulting in the formation of the intermediate **8a**. Next, the ester portion of the ketoester is hydrolyzed through the serine amino acid, leading to the formation of the intermediate **9a**. This intermediate then undergoes decarboxylation, which can be assisted by histidine or aniline, resulting in the formation of the Michael acceptor **3a**. Subsequently, the intermediate **7**, formed from the reaction of carbon disulfide **4** and amine **5a**, attacks the double bond of **3a**, leading to the formation of the product **6a**.

## Conclusion

In conclusion, an efficient sequential one-pot four-component methodology was developed for the synthesis of *S*-alkyl dithiocarbamates using Novozym 435 as a biocatalyst. The protocol enables the in situ generation of the Michael acceptor through a lipase-catalyzed Knoevenagel condensation/decarboxylation reaction, followed by addition of a dithiocarbamate nucleophile formed from an amine and carbon disulfide, without isolation of intermediates. Under mild reaction conditions, 15 derivatives were obtained in good to excellent yields. Beyond the preparation of the target compounds, this study highlights the potential of lipase catalytic promiscuity in sequential multicomponent transformations. The operational simplicity, mild conditions, and efficient access to structurally diverse *S*-alkyl dithiocarbamates demonstrate the synthetic utility of this approach. Considering the well-documented biological relevance of dithiocarbamate scaffolds, the synthesized compounds may provide a useful basis for future studies directed toward medicinal, antifungal, and agrochemical applications.

## Experimental

### General remarks

All reagents were commercially available and used without further purification. Solvents used for extraction and purification were distilled before use. Novozym 435 was a generous gift from Novozymes (Denmark). Progress of the reactions was monitored by thin-layer chromatography (TLC, performed on pre-coated Merck silica gel 60 F_254_ plates). All organic synthesis products were purified by preparative thin-layer chromatography (TLC), (CAMAG^®^ instrument, in-house prepared 20 × 20 cm silica plates) using a mixture of ethyl acetate and *n*-hexane as a mobile phase. ^1^H and ^13^C NMR spectra were recorded at 300 (75) MHz on a Bruker Avance spectrometer using CDCl_3_ as a solvent. The chemical shifts were referenced to the solvent signals at δH/C 7.26/77 ppm (chloroform-*d*) relative to TMS. Mass spectra were recorded with Agilent Technologies (HP) 5975c and G7081B mass spectrometers.

### Synthesis of *S*-alkyl ditihocarbamates **6a–o**

A mixture of aldehyde (0.1 mmol), ethyl acetoacetate (0.15 mmol), acetonitrile (1 mL), 0.05 mmol of aniline, and 30 mg of Novozyme 435 was added to the reaction vessel. The reaction mixture was stirred at 30 °C for 12 hours using a magnetic stirrer. The formation of the Michael acceptor was monitored via thin-layer chromatography (TLC). Afterward, carbon disulfide (0.4 mmol) and amine (0.2 mmol) were introduced into the reaction mixture, and stirring was continued at 25 °C for another 12 hours. The reaction progress was periodically checked by TLC. Following this, the mixture was filtered to remove the enzyme, the solvent was evaporated under reduced pressure, and the desired product was isolated from the reaction mixture using preparative thin-layer chromatography (TLC) plates (eluting with *n*-hexane/ethyl acetate).

## Supporting Information

File 1 Product characterization data and copies of NMR and MS spectra.

## Data Availability

All data that supports the findings of this study is available in the published article and/or the supporting information of this article.
