# Supplementary material for: One-pot four-component sequential synthesis of S-alkyl dithiocarbamates using lipase as a biocatalyst
Source: Beilstein J Org Chem. 2026 Jul 10;22:1048–56. doi: 10.3762/bjoc.22.83 (PMC13358900; doi:10.3762/bjoc.22.83)
Supplement: File 1 — Product characterization data and copies of NMR and MS spectra. [file Beilstein_J_Org_Chem-22-1048-s001.pdf]

BEILSTEIN JOURNAL OF ORGANIC CHEMISTRY

Supporting Information

for

One-pot four-component sequential synthesis of *S*-alkyl dithiocarbamates using lipase as a biocatalyst

Mansour Shahedi, Pargol Tahmasebi pour and Zohreh Habibi

Beilstein J. Org. Chem. **2026,** *22,* 1048–1056. doi:10.3762/bjoc.22.83

Product characterization data and copies of NMR and MS spectra

License and Terms: This is a supporting information file under the terms of the Creative Commons Attribution License (https://creativecommons.org/licenses/by/4.0). Please note that the reuse, redistribution and reproduction in particular requires that the author(s) and source are credited and that individual graphics may be subject to special legal provisions.
The license is subject to the *Beilstein Journal of Organic Chemistry* terms and conditions: (https://www.beilstein-journals.org/bjoc/terms)

Table of contents

Characterization data for products: S2

^{1}H NMR, ^{13}C NMR {^{1}H} and mass spectra S10

Characterization data for products:

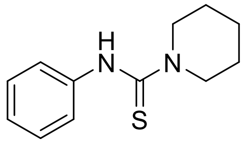

***N*-Phenylpiperidine-1-carbothioamide** [1], amorphous solid (white), ^{1}H NMR (300 MHz, chloroform-*d*) δ 7.38 – 7.27 (m, 2H), 7.13 (m, 3H), 3.78 (s, 4H), 1.67 (s, 6H). ^{13}C NMR (75 MHz, chloroform-*d*) δ 182.7, 140.3, 129.1, 124.8, 122.6, 50.9, 25.5, 24.1.

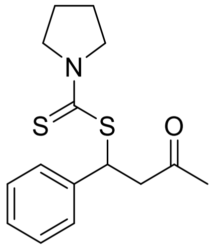

3-Oxo-1-phenylbutyl pyrrolidine-1-carbodithioate (**6a**) [2], yellow oil, isolated yield (96%, 28 mg), purification by preparative TLC (*n*-hexane/ethyl acetate 9:1), ^{1}H NMR (300 MHz, chloroform-*d*) δ 7.49 – 7.38 (m, 2H), 7.38 – 7.22 (m, 3H), 5.62 (dd, *J* = 9.9, 5.0 Hz, 1H), 3.92 (t, *J* = 6.8 Hz, 2H), 3.68 – 3.45 (m, 3H), 3.18 (dd, *J* = 16.4, 9.9 Hz, 1H), 2.15 (s, 3H), 2.03 (m, 4H). ^{13}C NMR (75 MHz, chloroform-*d*) δ 205.7, 191.0, 139.2, 128.7, 128.2, 127.7, 54.9, 50.5, 50.3, 49.9, 29.8, 26.0, 24.2. MS: (EI, 70 eV): m/z = 293 [M^+], Anal. Calcd for $C_{15}H_{19}NOS_2$: C 61.40; H 6.53; N 4.77; Found: C 61.15; H 6.13; N 4.70.

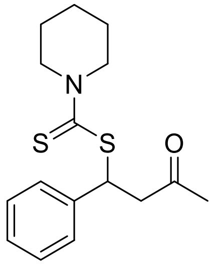

3-Oxo-1-phenylbutyl piperidine-1-carbodithioate (**6b**) [3], yellow oil, isolated yield (87% 26.72 mg), purification by preparative TLC (*n*-hexane/ethyl acetate 9:1), ^{1}H NMR (300 MHz, chloroform-*d*) δ 7.46 – 7.37 (m, 2H), 7.34 – 7.25 (m, 3H), 5.58 (dd, *J* = 10.1, 4.6 Hz, 1H), 4.27 (d, *J* = 33.0 Hz, 2H), 3.83 (s, 2H), 3.54 (dd, *J* = 16.3, 4.6 Hz, 1H), 3.18 (dd, *J* = 16.3, 10.2 Hz, 1H), 2.14 (d, *J* = 1.5 Hz, 3H), 1.69 (s, 6H). ^{13}C NMR (75 MHz, chloroform-*d*) δ 205.7, 190.9,

139.2, 128.7, 128.2, 127.7, 54.9, 50.5, 50.3, 49.9, 29.8, 26.0, 24.2. MS: (EI, 70 eV): m/z = 307 $[M^+]$, Anal. Calcd for $C_{16}H_{21}NOS_2$: C 62.50; H 6.83; N 4.56; Found: C 62.55; H 6.61; N 4.40.

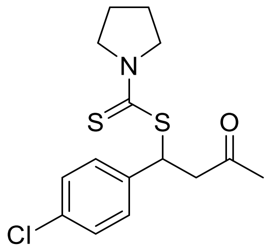

1-(4-Chlorophenyl)-3-oxobutyl pyrrolidine-1-carbodithioate (6c), brown oil, isolated yield (78%, 25.57 mg), purification by preparative TLC (*n*-hexane/ethyl acetate 9:1), ^{1}H NMR (300 MHz, chloroform-*d*) δ 7.37 (d, *J* = 8.4 Hz, 2H), 7.28 (d, *J* = 8.5 Hz, 2H), 5.59 (dd, *J* = 9.7, 5.0 Hz, 1H), 3.91 (t, *J* = 6.9 Hz, 2H), 3.57 (d, *J* = 7.7 Hz, 2H), 3.46 (dd, *J* = 16.4, 4.7 Hz, 1H), 3.14 (dd, *J* = 16.7, 9.7 Hz, 1H), 2.14 (s, 3H), 2.10 – 2.04 (m, 2H), 2.01 – 1.93 (m, 2H). ^{13}C NMR (75 MHz, chloroform-*d*) δ 205.4, 190.5, 138.2, 133.4, 129.7, 128.8, 55.0, 50.6, 49.4, 46.42, 29.9, 26.0, 24.2. MS: (EI, 70 eV): m/z = 328 [M+], Anal. Calcd for $C_{15}H_{18}ClNOS_2$: C 54.95; H 5.53; N 4.27; Found: C 54.56; H 5.19; N 4.01.

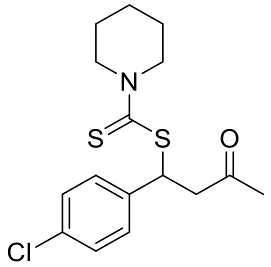

1-(4-Chlorophenyl)-3-oxobutyl piperidine-1-carbodithioate (6d), brown oil, isolated yield (72%, 24.61 mg), purification by preparative TLC (*n*-hexane/ethyl acetate 9:1), ^{1}H NMR (300 MHz, chloroform-*d*) δ 7.37 (d, *J* = 8.1 Hz, 3H), 7.29 (d, *J* = 7.8 Hz, 2H), 5.56 (m, 1H), 4.26 (m, 2H), 3.85 (m, 2H), 3.49 (dd, *J* = 16.7, 4.7 Hz, 1H), 3.14 (dd, *J* = 16.7, 4.5 Hz, 1H), 2.14 (s, 3H), 1.68 (s, 6H). ^{13}C NMR (75 MHz, chloroform-*d*) δ 207.1, 198.2, 141.9, 136.4, 129.8, 129.4, 129.2, 128.8, 127.4, 50.9, 31.0, 27.7, 22.5. MS: (EI, 70 eV): m/z = 341 $[M^+]$, Anal. Calcd for $C_{16}H_{20}ClNOS_2$: C 56.21; H 5.90; N 4.10; Found: C 56.13; H 5.83; N 4.20.

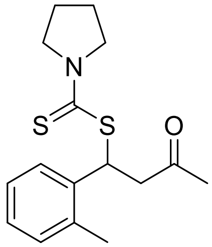

3-Oxo-1-(*o*-tolyl)butyl pyrrolidine-1-carbodithioate (**6e**), yellow oil, isolated yield (92%, 28.61 mg), purification by preparative TLC (*n*-hexane/ethyl acetate 9:1), ^{1}H NMR (300 MHz, chloroform-*d*) δ 7.30 (d, *J* = 8.4 Hz, 1H), 7.19 (m, 3H), 5.71 (dd, *J* = 10.8, 4.5 Hz, 1H), 3.94 (t, *J* = 6.8 Hz, 2H), 3.67 – 3.45 (m, 3H), 3.29 (dd, *J* = 16.5, 10.7 Hz, 1H), 2.51 (s, 3H), 2.11 (s, 3H), 2.08 – 1.91 (m, 4H). ^{13}C NMR (75 MHz, chloroform-*d*) δ 206.0, 191.3, 137.0, 136.3, 130.9, 127.8, 126.9, 126.1, 60.4, 54.8, 50.5, 49.3, 46.7, 29.6, 26.0, 24.2, 21.1, 19.7, 14.2. MS: (EI, 70 eV): m/z = 307 [M^+], Anal. Calcd for $C_{16}H_{21}NOS_2$: C 62.50; H 6.88; N 4.56; Found: C 62.35; H 6.63; N 4.49.

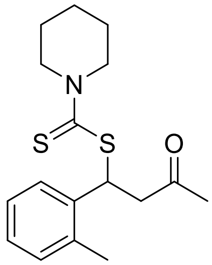

3-Oxo-1-(*o*-tolyl)butyl piperidine-1-carbodithioate (**6f**), yellow oil, isolated yield (84%, 27.31 mg), purification by preparative TLC (*n*-hexane/ethyl acetate 9:1), ^{1}H NMR (300 MHz, chloroform-*d*) δ 7.28 (s, 2H), 7.20 (m, 2H), 5.70 (dd, *J* = 10.9, 4.4 Hz, 1H), 4.29 (s, 2H), 3.82 (s, 2H), 3.55 (dd, *J* = 16.5, 4.4 Hz, 1H), 3.29 (dd, *J* = 16.5, 10.9 Hz, 1H), 2.51 (s, 3H), 2.12 (s, 3H), 1.72 (s, 6H). ^{13}C NMR (75 MHz, chloroform-*d*) δ 206.0, 194.1, 137.1, 136.3, 130.9, 127.8, 126.9, 126.1, 49.4, 47.5, 29.6, 24.2, 19.8. MS: (EI, 70 eV): m/z = 321 [M^+], Anal. Calcd for $C_{17}H_{23}NOS_2$: C 63.51; H 7.21; N 4.36; Found: C 63.37; H 7.33; N 4.10.

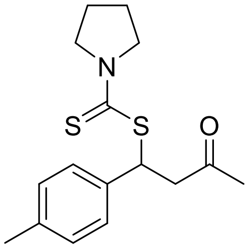

3-Oxo-1-(*p*-tolyl)butyl pyrrolidine-1-carbodithioate (6g), yellow oil, isolated yield (85%, 26.43 mg), purification by preparative TLC (*n*-hexane/ethyl acetate 9:1), ^{1}H NMR (300 MHz, chloroform-*d*) δ 7.31 (d, *J* = 7.8 Hz, 2H), 7.13 (d, *J* = 7.6 Hz, 2H), 5.56 (dd, *J* = 10.1, 5.0 Hz, 1H), 3.92 (t, *J* = 6.8 Hz, 2H), 3.62 – 3.45 (m, 3H), 3.16 (dd, *J* = 16.2, 10.2 Hz, 1H), 2.32 (s, 3H), 2.14 (s, 3H), 2.01 (dq, *J* = 19.5, 6.7 Hz, 4H). ^{13}C NMR (75 MHz, chloroform-*d*) δ 205.9, 191.1, 137.6, 136.0, 129.4, 128.1, 54.8, 50.5, 50.1, 49.9, 29.8, 26.0, 24.2, 21.1. MS: (EI, 70 eV): m/z = 307 [M^+], Anal. Calcd for $C_{16}H_{21}NOS_2$: C 62.50; H 6.88; N 4.56; Found: C 62.41; H 6.52; N 4.29.

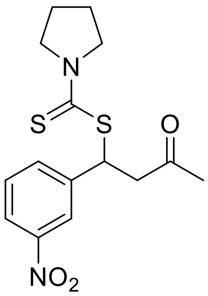

1-(3-Nitrophenyl)-3-oxobutyl pyrrolidine-1-carbodithioate (6h), brown oil, isolated yield (69%, 23.36 mg), purification by preparative TLC (*n*-hexane/ethyl acetate 8:1), ^{1}H NMR (300 MHz, chloroform-*d*) δ 8.28 (s, 1H), 8.11 (d, *J* = 8.5 Hz, 1H), 7.84 (d, *J* = 7.9 Hz, 1H), 7.50 (q, *J* = 8.0, 6.9 Hz, 1H), 5.73 (dd, *J* = 9.2, 5.1 Hz, 1H), 3.91 (t, *J* = 6.9 Hz, 2H), 3.61 (t, *J* = 6.9 Hz, 2H), 3.48 (dd, *J* = 17.4, 5.1 Hz, 1H), 3.23 (dd, *J* = 17.4, 9.2 Hz, 1H), 2.17 (s, 3H), 2.13 – 1.97 (m, 4H). ^{13}C NMR (75 MHz, chloroform-*d*) δ 204.7, 189.7, 148.2, 142.5, 135.2, 129.3, 123.1, 122.6, 55.1, 50.6, 49.3, 49.1, 30.0, 26.0, 24.2. MS: (EI, 70 eV): m/z = 338 [M^+], Anal. Calcd for $C_{15}H_{18}N_2O_3S_2$: C 53.23; H 5.36; N 8.28; Found: C 53.11; H 5.22; N 8.29.

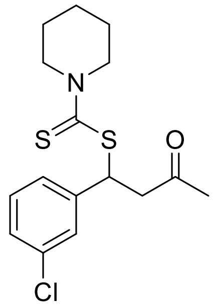

1-(3-Chlorophenyl)-3-oxobutyl piperidine-1-carbodithioate (6i), brown oil, isolated yield (81%, 28.7 mg), purification by preparative TLC (*n*-hexane/ethyl acetate 8:1), ^{1}H NMR (300 MHz, chloroform-*d*) δ 7.41 (s, 1H), 7.27 (m, 3H), 5.58 (dd, *J* = 9.9, 4.8 Hz, 1H), 4.47 – 4.14 (m, 2H), 3.84 (s, 2H), 3.49 (dd, *J* = 16.8, 4.8 Hz, 1H), 3.15 (dd, *J* = 16.8, 9.9 Hz, 1H), 2.16 (s, 3H), 1.71 (s, 6H). ^{13}C NMR (75 MHz, chloroform-*d*) δ 205.3, 193.1, 141.6, 134.4, 129.8, 128.5, 127.9, 126.7, 50.4, 49.80, 29.9, 24.2. MS: (EI, 70 eV): m/z = 341 [M^+], Anal. Calcd for $C_{16}H_{20}ClNOS_2$: C 52.21; H 5.90; N 4.10; Found: C 52.01; H 5.97; N 4.02.

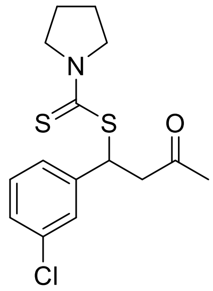

1-(3-Chlorophenyl)-3-oxobutyl pyrrolidine-1-carbodithioate (6j), brown oil, isolated yield (88%, 29.9 mg), purification by preparative TLC (*n*-hexane/ethyl acetate 8:1), ^{1}H NMR (300 MHz, chloroform-*d*) δ 7.40 (s, 1H), 7.37 – 7.18 (m, 4H), 5.60 (dd, *J* = 9.5, 5.1 Hz, 1H), 3.91 (t, *J* = 6.9 Hz, 2H), 3.58 (t, *J* = 6.5 Hz, 2H), 3.45 (dd, *J* = 16.8, 5.1 Hz, 1H), 3.15 (dd, *J* = 16.9, 9.5 Hz, 1H), 2.15 (s, 3H), 2.10 – 1.94 (m, 4H). ^{13}C NMR (75 MHz, chloroform-*d*) δ 205.2, 190.4, 141.8, 134.3, 129.8, 128.4, 127.9, 126.6, 55.0, 50.6, 49.6, 49.6, 29.9, 26.0, 24.2. MS: (EI, 70 eV): m/z = 327 [M^+], Anal. Calcd for $C_{15}H_{18}ClNOS_2$: C 54.95; H 5.53; N 4.27; Found: C 54.91; H 5.22; N 4.54.

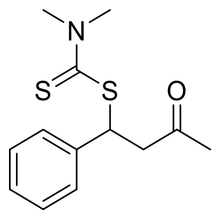

3-Oxo-1-phenylbutyl dimethylcarbamodithioate (6k), yellow oil, isolated yield (90%, 24.06 mg), purification by preparative TLC (*n*-hexane/ethyl acetate 9:1), ^{1}H NMR (300 MHz, chloroform-*d*) δ 7.42 (d, *J* = 6.6 Hz, 2H), 7.32 (dd, *J* = 15.1, 7.9 Hz, 3H), 5.54 (dd, *J* = 10.0, 4.9 Hz, 1H), 3.52 (d, *J* = 16.9 Hz, 4H), 3.32 (s, 3H), 3.18 (dd, *J* = 16.3, 10.0 Hz, 1H), 2.15 (s, 3H). ^{13}C NMR (75 MHz, chloroform-*d*) δ 205.7, 195.3, 139.0, 128.7, 128.3, 127.8, 51.5, 49.8, 45.2, 41.4, 29.8. MS: (EI, 70 eV): m/z = 267 [M^+], Anal. Calcd for $C_{13}H_{17}NOS_2$: C 58.39; H 6.41; N 4.24; Found: C 58.61; H 6.09; N 4.68.

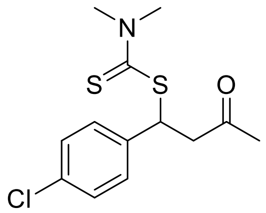

1-(4-Chlorophenyl)-3-oxobutyl dimethylcarbamodithioate (6l), brown oil, isolated yield (85%, 27.2 mg), purification by preparative TLC (*n*-hexane/ethyl acetate 8:1), ^{1}H NMR (300 MHz, chloroform-*d*) δ 7.38 (d, *J* = 8.3 Hz, 2H), 7.30 (d, *J* = 8.3 Hz, 2H), 5.52 (dd, *J* = 9.8, 5.0 Hz, 1H), 3.54 (s, 3H), 3.47 (dd, *J* = 16.9, 4.8 Hz, 1H), 3.33 (s, 3H), 3.14 (dd, *J* = 16.8, 9.8 Hz, 1H), 2.15 (s, 3H). ^{13}C NMR (75 MHz, chloroform-*d*) δ 205.30, 133.5, 129.7, 128.8, 77.4, 77.0, 76.6, 50.7, 49.6, 45.2, 41.4, 29.9. MS: (EI, 70 eV): m/z = 301.04 [M^+], Anal. Calcd for $C_{13}H_{16}ClNOS_2$: C 51.73; H 5.34; N 4.64; Found: C 51.81; H 5.30; N 4.79.

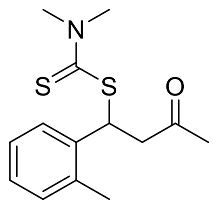

3-Oxo-1-(*o*-tolyl)butyl dimethylcarbamodithioate (6m), yellow oil, isolated yield (89%, 25.04 mg), purification by preparative TLC (*n*-hexane/ethyl acetate 9:1), ^{1}H NMR (300 MHz, chloroform-*d*) δ 7.34 – 7.27 (m, 1H), 7.24 – 7.14 (m, 3H), 5.64 (dd, *J* = 10.8, 4.5 Hz, 1H), 3.53 (m, 4H), 3.35 – 3.21 (m, 4H), 2.50 (s, 3H), 2.12 (s, 3H). ^{13}C NMR (75 MHz, chloroform-*d*) δ 205.9, 195.7, 137.1, 136.2, 130.9, 130.2, 127.8, 126.9, 126.4, 126.1, 77.2, 49.1, 48.1, 45.14, 41.3, 29.6, 19.7. MS: (EI, 70 eV): m/z = 281 [M^{+}], Anal. Calcd for $C_{14}H_{19}NOS_2$: C 59.75; H 6.81; N 4.98; Found: C 59.31; H 6.12; N 4.89.

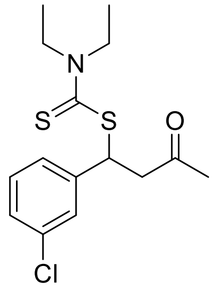

1-(3-Chlorophenyl)-3-oxobutyl diethylcarbamodithioate (6n), brown oil, isolated yield (87%, 29.7 mg), purification by preparative TLC (*n*-hexane/ethyl acetate 8:1), ^{1}H NMR (300 MHz, chloroform-*d*) δ 7.53 (s, 1H), 7.40 (m, 4H), 5.54 (dd, *J* = 9.9, 4.8 Hz, 1H), 4.02 (m, 4H), 3.47 (dd, *J* = 16.8, 4.8 Hz, 1H), 3.14 (dd, *J* = 16.8, 9.9 Hz, 1H), 2.39 (s, 3H), 1.27 (m, 6H). ^{13}C NMR (75 MHz, chloroform-*d*) δ 205.28, 193.2, 141.6, 134.3, 129.8, 128.1, 127.9, 126.4, 50.3, 49.4, 46.7, 29.9, 27.8, 12.5, 11.6. MS: (EI, 70 eV): m/z = 329 [M^{+}], Anal. Calcd for $C_{15}H_{20}ClNOS_2$: C 54.61; H 6.11; N 4.25; Found: C 54.81; H 6.02; N 4.69.

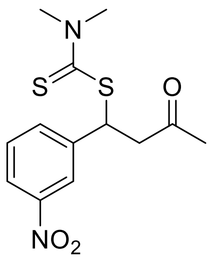

1-(3-Nitrophenyl)-3-oxobutyl dimethylcarbamodithioate (6o), brown oil, isolated yield (76%, 26.5 mg), purification by preparative TLC (*n*-hexane/ethyl acetate 8:1), ^{1}H NMR (300 MHz, chloroform-*d*) δ 8.27 (s, 1H), 8.11 (d, *J* = 8.2 Hz, 1H), 7.83 (d, *J* = 7.8 Hz, 1H), 7.49 (t, *J* = 8.0 Hz, 1H), 5.65 (dd, *J* = 9.3, 5.1 Hz, 1H), 3.48 (m, 4H), 3.34 (s, 3H), 3.22 (dd, *J* = 17.4, 9.3 Hz, 1H), 2.16 (s, 3H). ^{13}C NMR (75 MHz, chloroform-*d*) δ 204.6, 194.1, 148.2, 142.4, 135.2, 129.3, 123.0, 122.6, 77.5, 77.0, 76.6, 50.3, 49.2, 45.4, 41.5, 30.0. MS: (EI, 70 eV): m/z = 312 [M^+], Anal. Calcd for $C_{13}H_{16}N_2O_3S_2$: C 49.98; H 5.16; N 8.97; Found: C 50. 20; H 5.72; N 8.79.

References

(1) Štrukil, V.; Igrc, M.D.; Fábián, L.; Eckert-Maksić, M.; Childs, S.L.; Reid, D.G.; Duer, M.J.; Halasz, I.; Mottillo, C.; Friščić, T. *Green Chem*. **2012**, *14*, 2462.

(2) Xia, S.; Wang, X.; Ge, Z.M.; Cheng, T.M.; Li, R.T. *Tetrahedron*. **2009**, *65*, 1005.

(3) Azizi, N.; Gholibeglo, E.; Nayeri, S.D. *Monatsh. fur Chem.* **2012**, *143*, 1171.

^{1}H NMR, ^{13}C NMR {^{1}H} and mass spectra

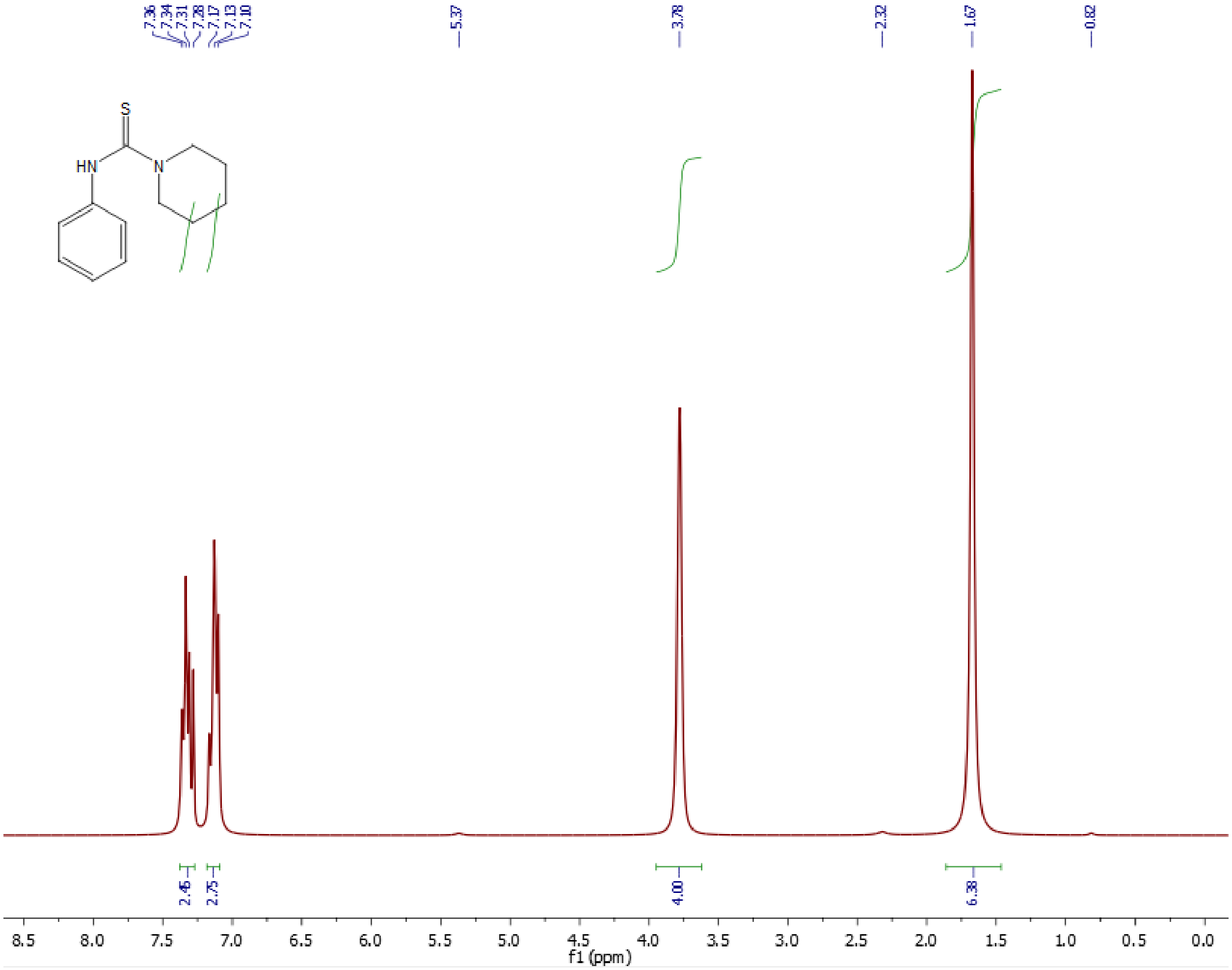

^{1}H NMR (300 MHz, chloroform-*d*) of thiourea

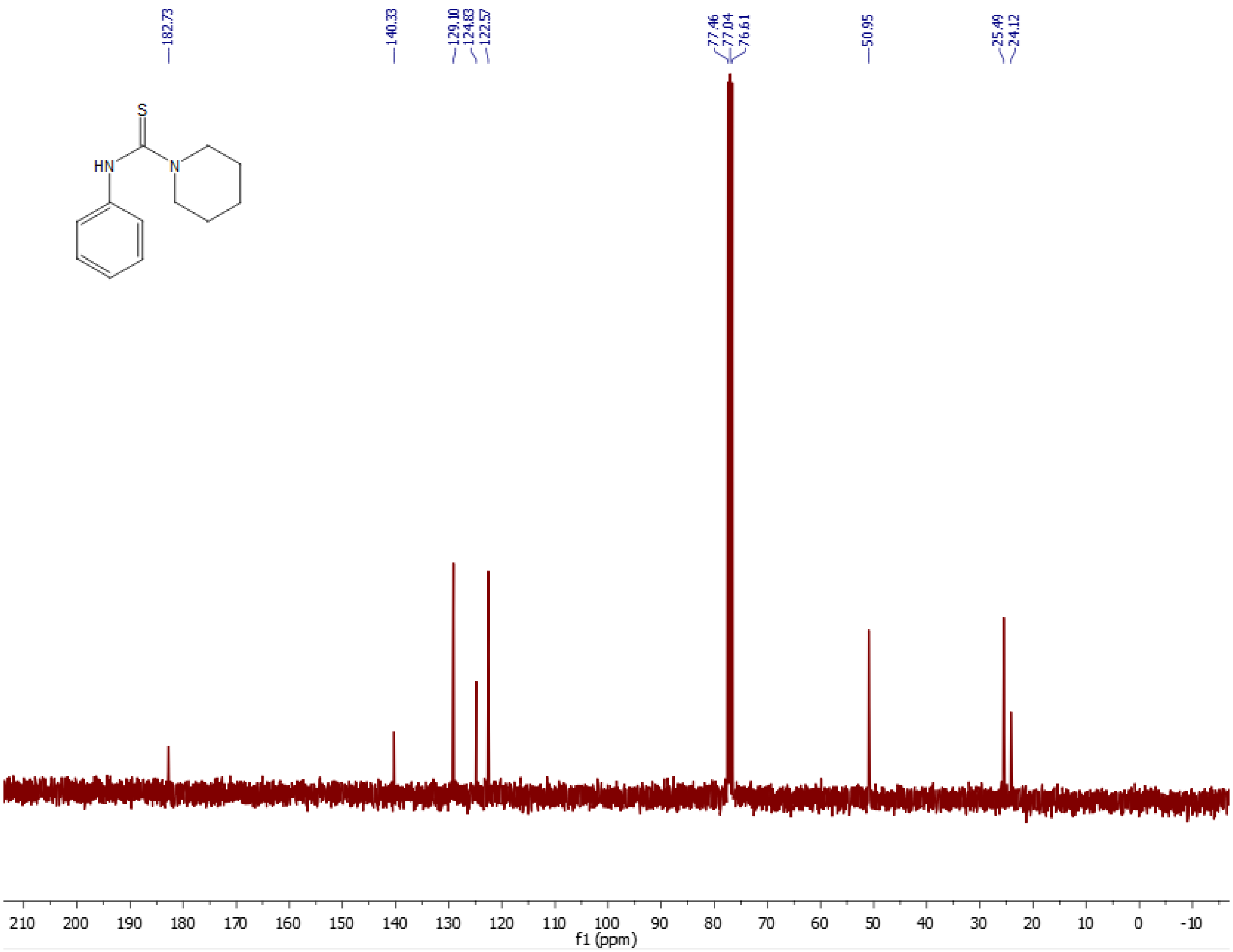

^{13}C NMR {^{1}H} (75 MHz, chloroform-*d*) of thiourea

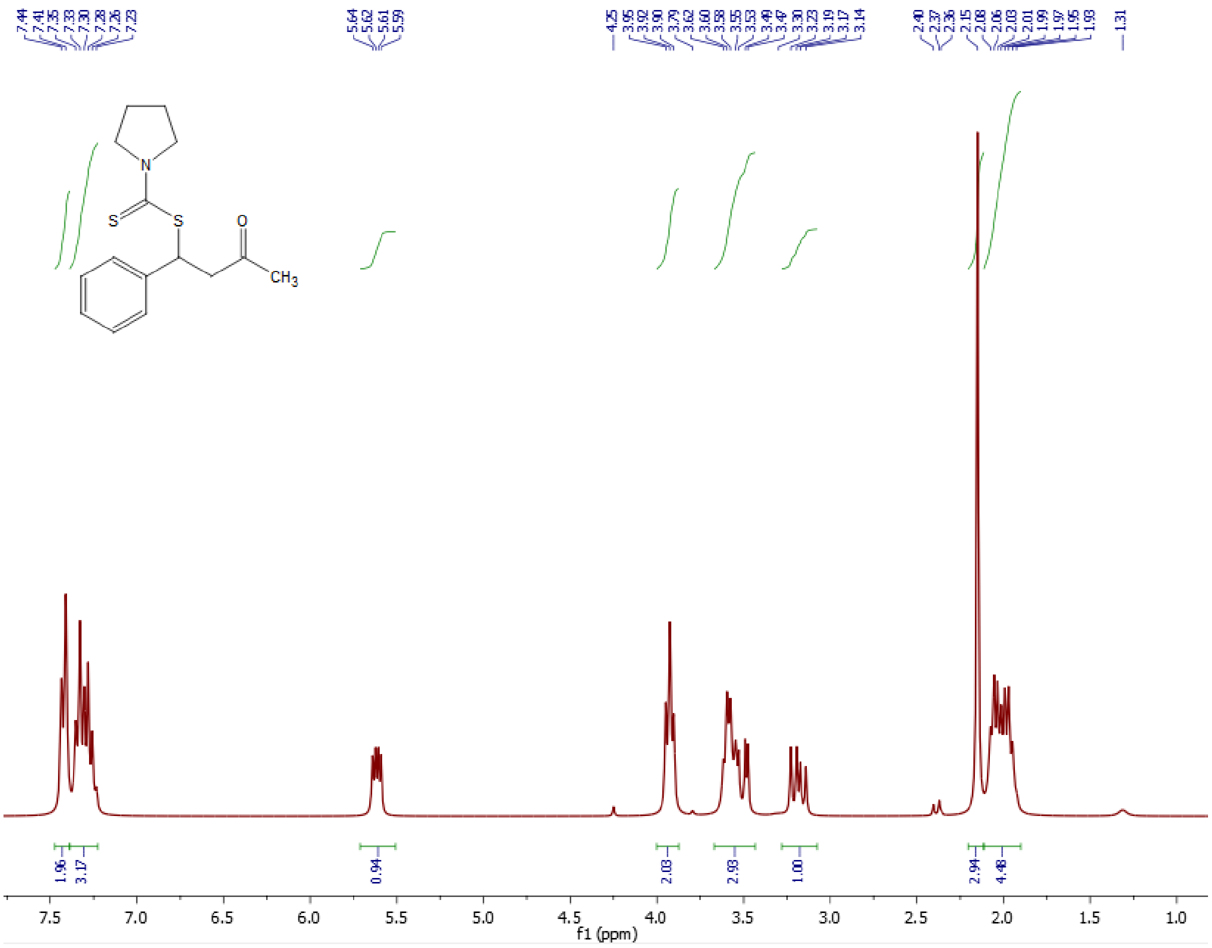

^{1}H NMR (300 MHz, chloroform-*d*) of compound **6a**

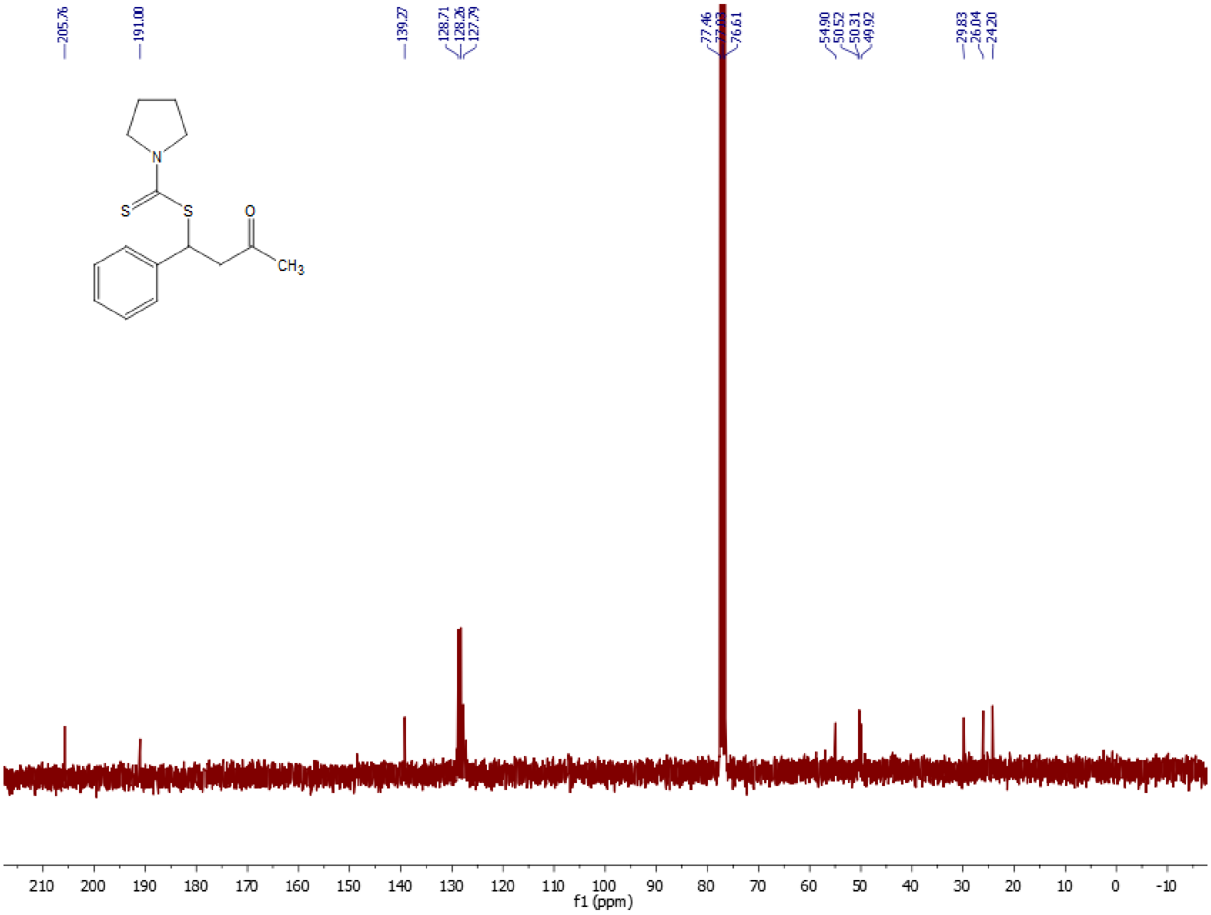

^{13}C NMR {^{1}H} (75 MHz, chloroform-*d*) of compound **6a**

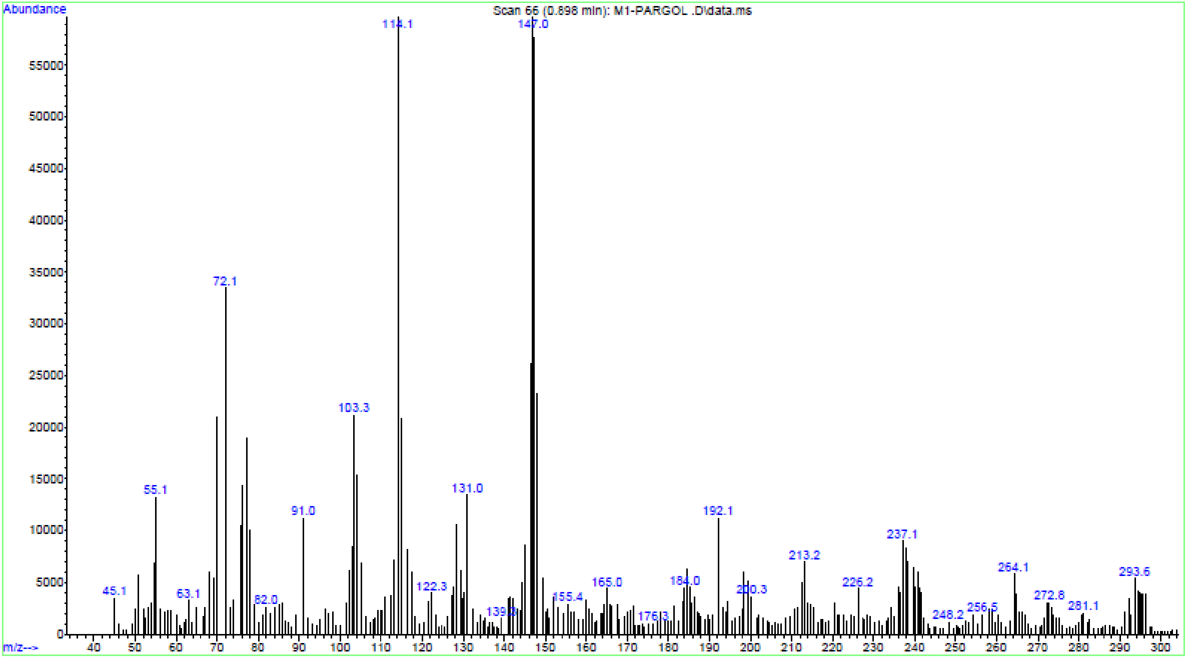

Mass spectrum of compound **6a**

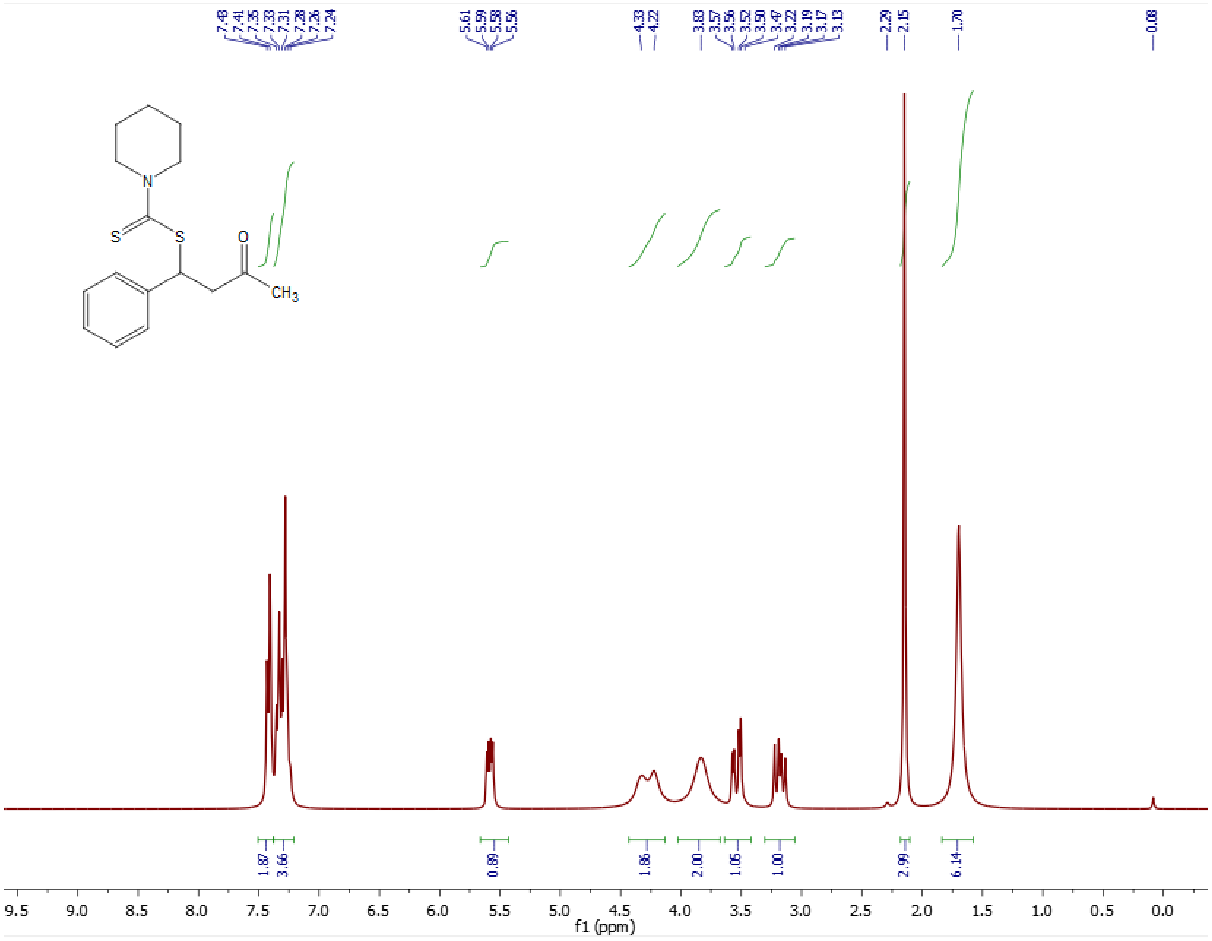

^{1}H NMR (300 MHz, chloroform-*d*) of compound **6b**

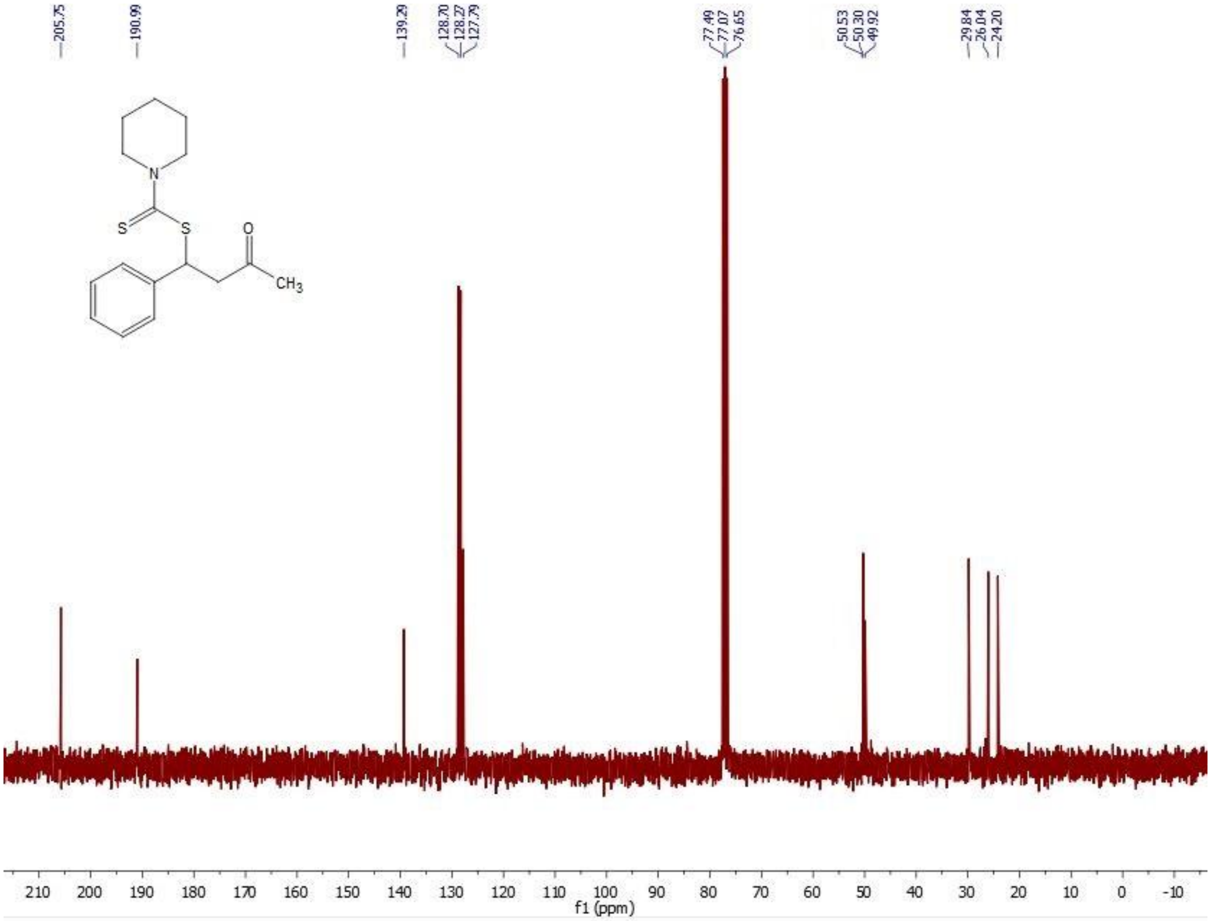

^{13}C NMR {^{1}H} (75 MHz, chloroform-*d*) of compound **6b**

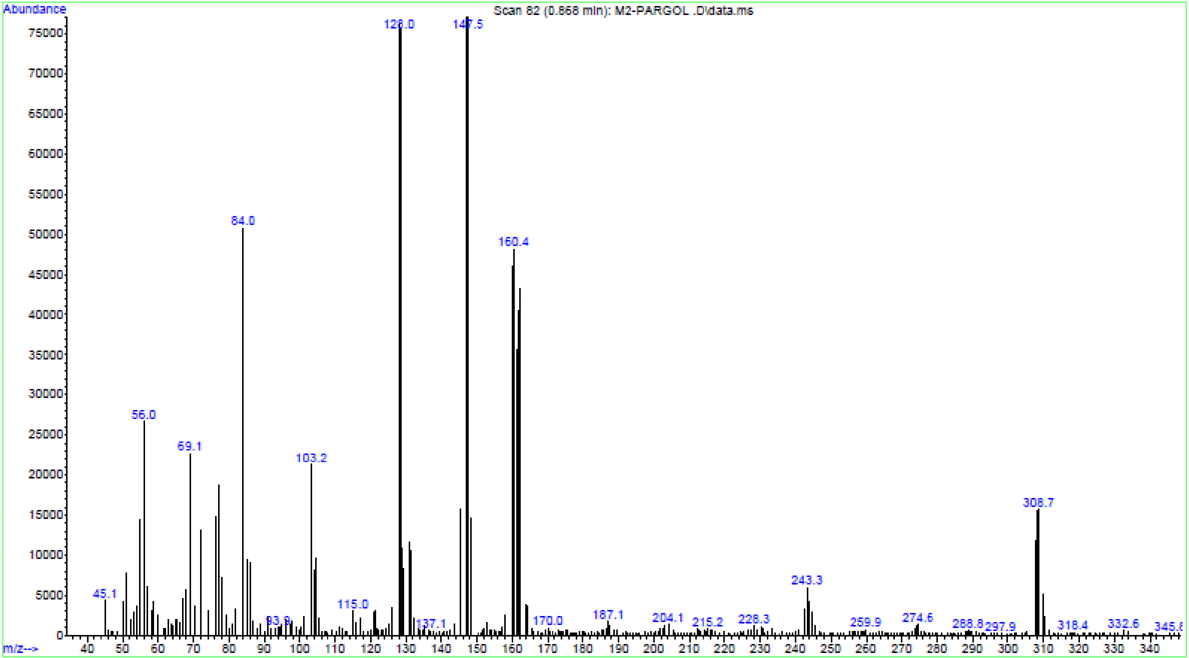

Mass spectrum of compound **6b**

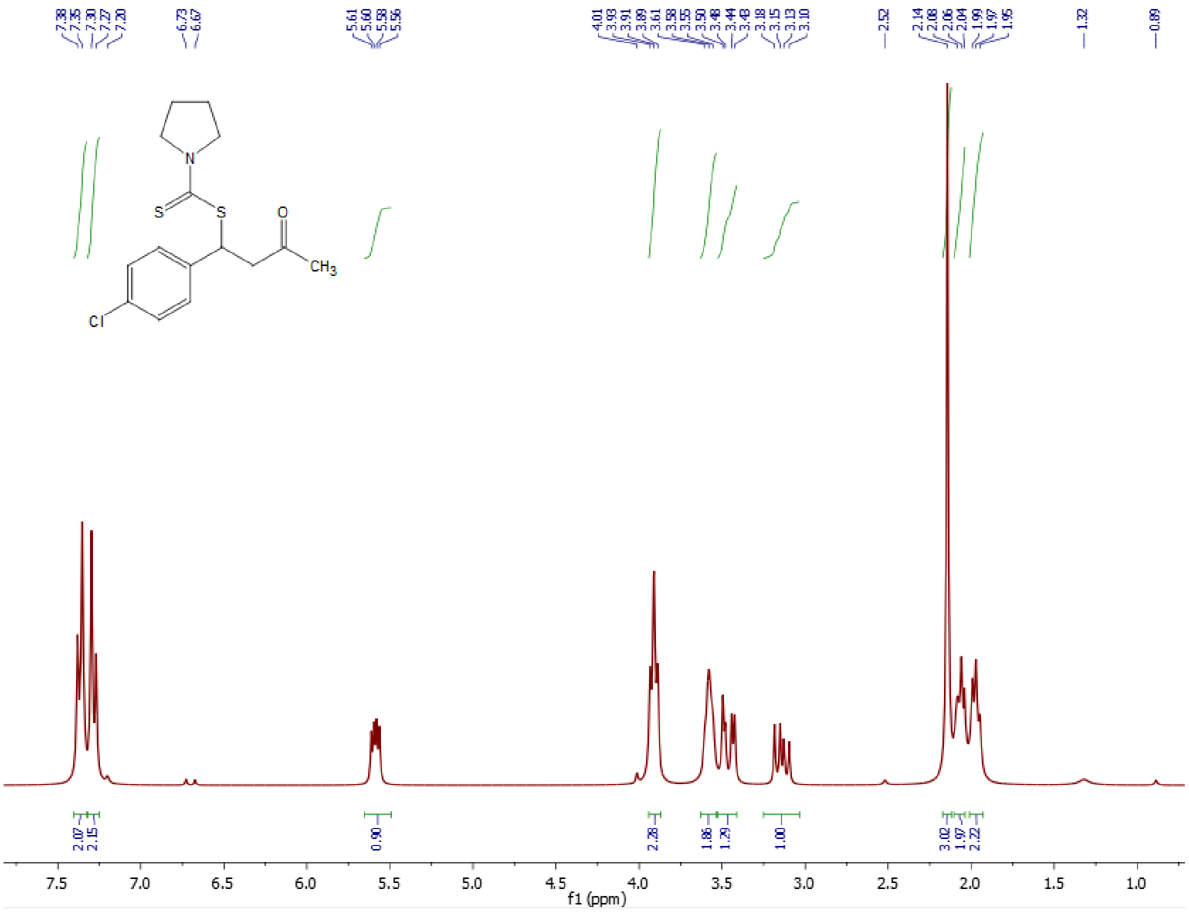

^{1}H NMR (300 MHz, chloroform-*d*) of compound **6c**

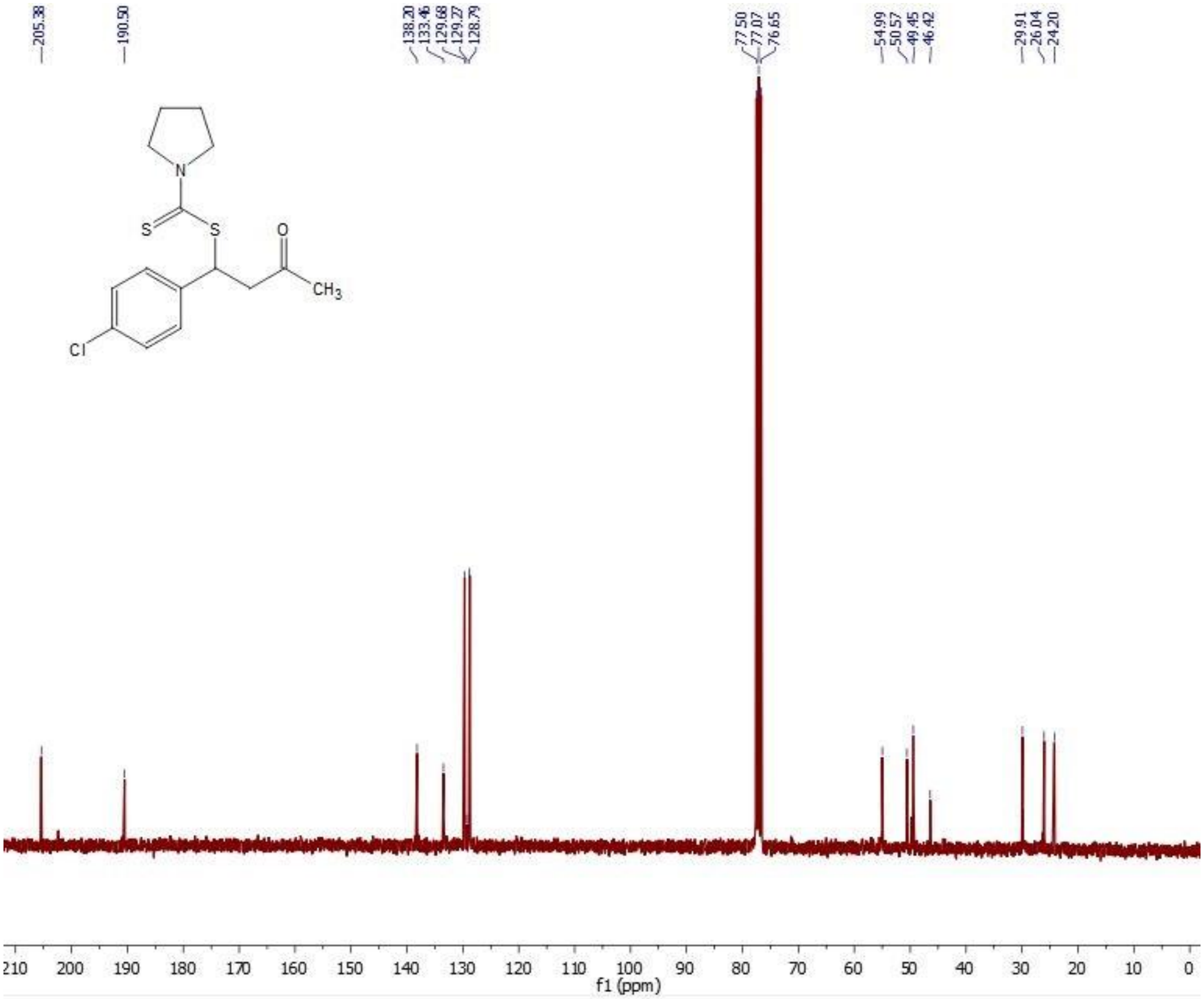

^{13}C NMR {^{1}H} (75 MHz, chloroform-*d*) of compound **6c**

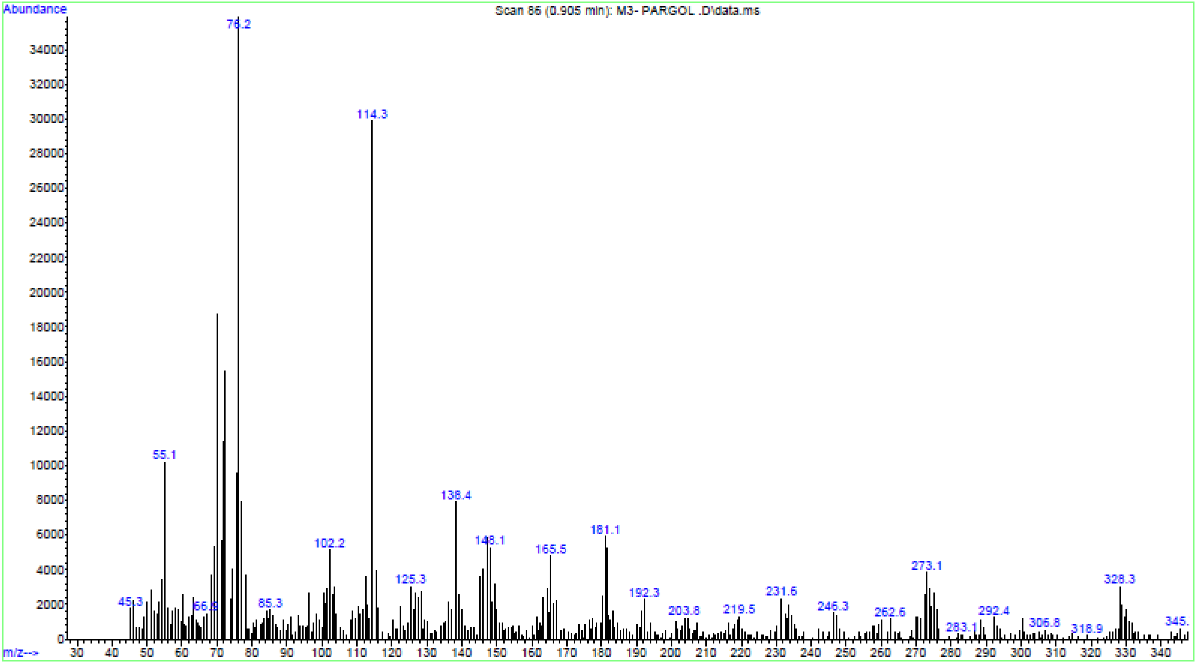

Mass spectrum of compound **6c**

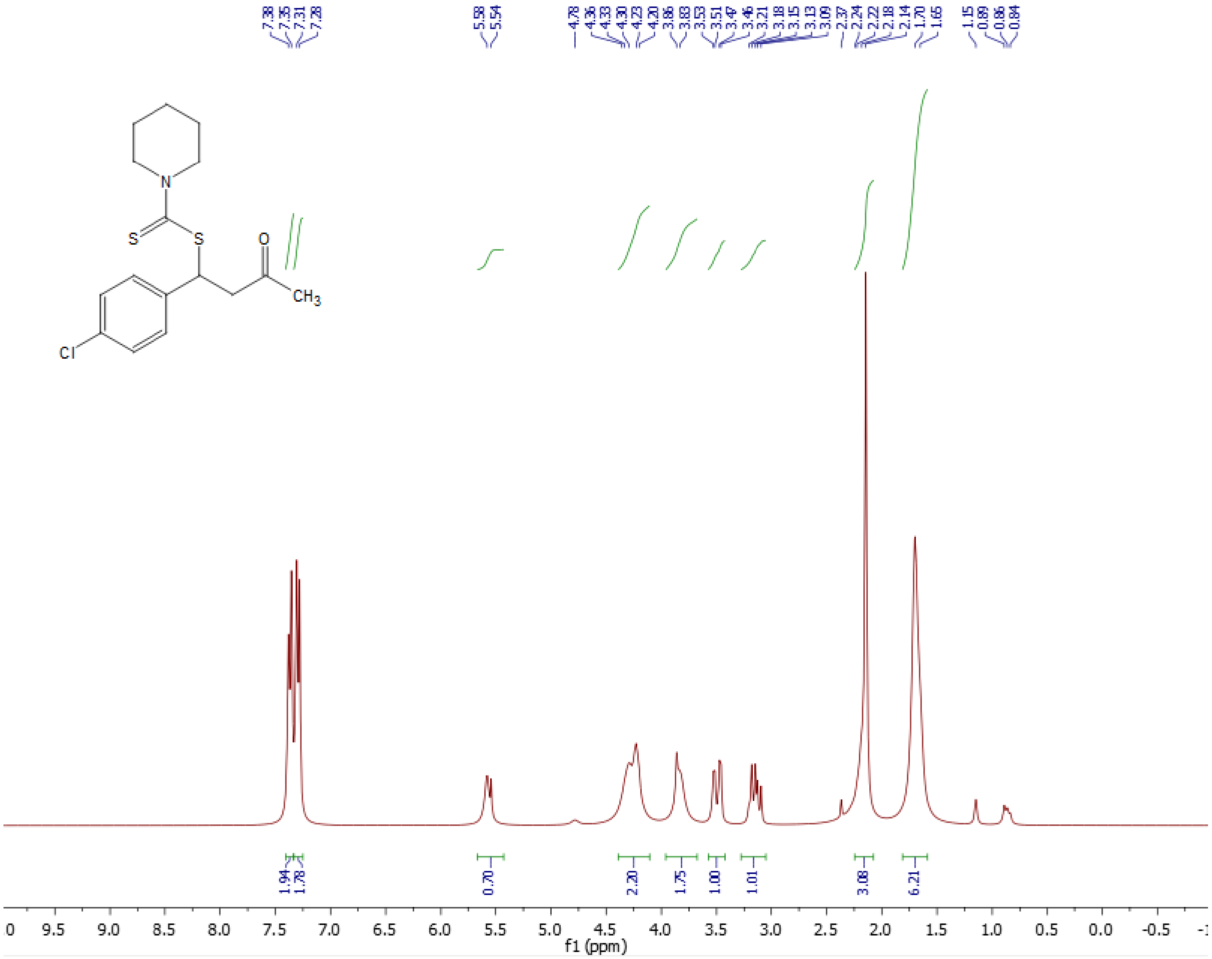

^{1}H NMR (300 MHz, chloroform-*d*) of compound **6d**

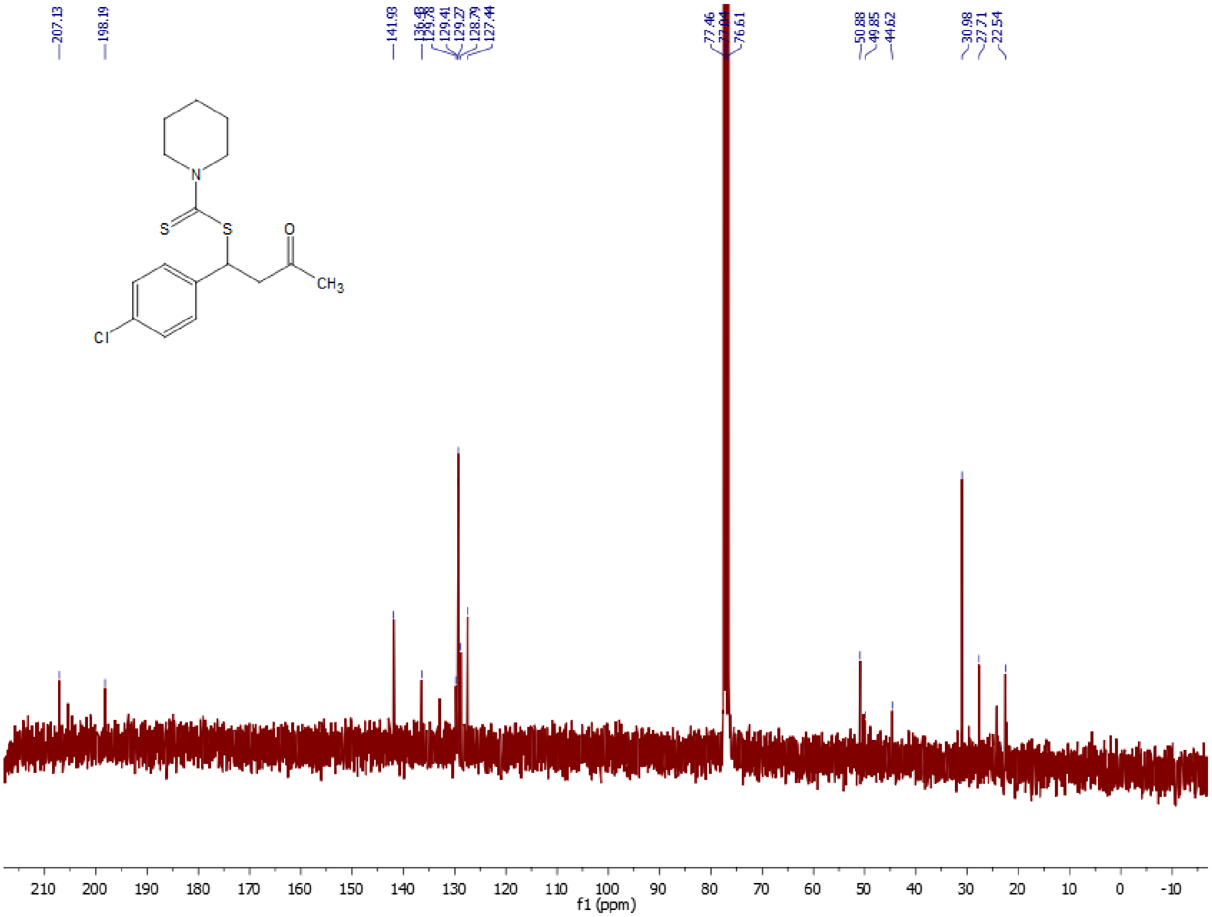

^{13}C NMR {^{1}H} (75 MHz, chloroform-*d*) of compound **6d**

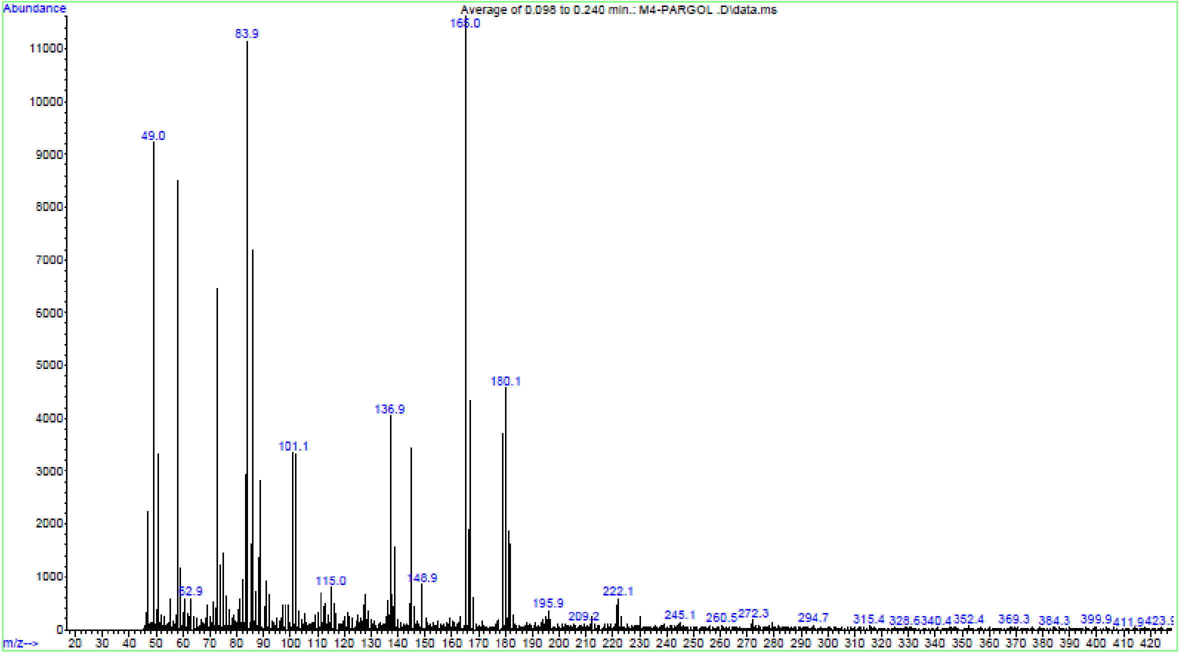

Mass spectrum of compound **6d**

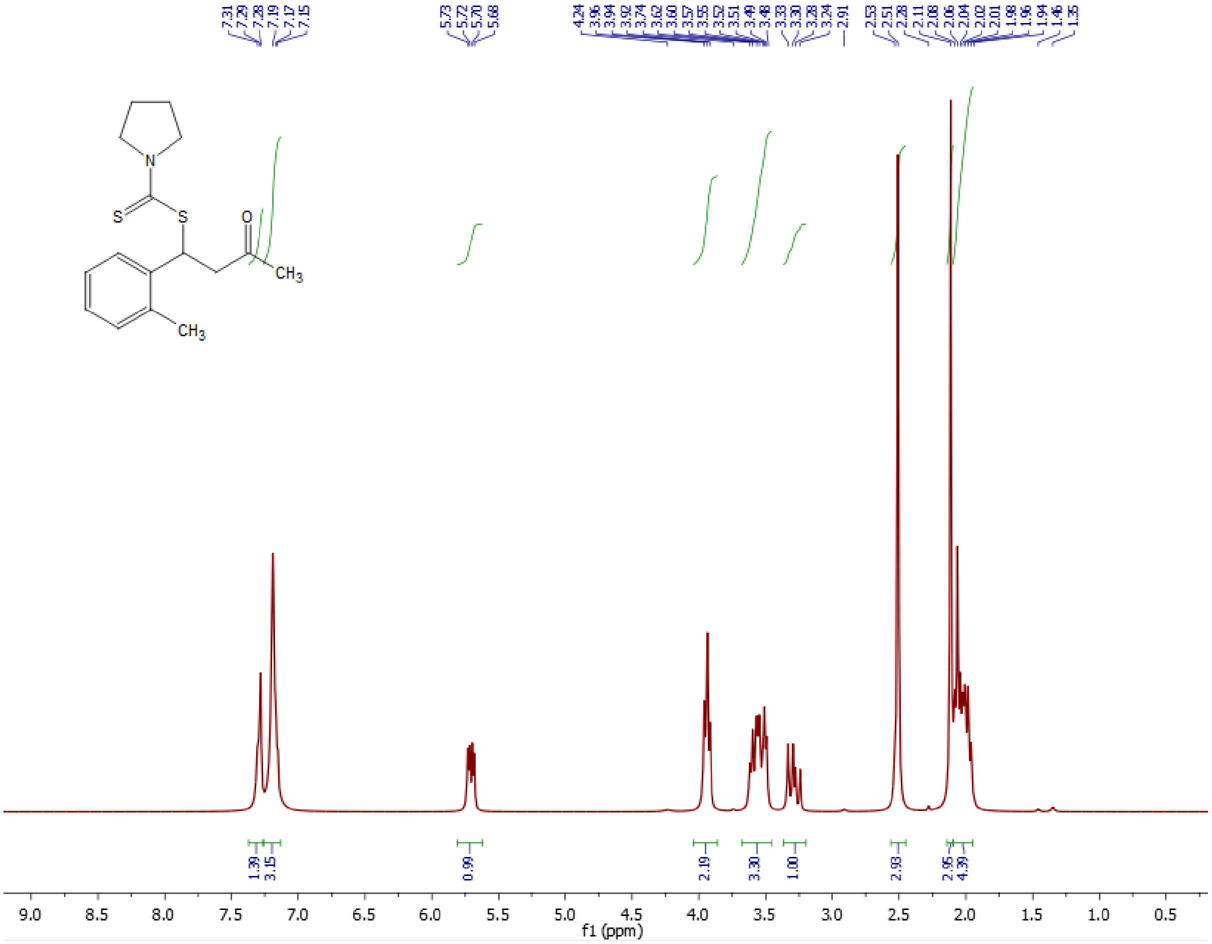

^{1}H NMR (300 MHz, chloroform-*d*) of compound **6e**

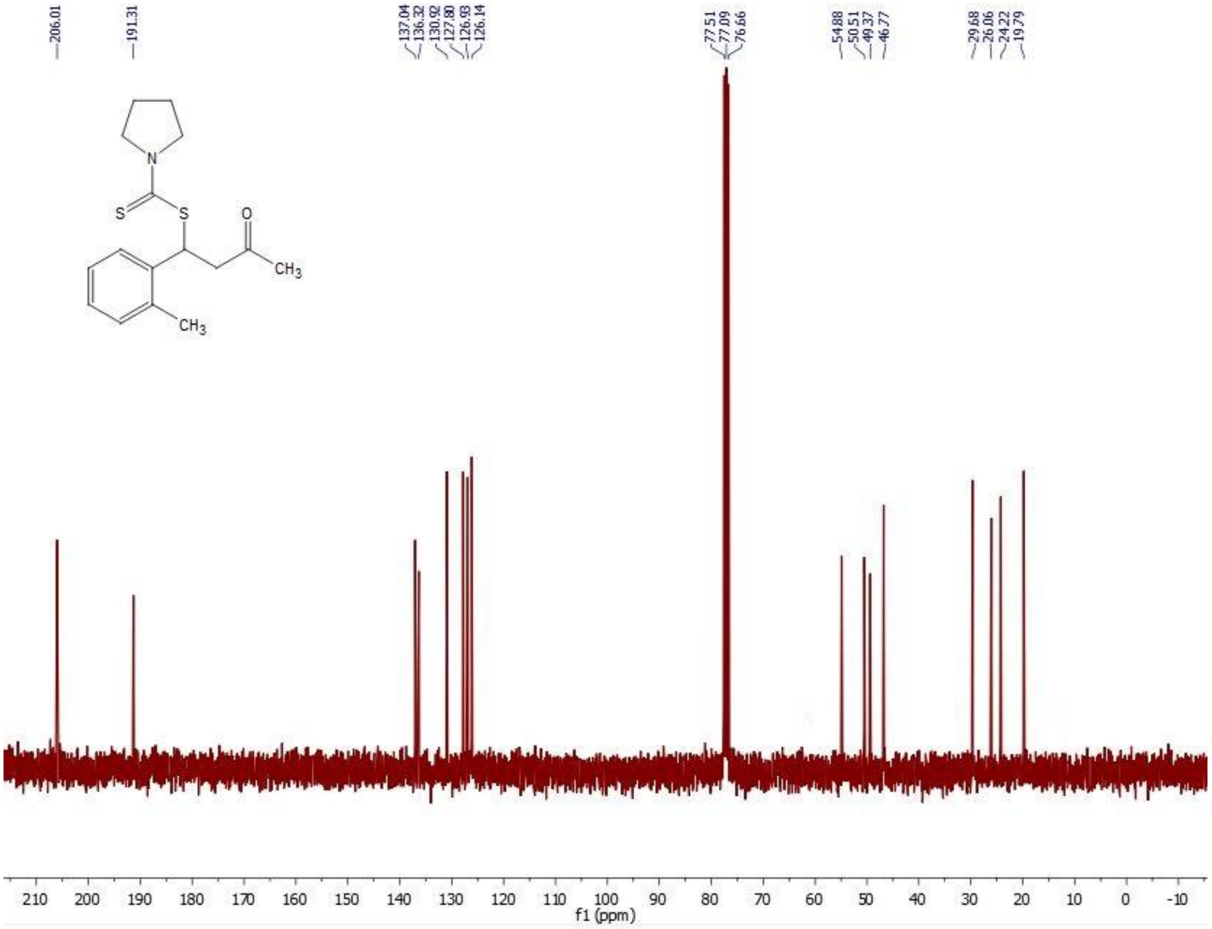

^{13}C NMR {^{1}H} (75 MHz, chloroform-*d*) of compound **6e**

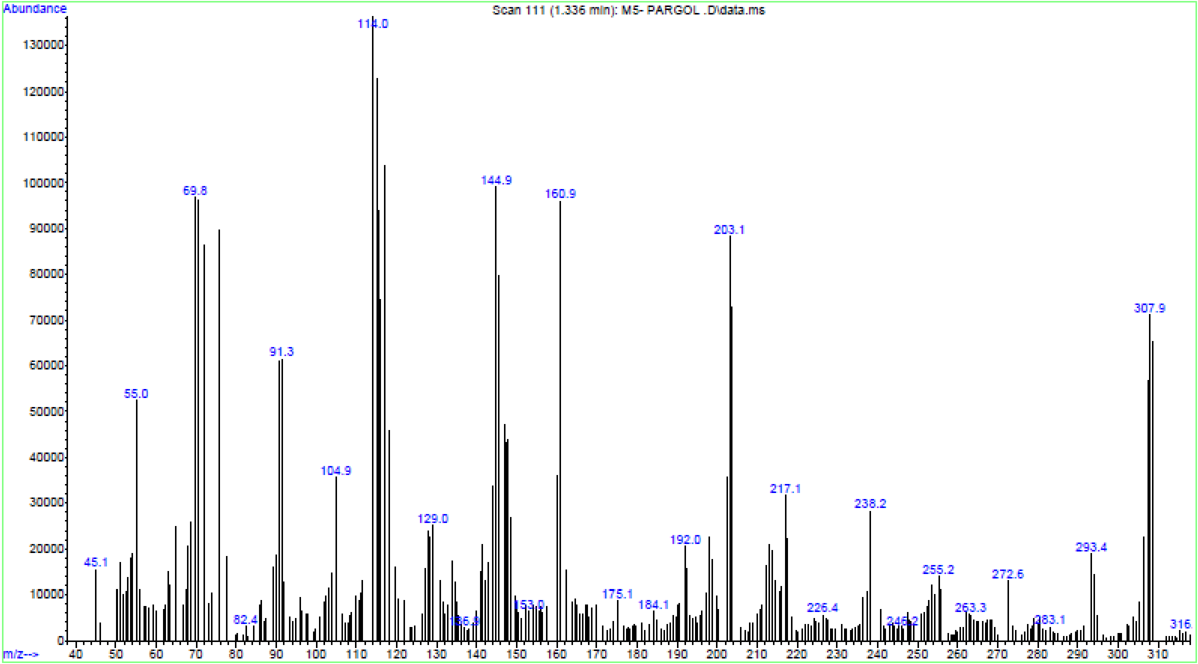

Mass spectrum of compound **6e**

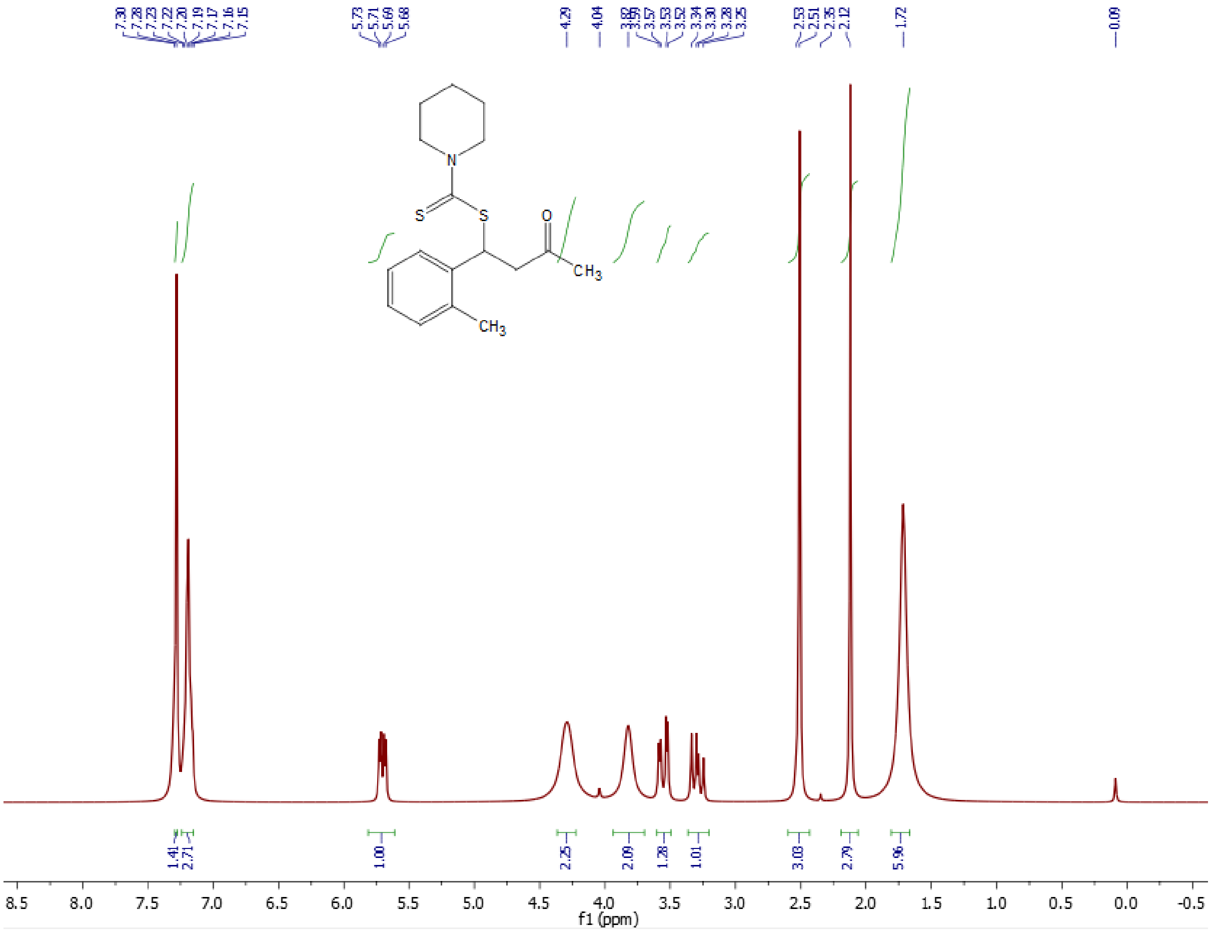

^{1}H NMR (300 MHz, chloroform-*d*) of compound **6f**

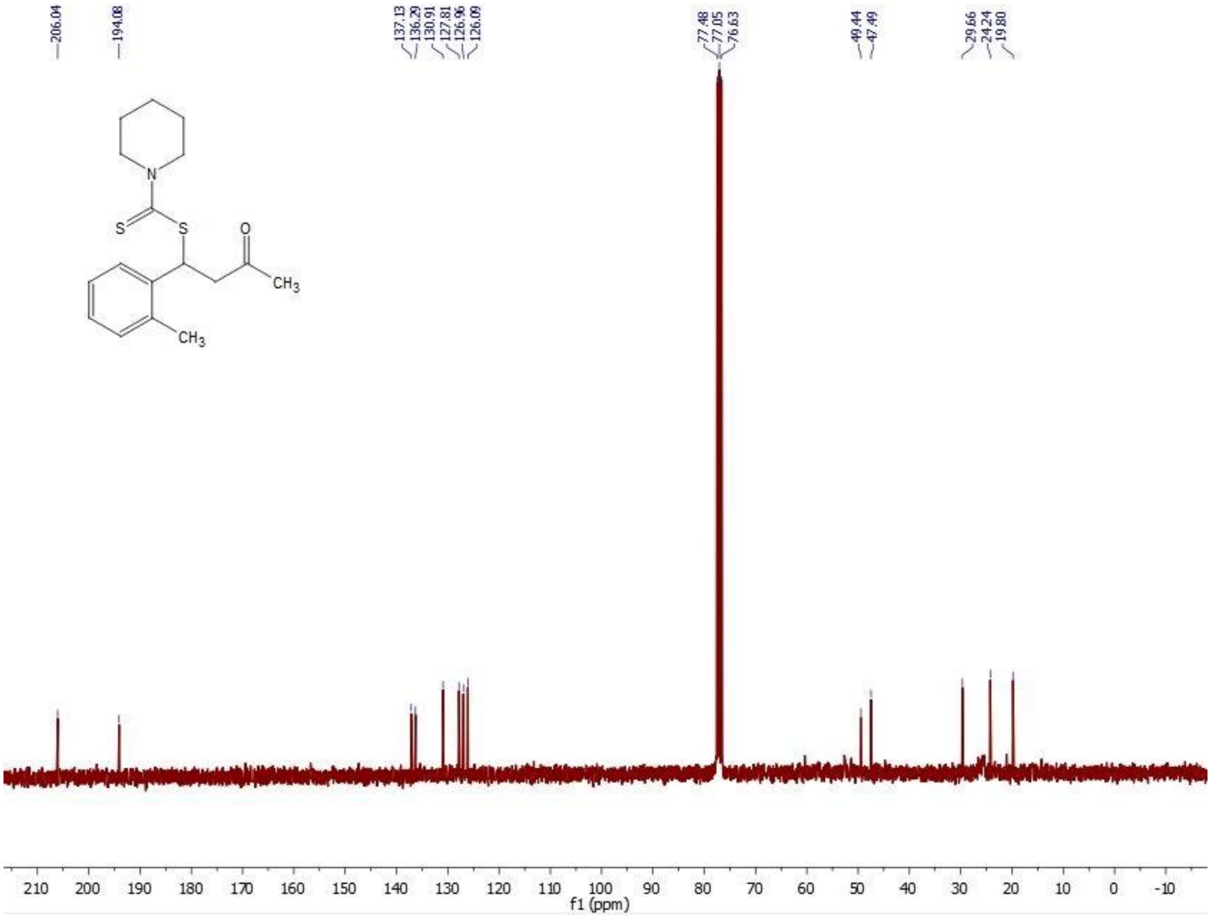

^{13}C NMR {^{1}H} (75 MHz, chloroform-*d*) of compound **6f**

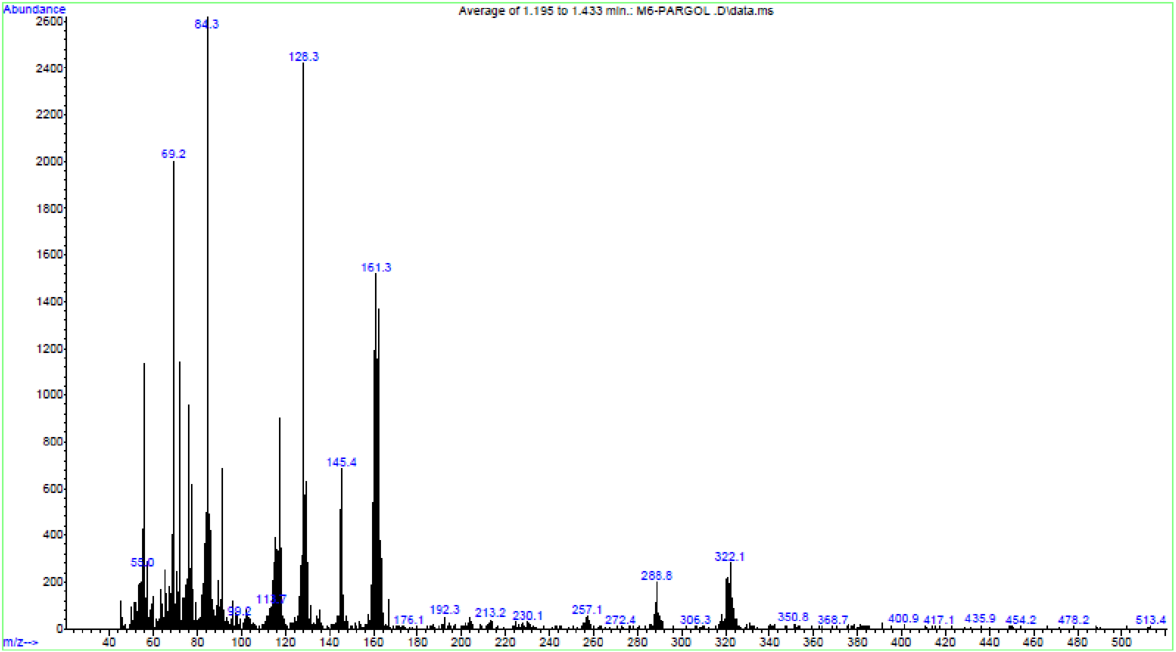

Mass spectrum of compound **6f**

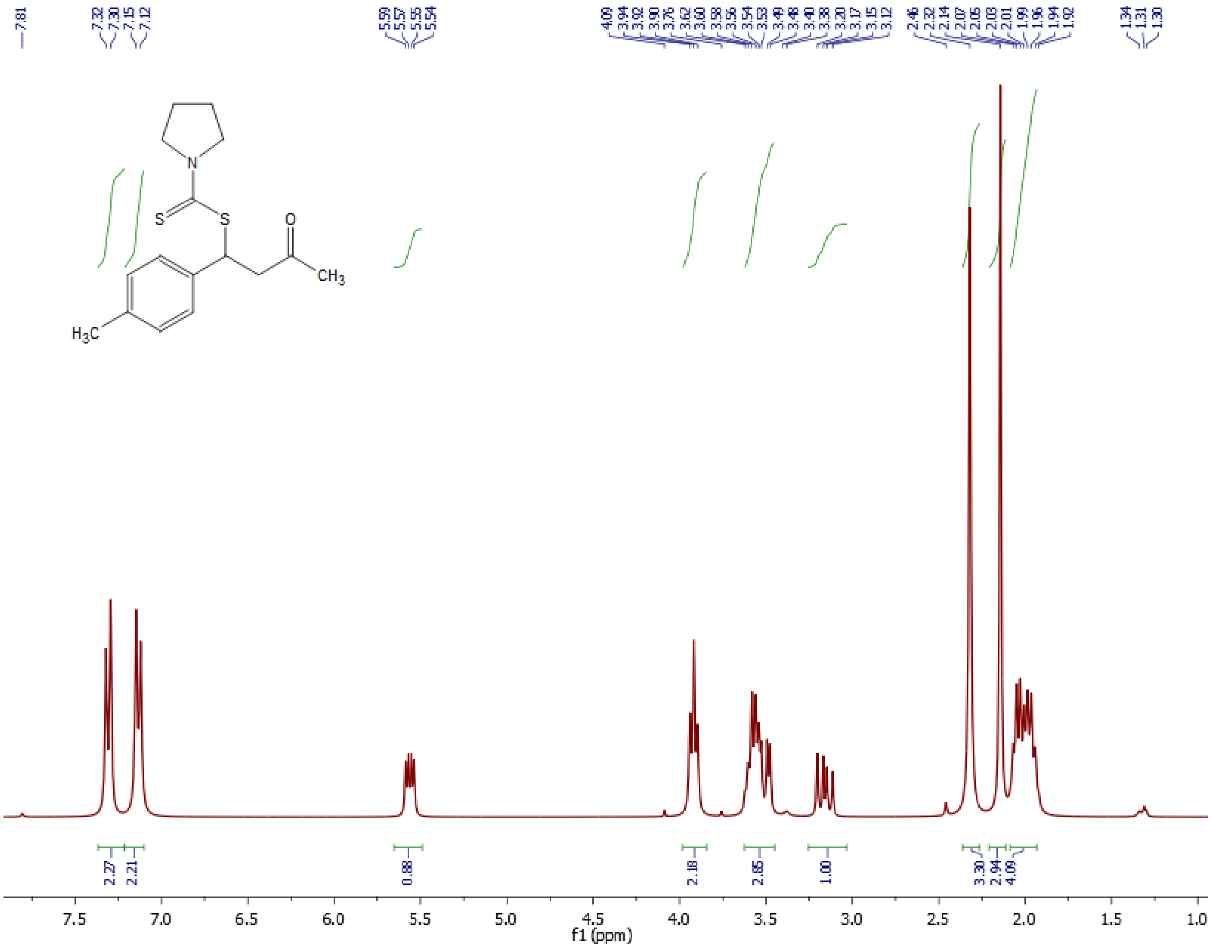

^{1}H NMR (300 MHz, chloroform-*d*) of compound **6g**

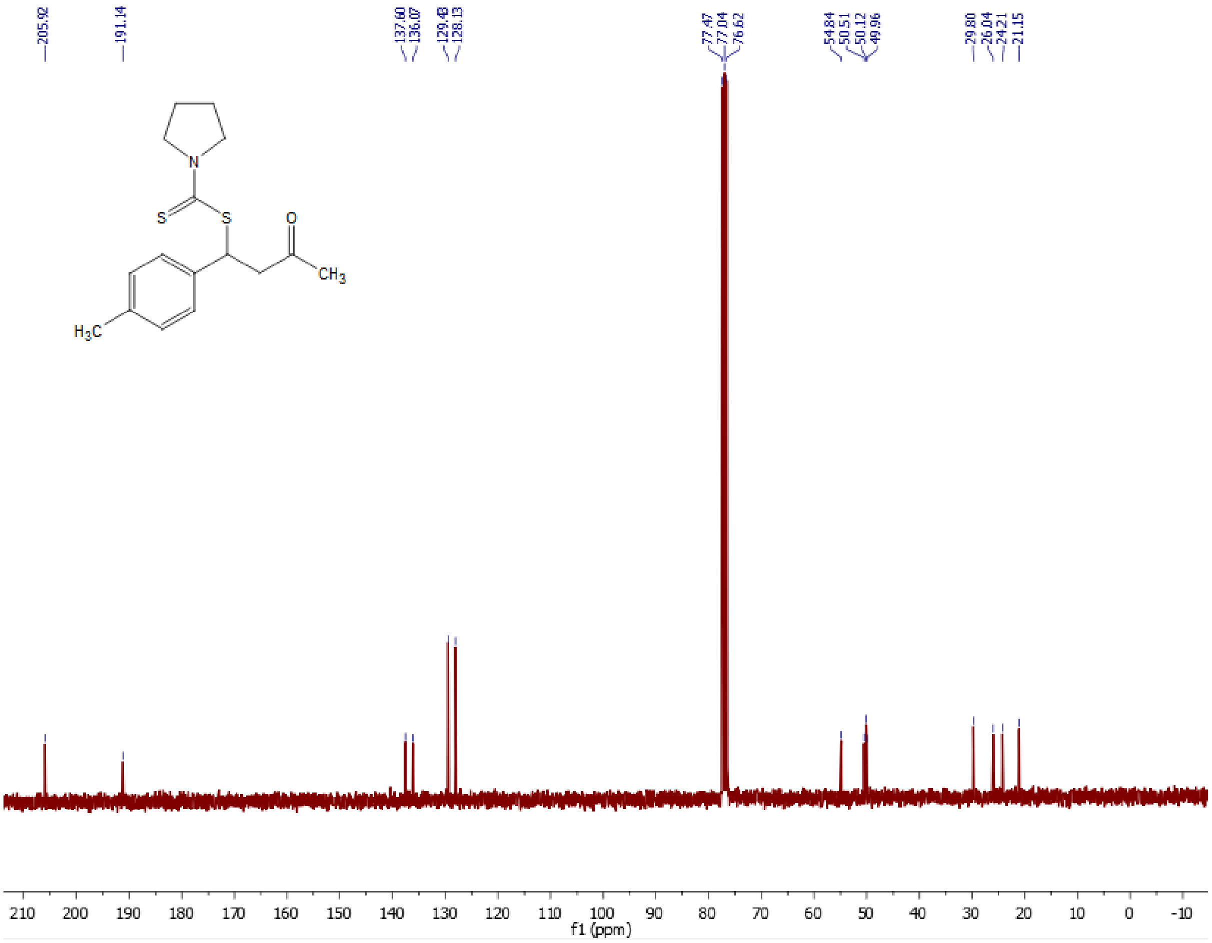

^{13}C NMR {^{1}H} (75 MHz, chloroform-*d*) of compound **6g**

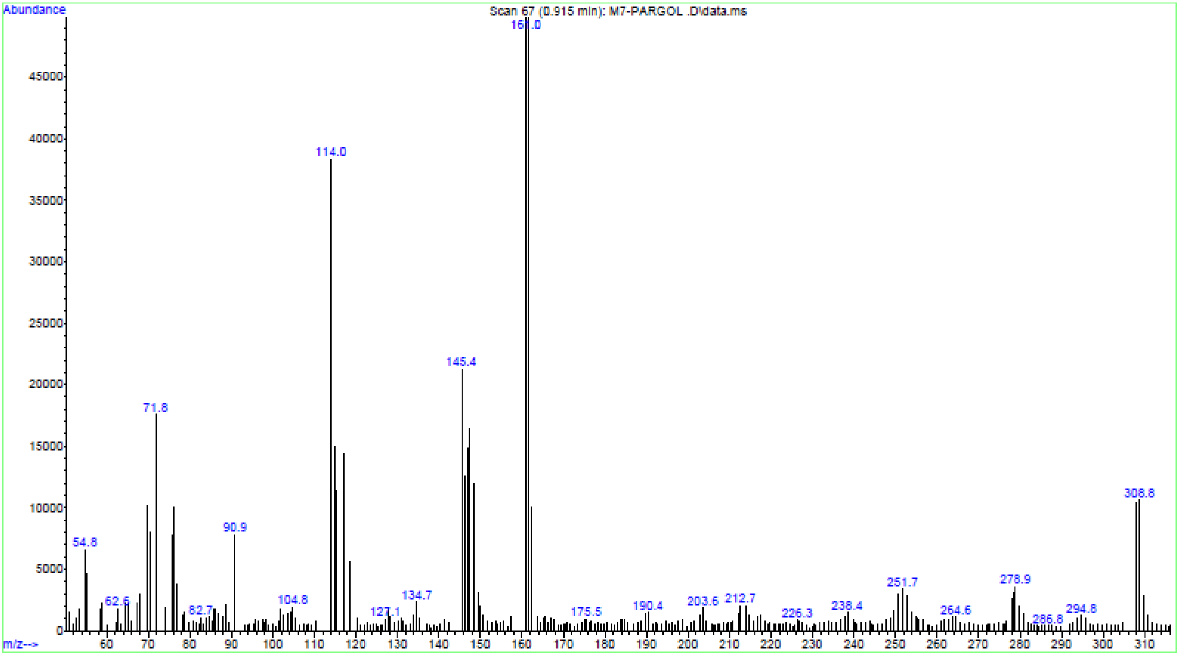

Mass spectrum of compound **6g**

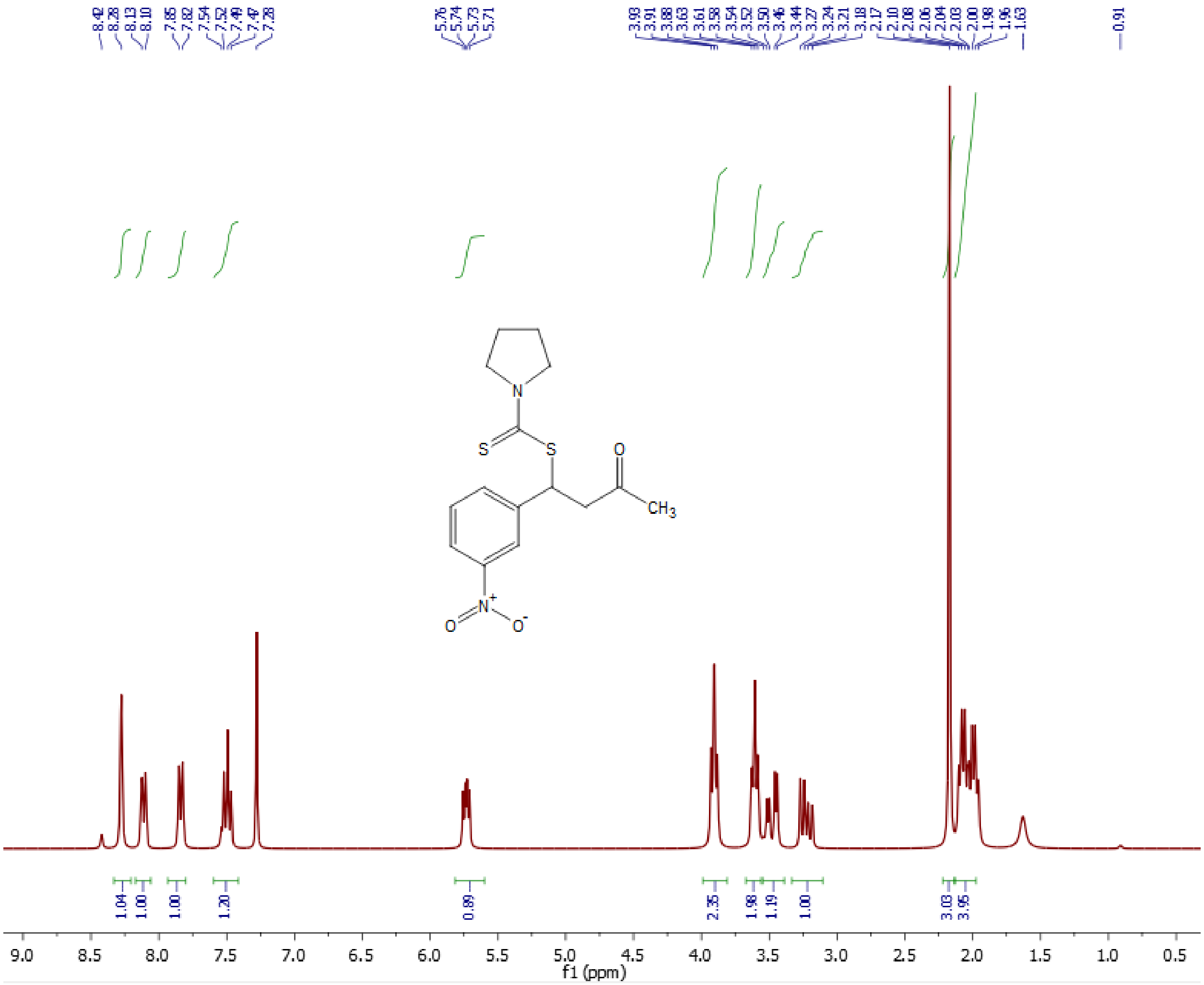

^{1}H NMR (300 MHz, chloroform-*d*) of compound **6h**

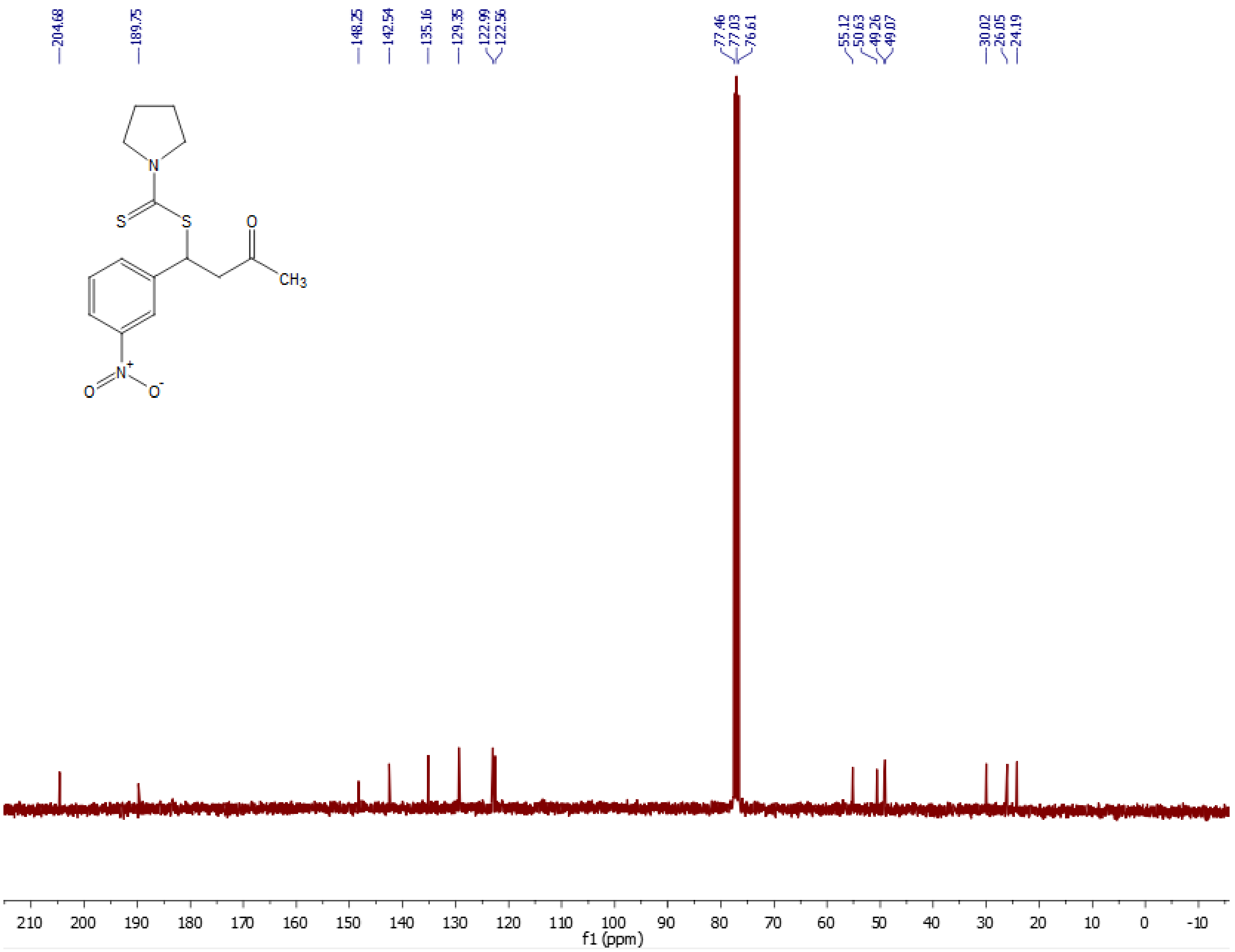

^{13}C NMR {^{1}H} (75 MHz, chloroform-*d*) of compound **6h**

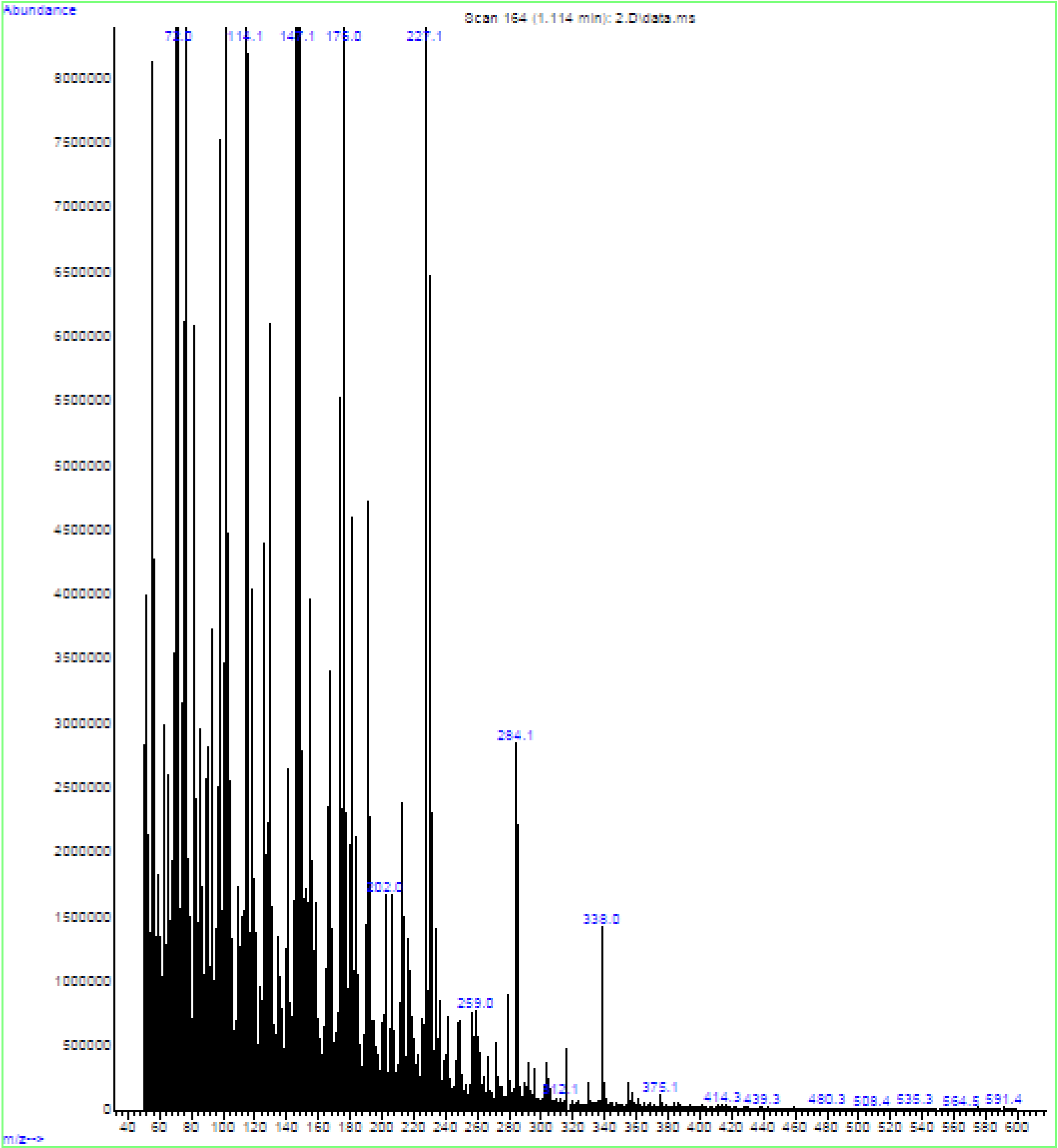

Mass spectrum of compound **6h**

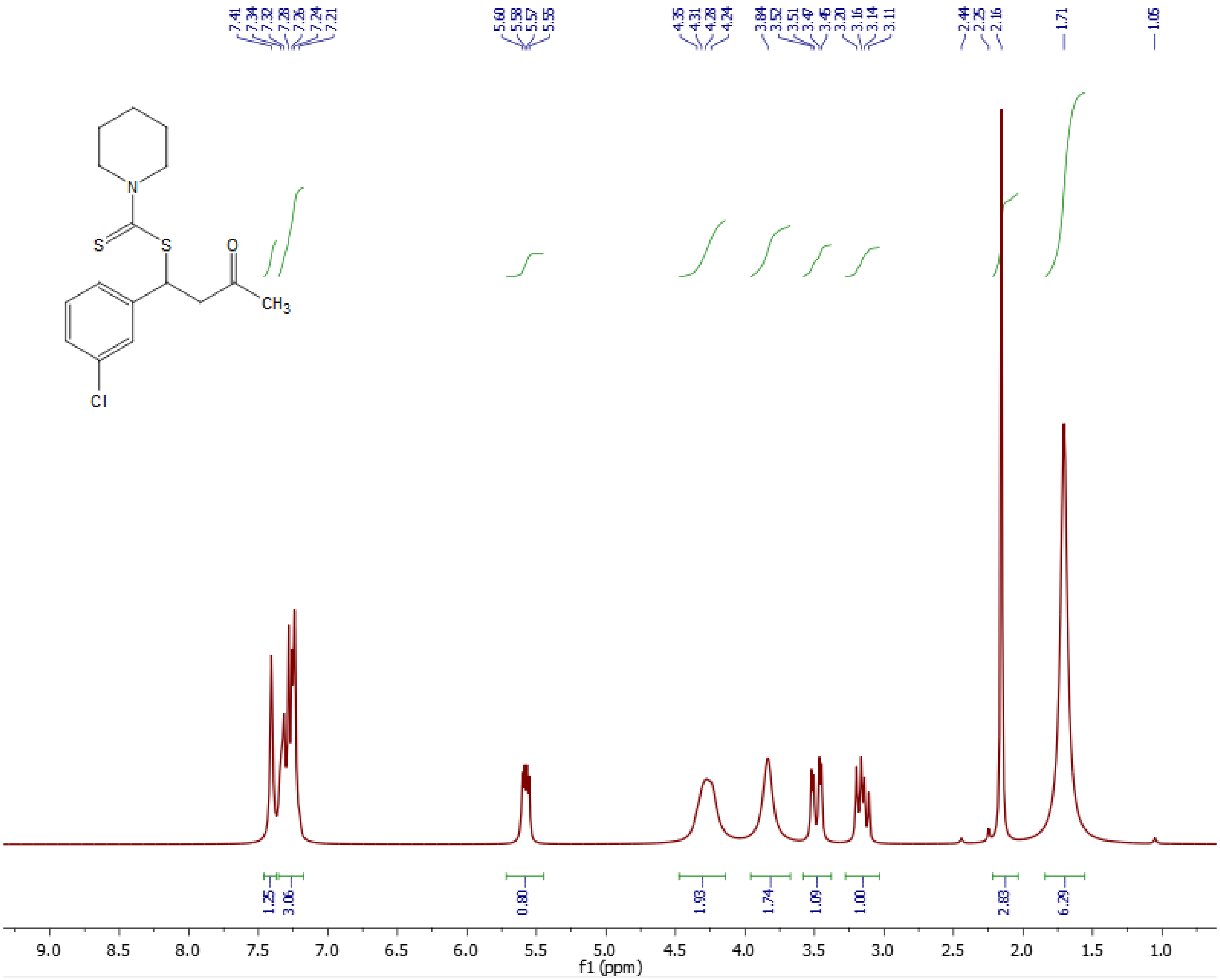

^{1}H NMR (300 MHz, chloroform-*d*) of compound **6i**

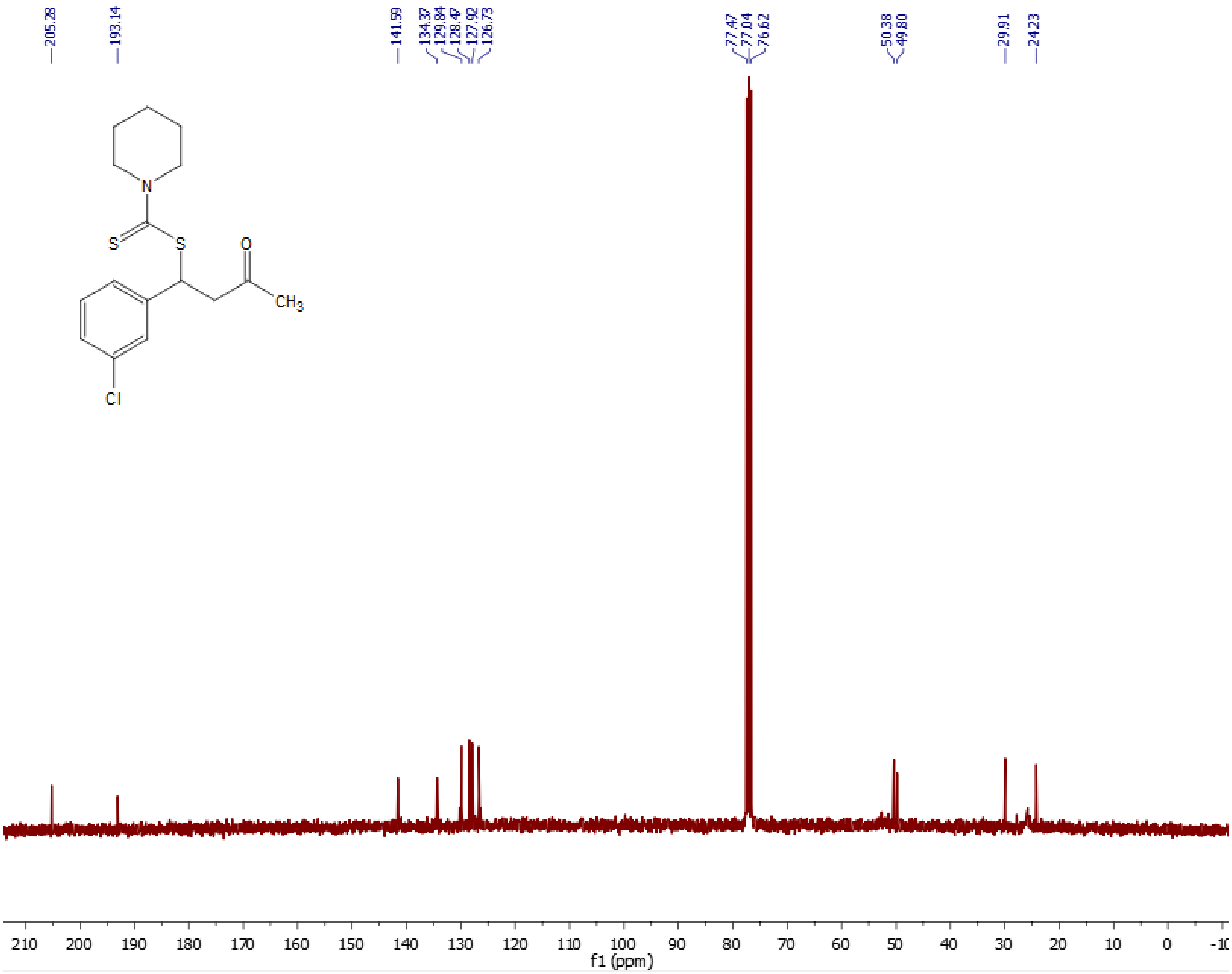

^{13}C NMR {^{1}H} (75 MHz, chloroform-*d*) of compound **6i**

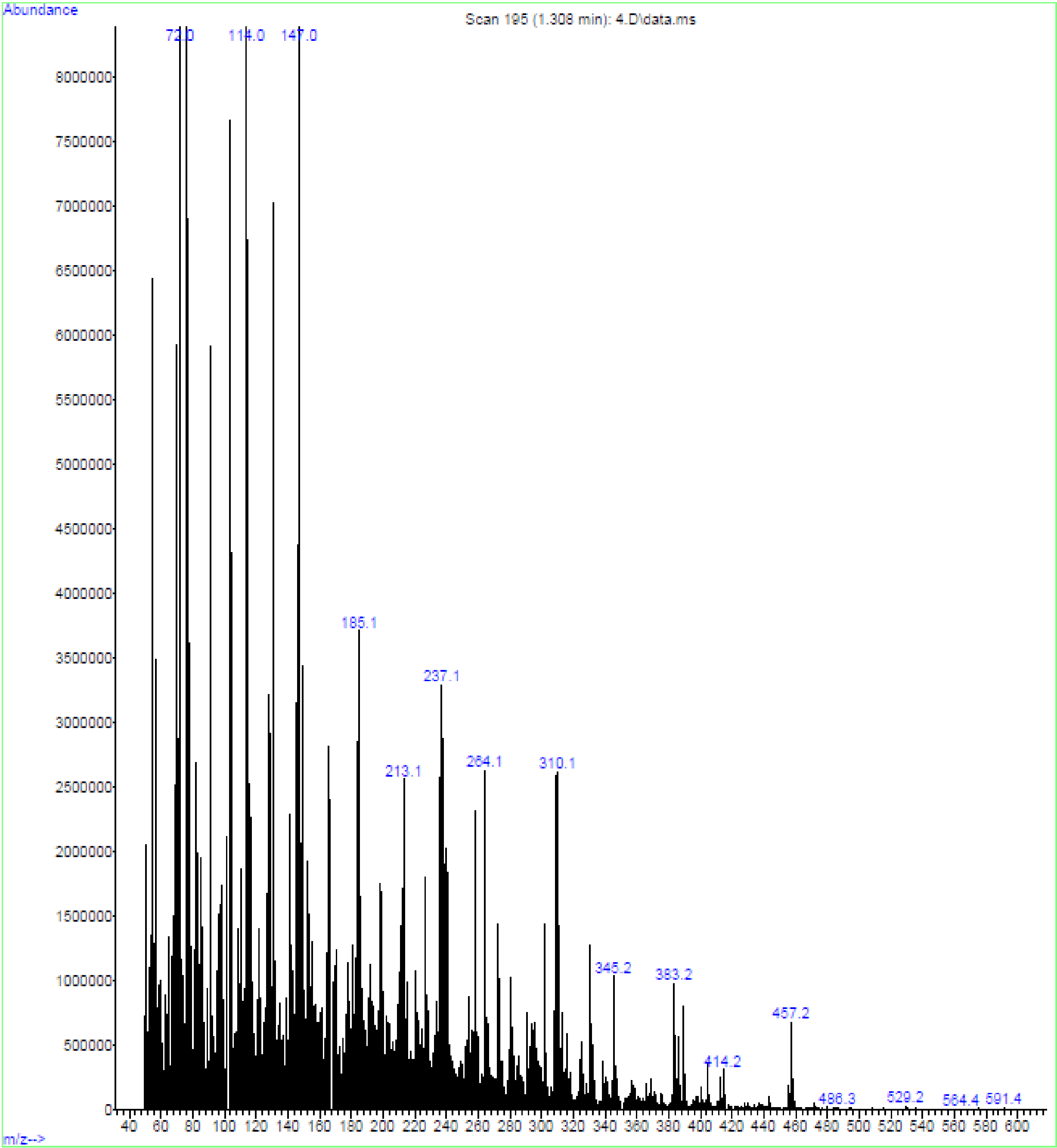

Mass spectrum of compound **6i**

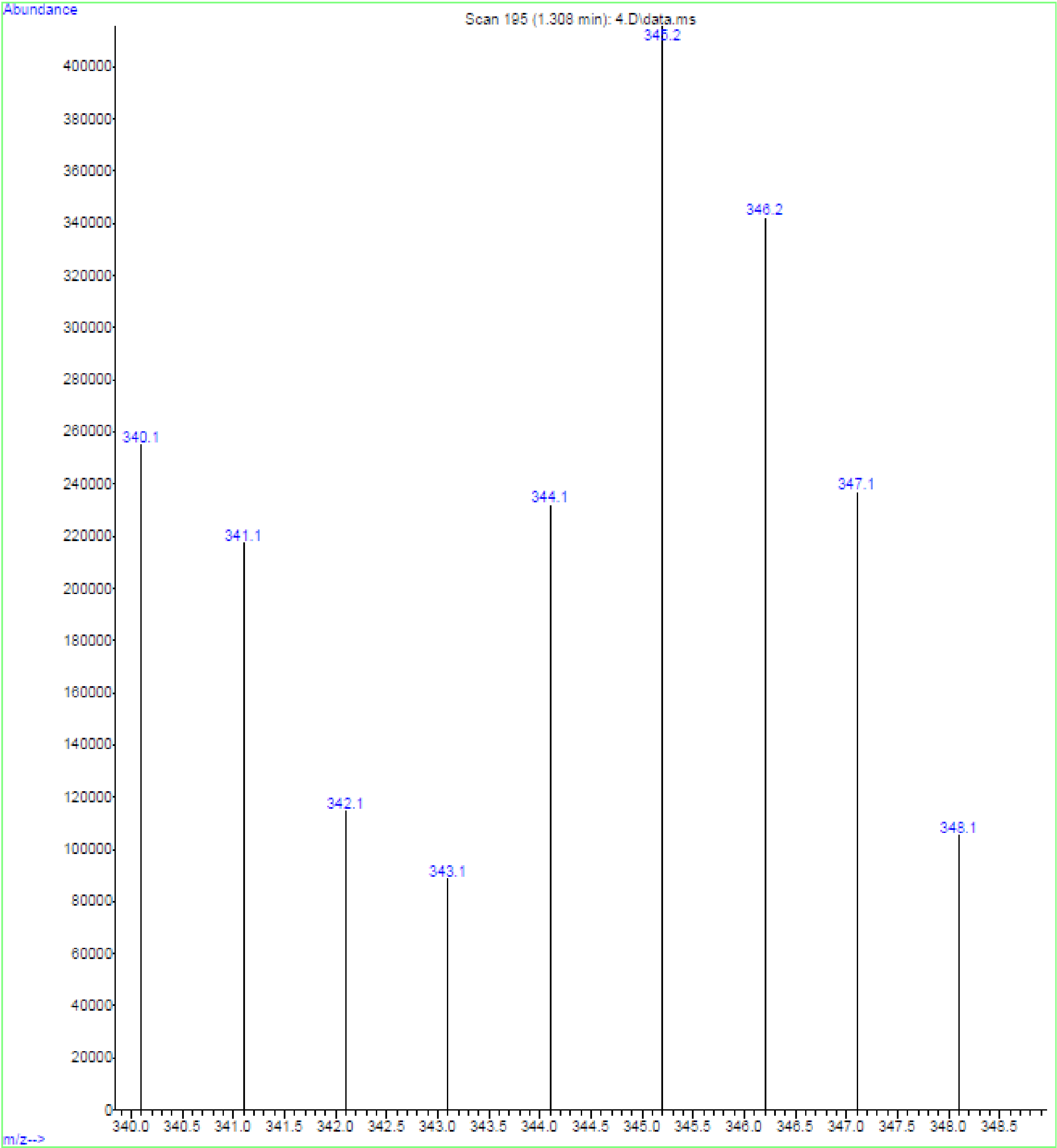

Mass spectrum of compound **6i**

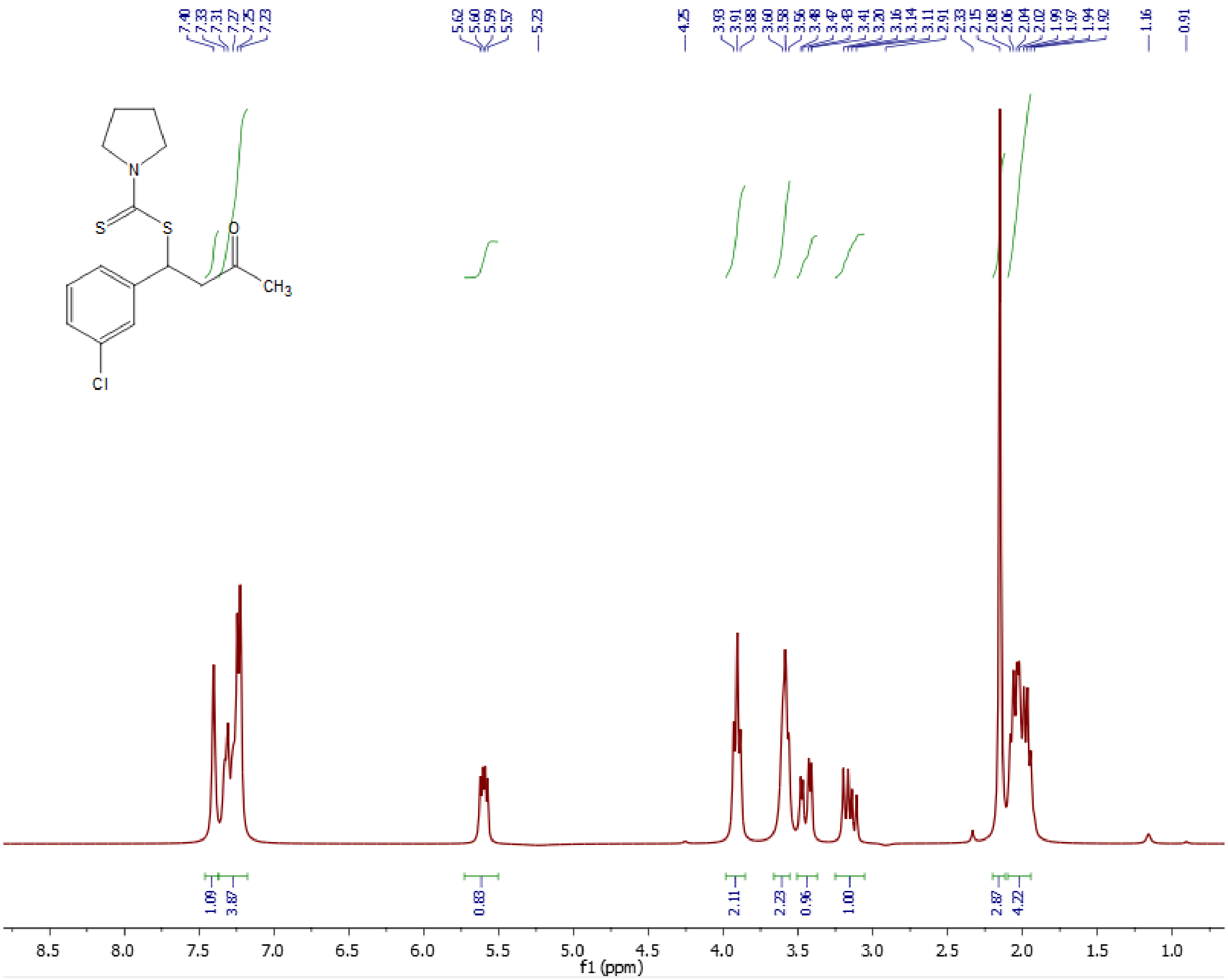

^{1}H NMR (300 MHz, chloroform-*d*) of compound **6j**

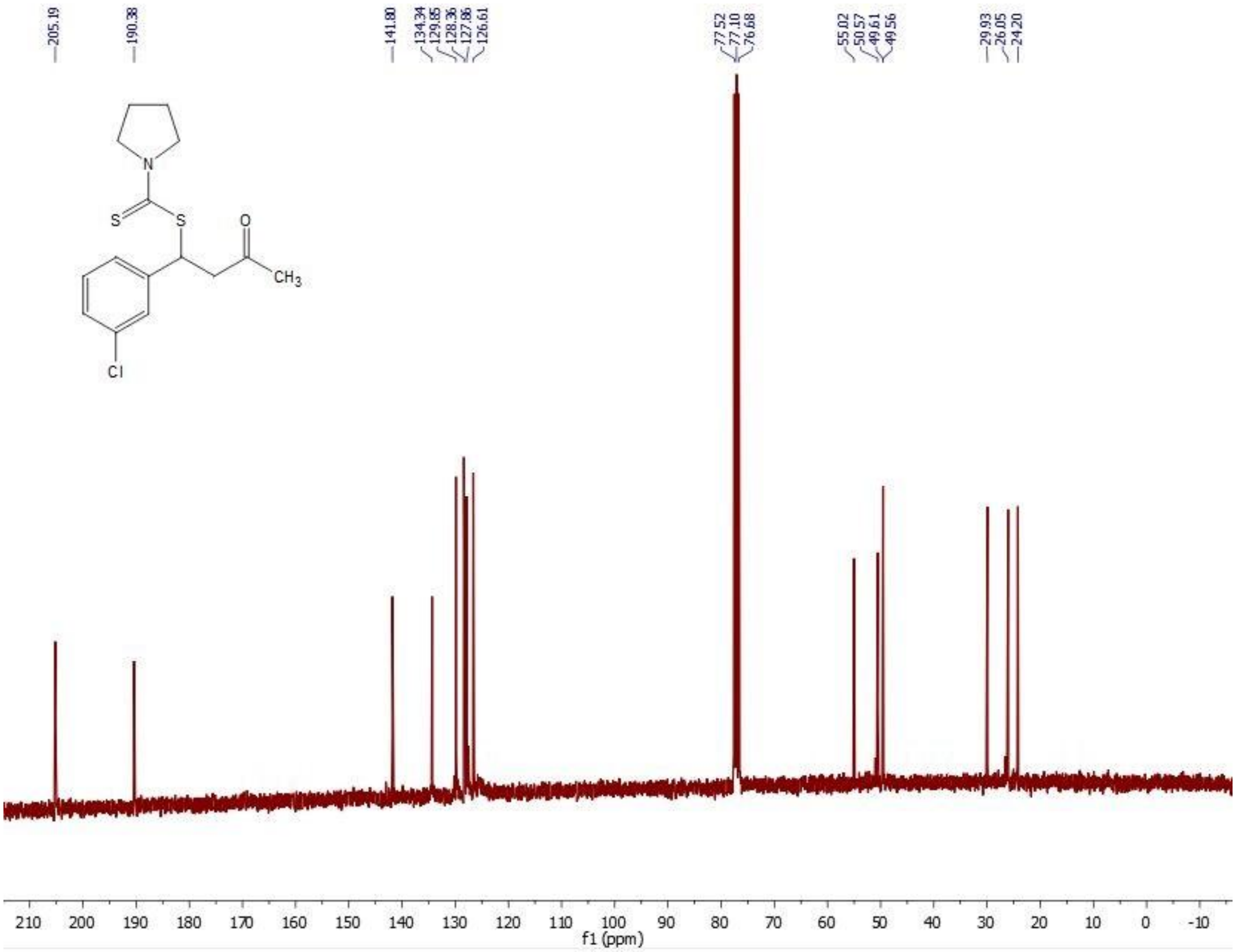

^{13}C NMR {^{1}H} (75 MHz, chloroform-*d*) of compound **6j**

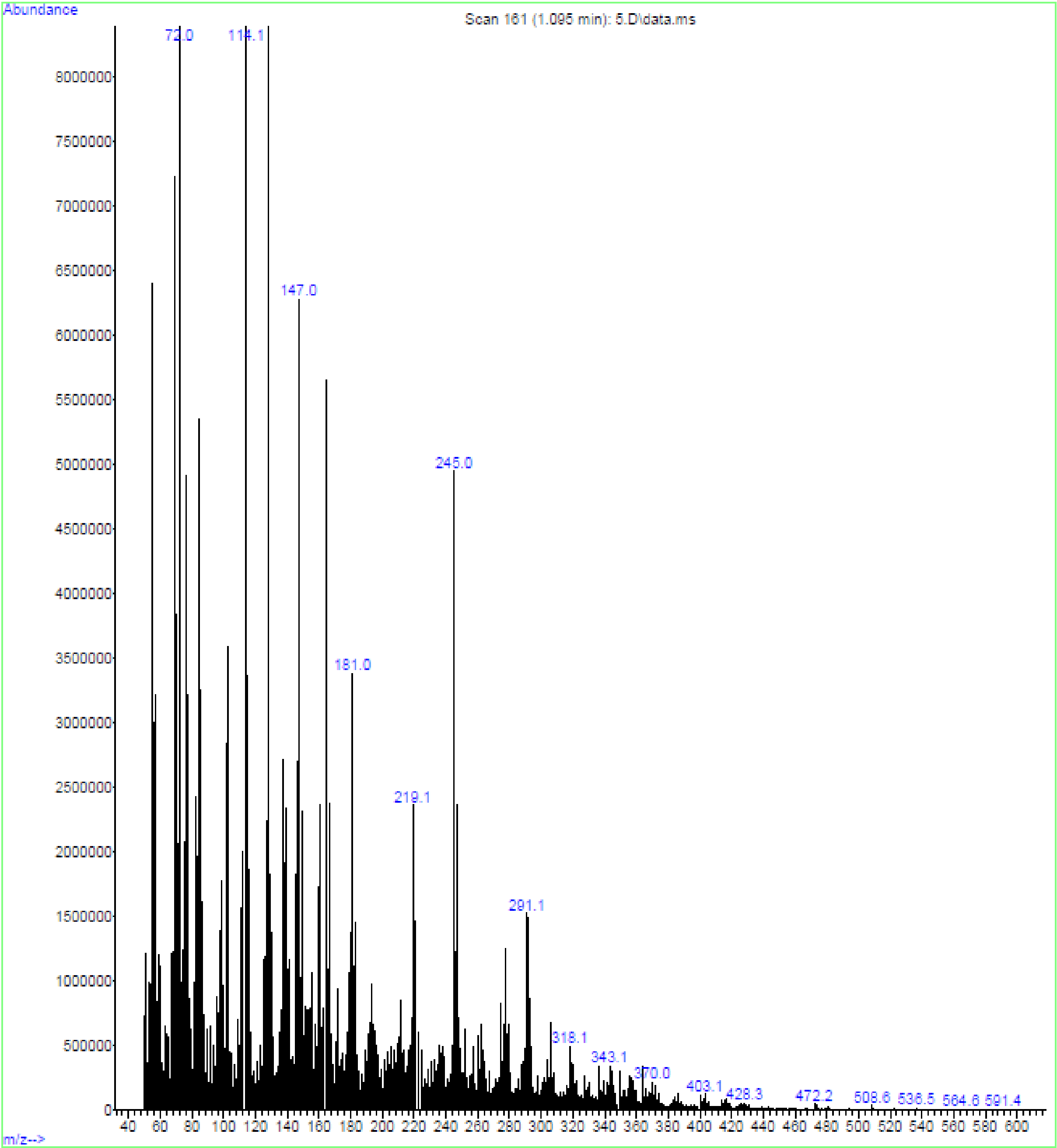

Mass spectrum of compound **6j**

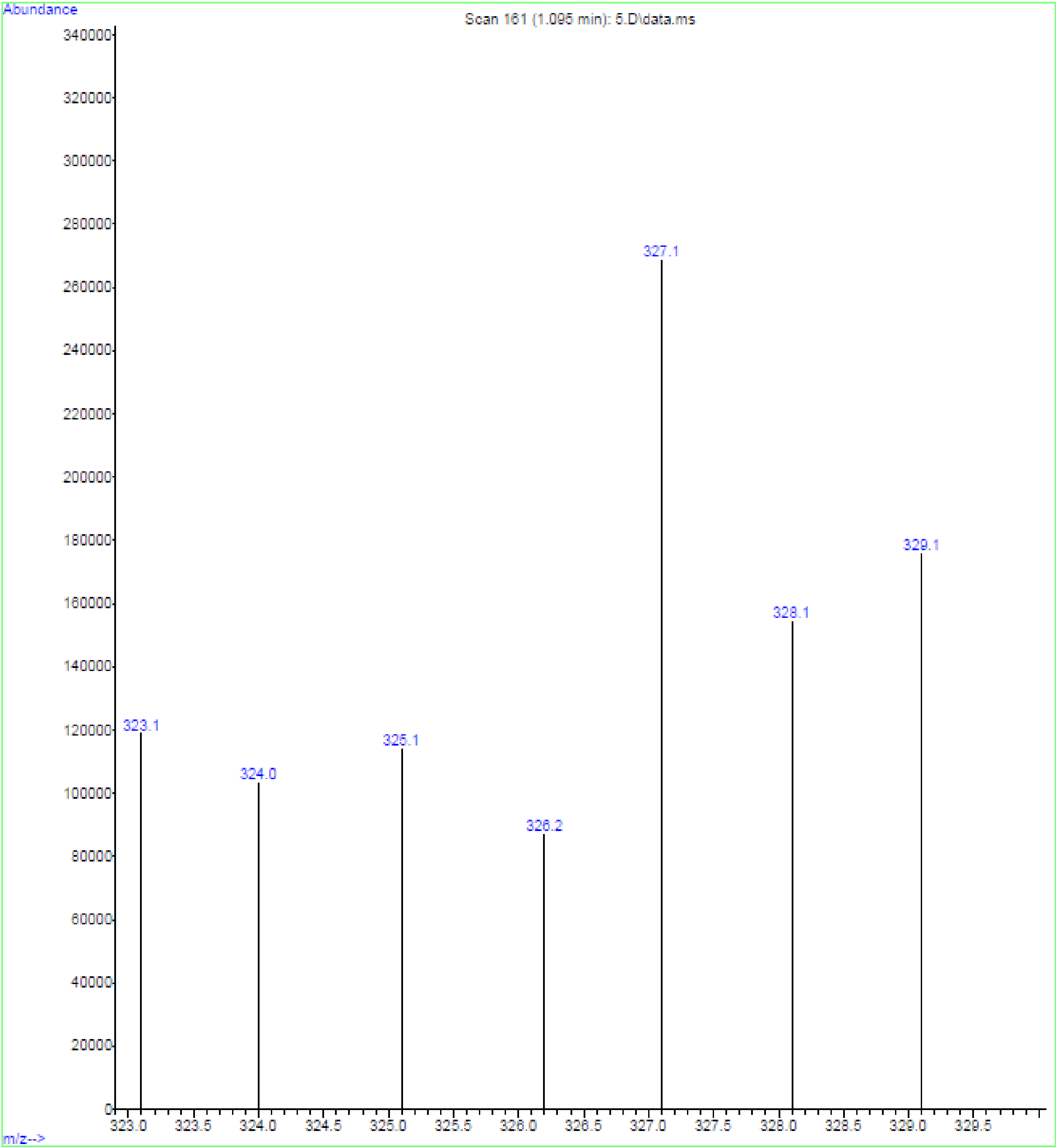

Mass spectrum of compound **6j**

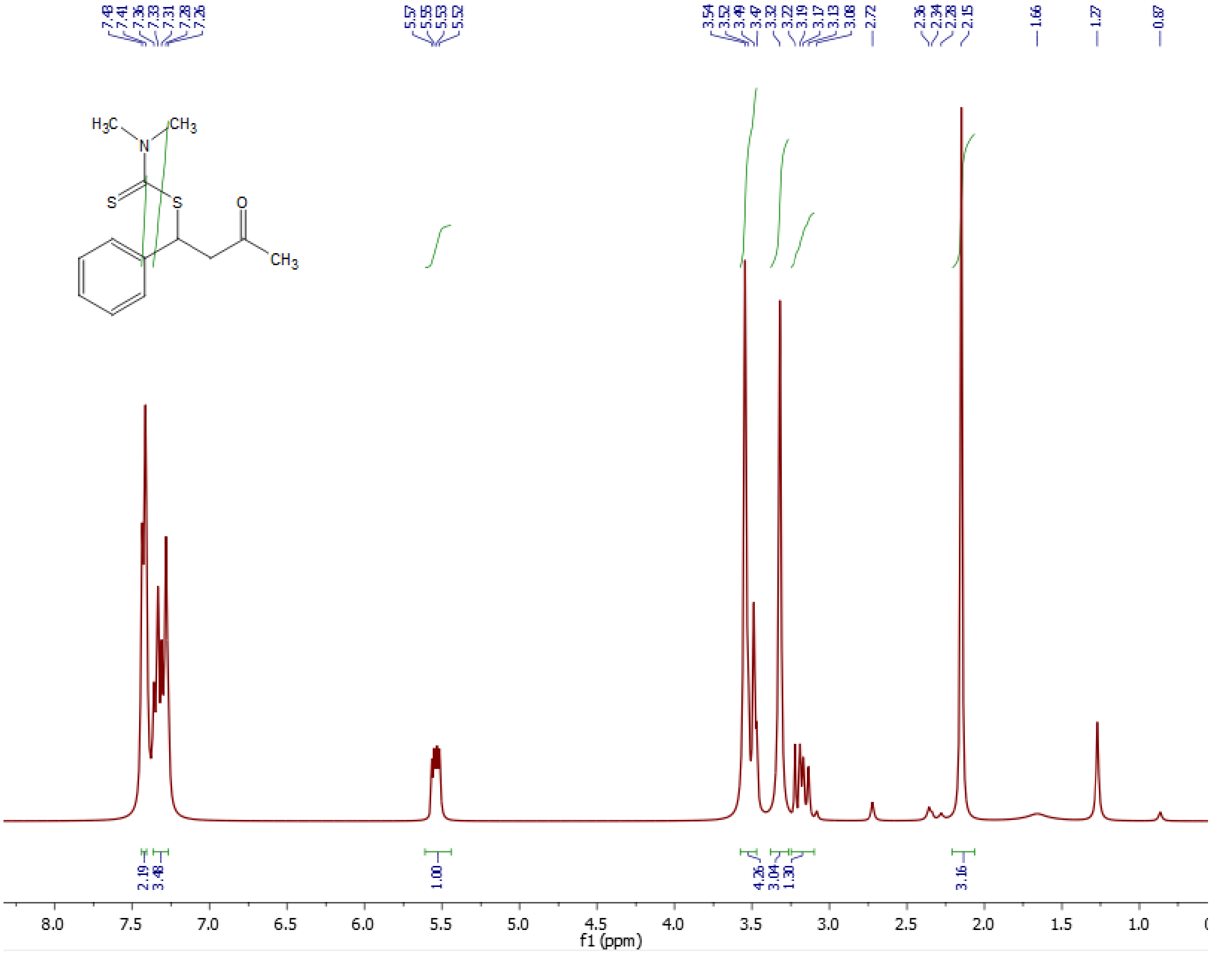

^{1}H NMR (300 MHz, chloroform-*d*) of compound **6k**

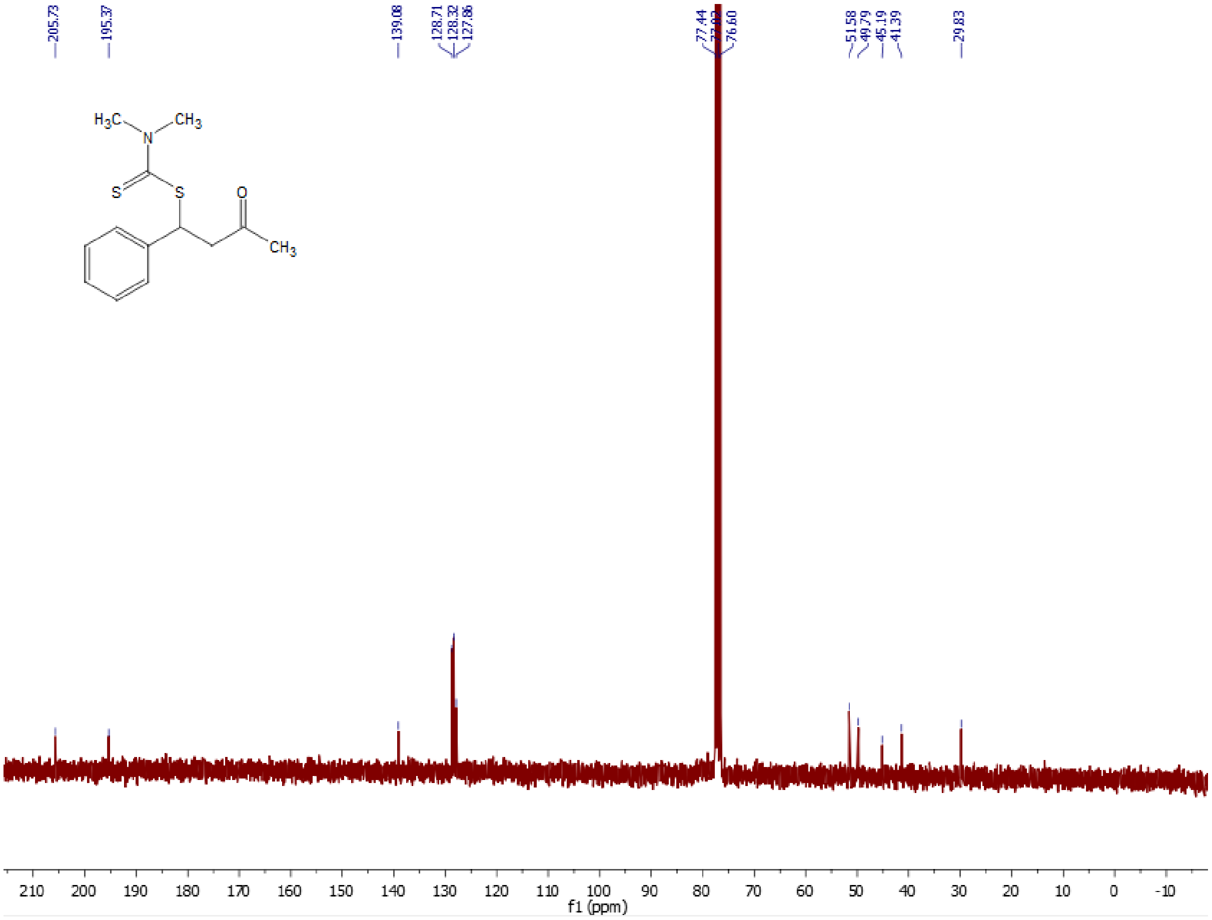

^{13}C NMR {^{1}H} (75 MHz, chloroform-*d*) of compound **6k**

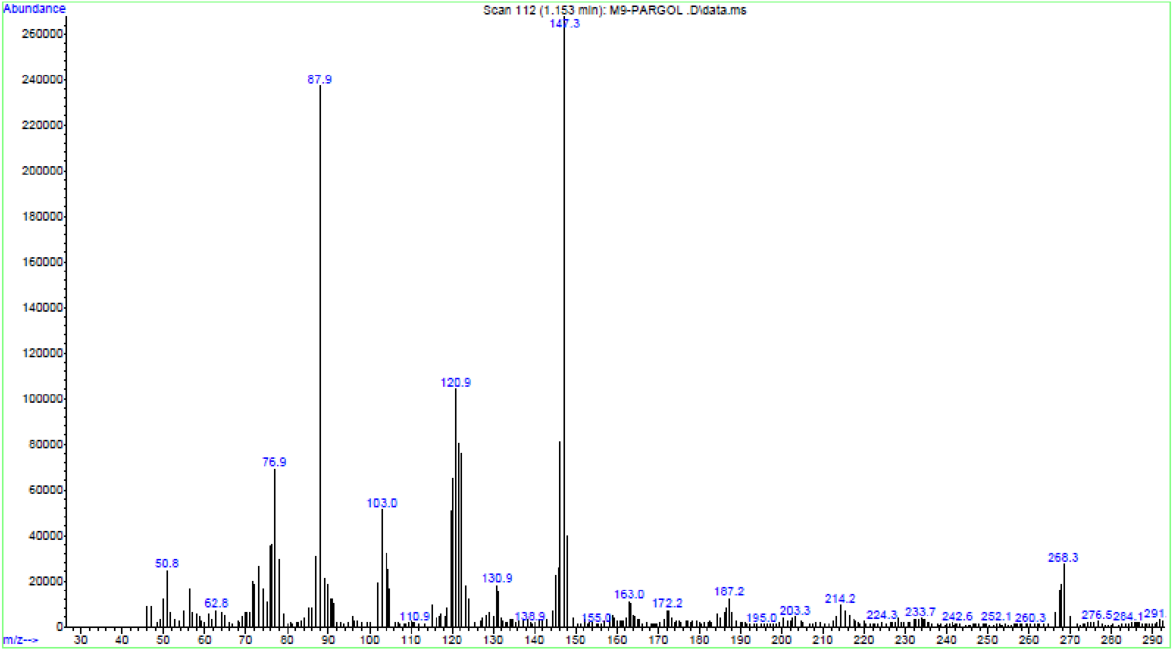

Mass spectrum of compound **6k**

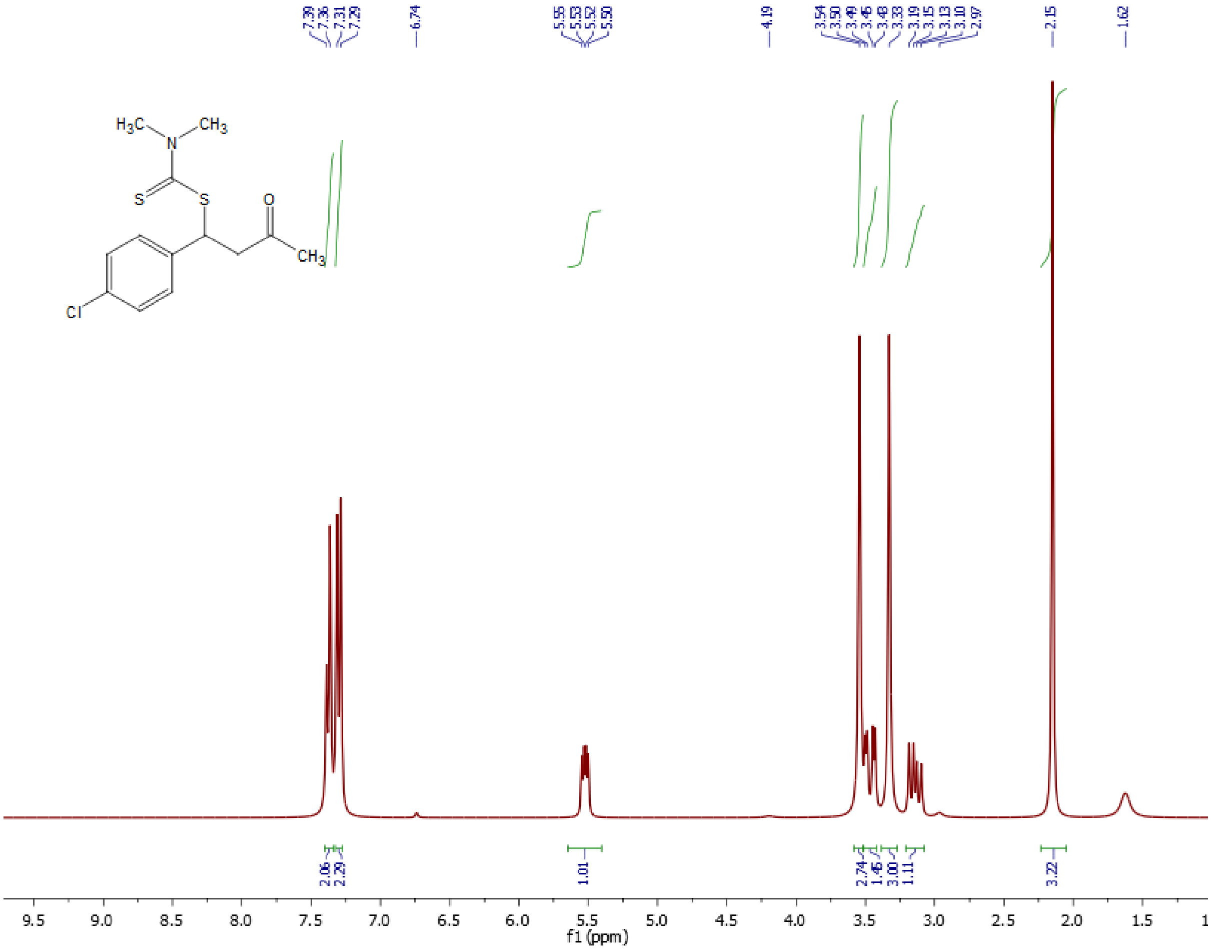

^{1}H NMR (300 MHz, chloroform-*d*) of compound **6l**

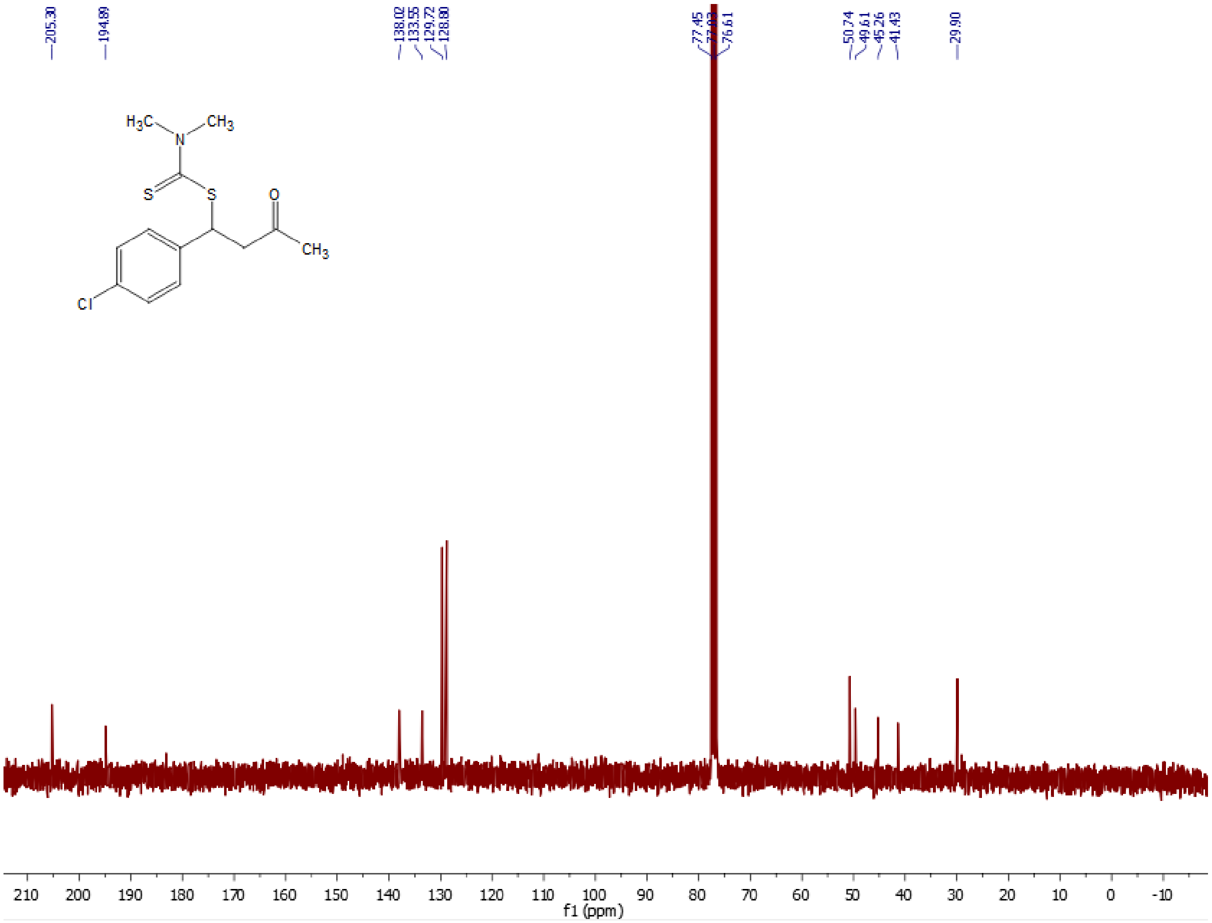

^{13}C NMR {^{1}H} (75 MHz, chloroform-*d*) of compound **6l**

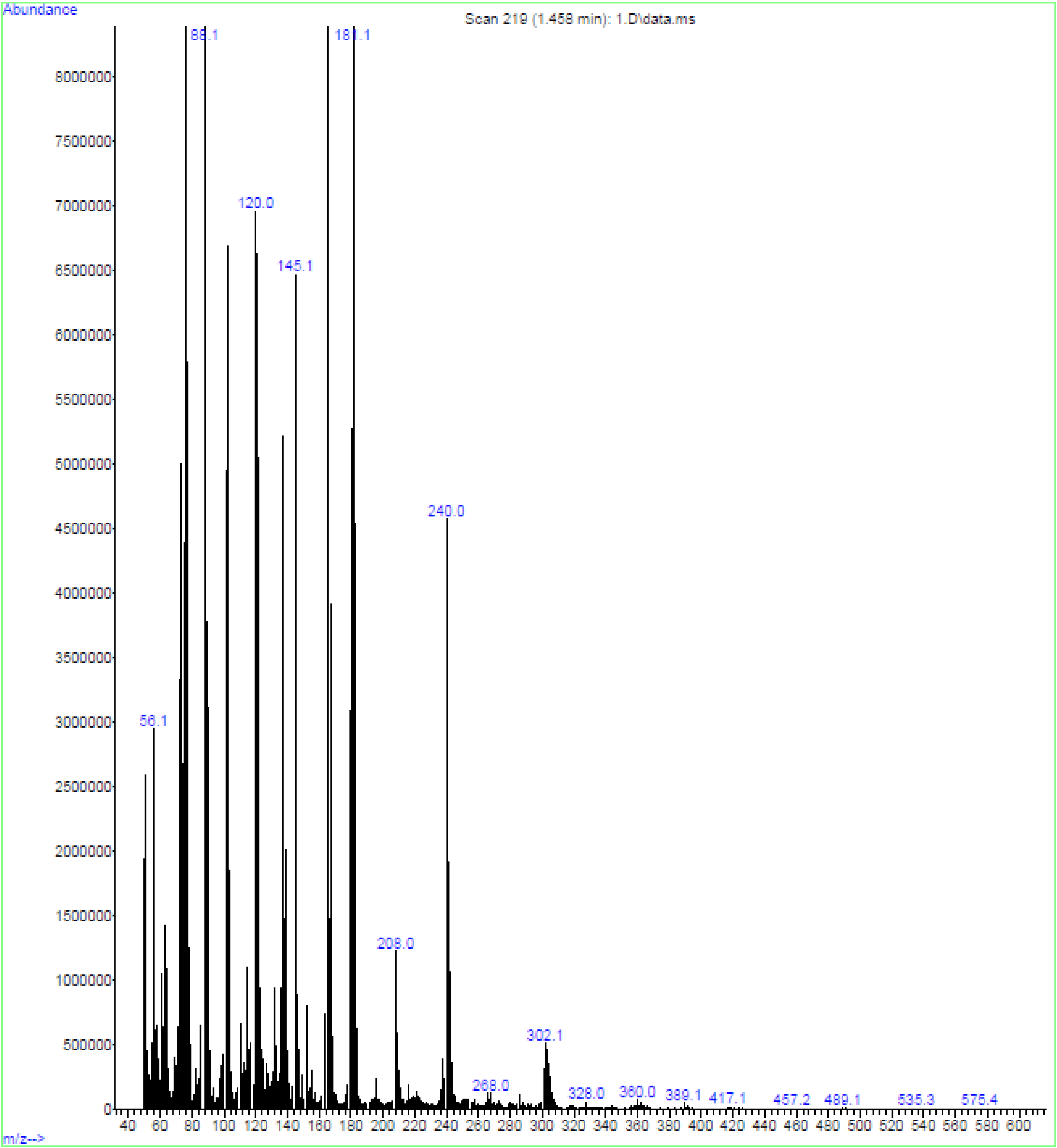

Mass spectrum of compound **6l**

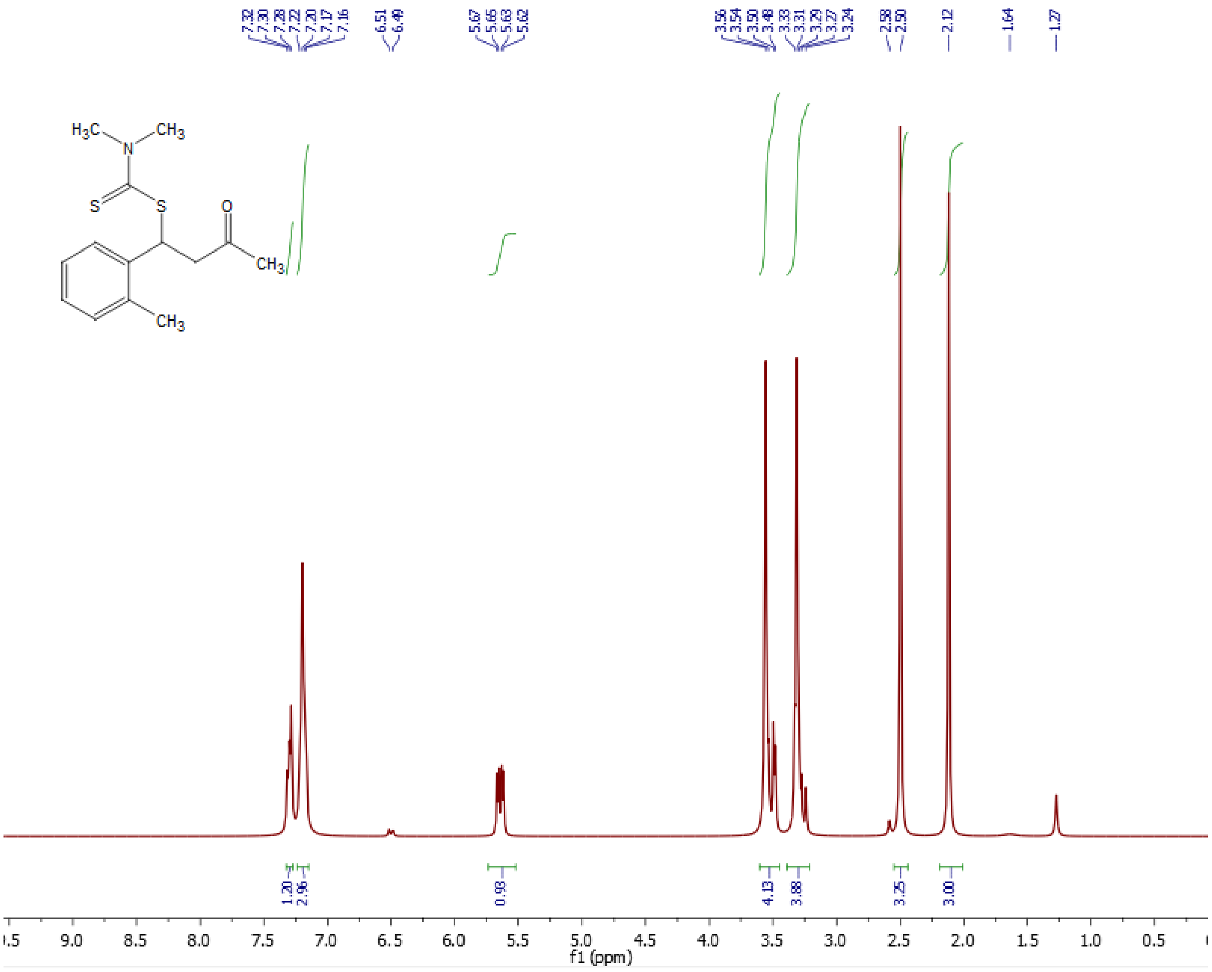

^{1}H NMR (300 MHz, chloroform-*d*) of compound **6m**

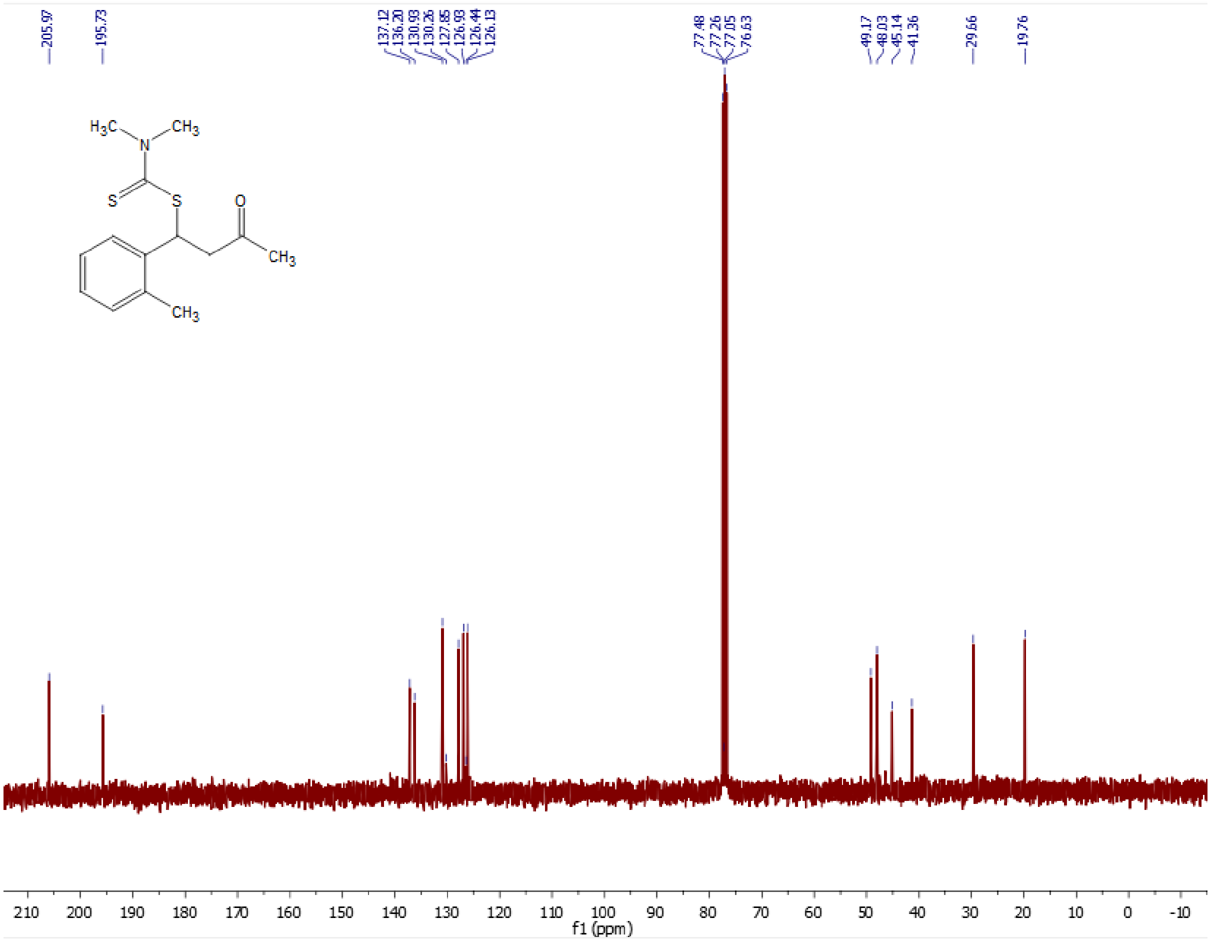

^{13}C NMR {^{1}H} (75 MHz, chloroform-*d*) of compound **6m**

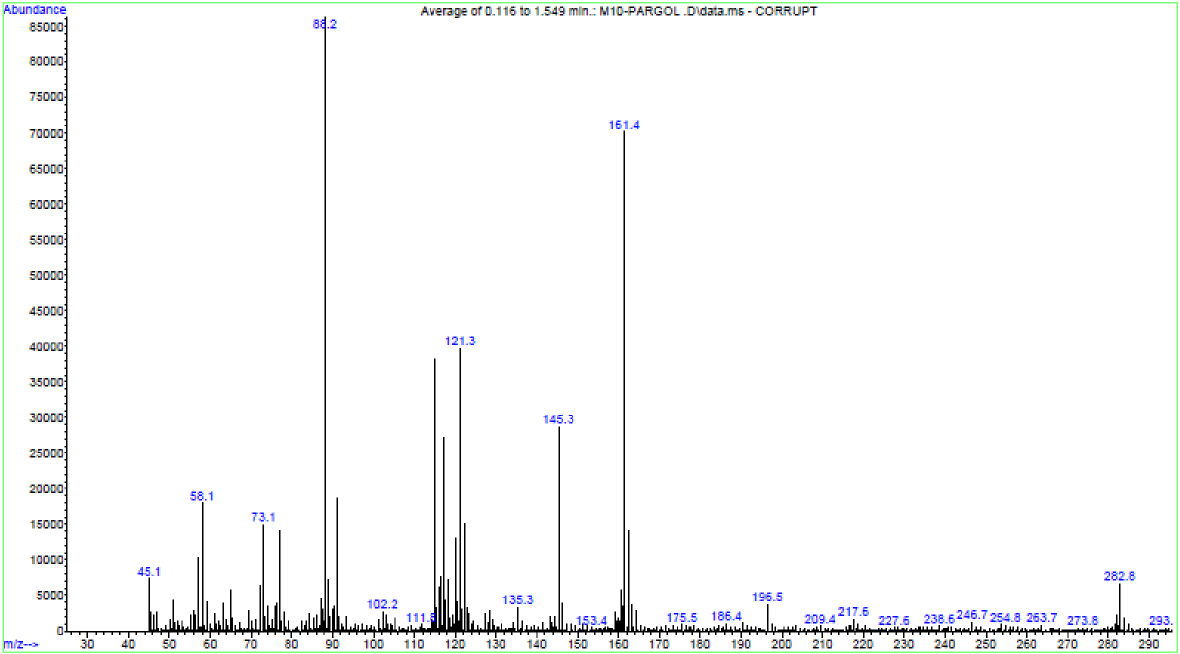

Mass spectrum of compound **6m**

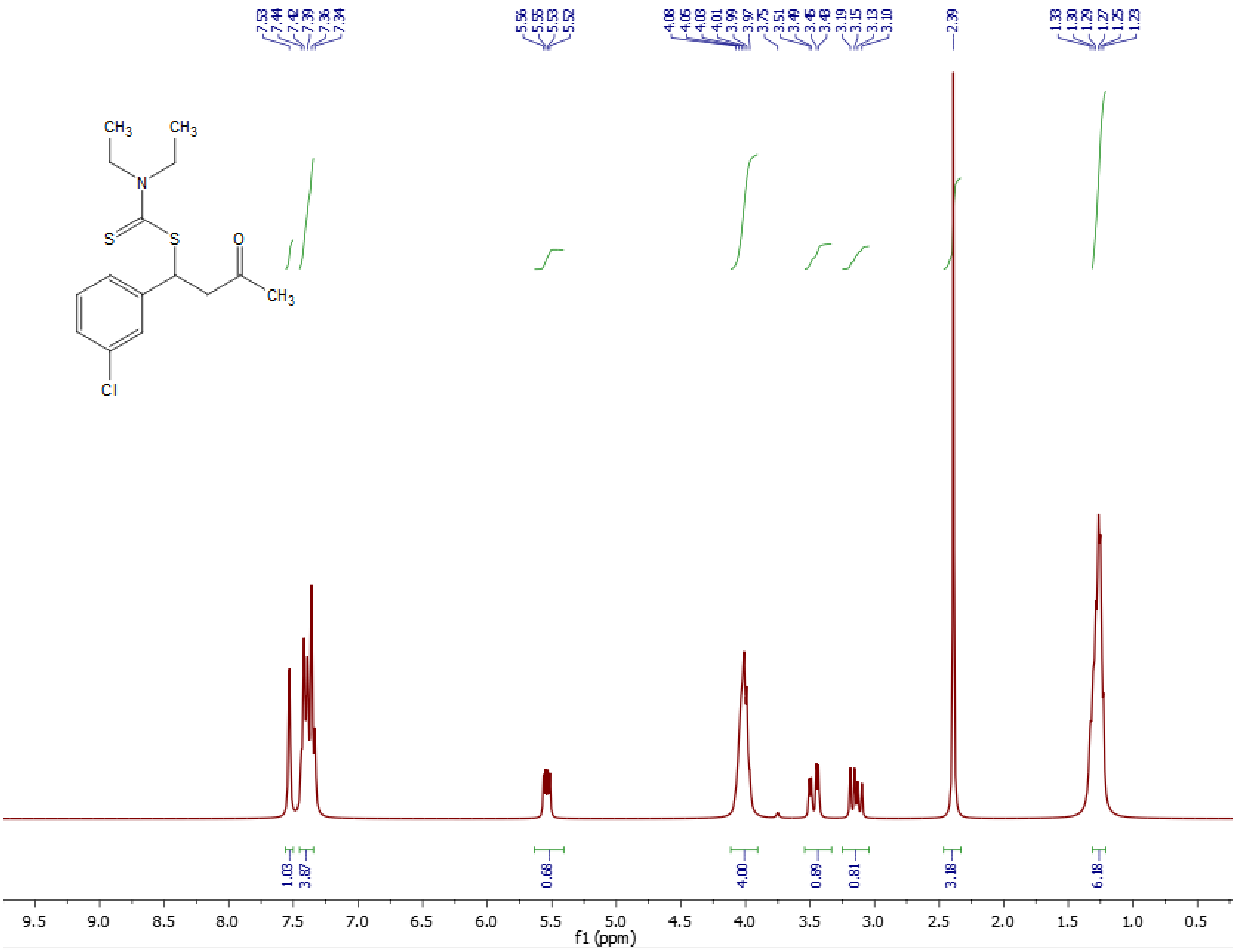

^{1}H NMR (300 MHz, chloroform-*d*) of compound **6n**

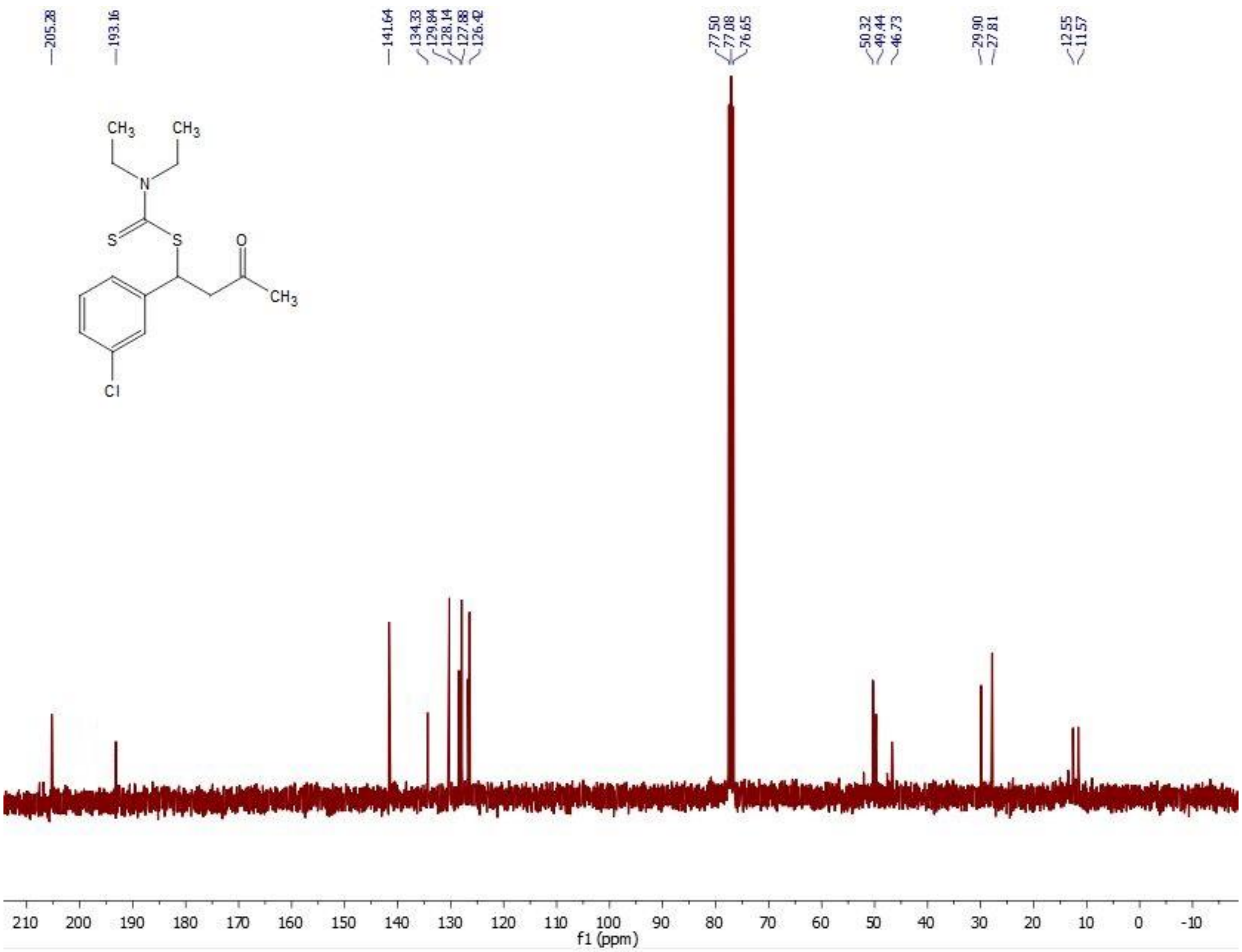

^{13}C NMR {^{1}H} (75 MHz, chloroform-*d*) of compound **6n**

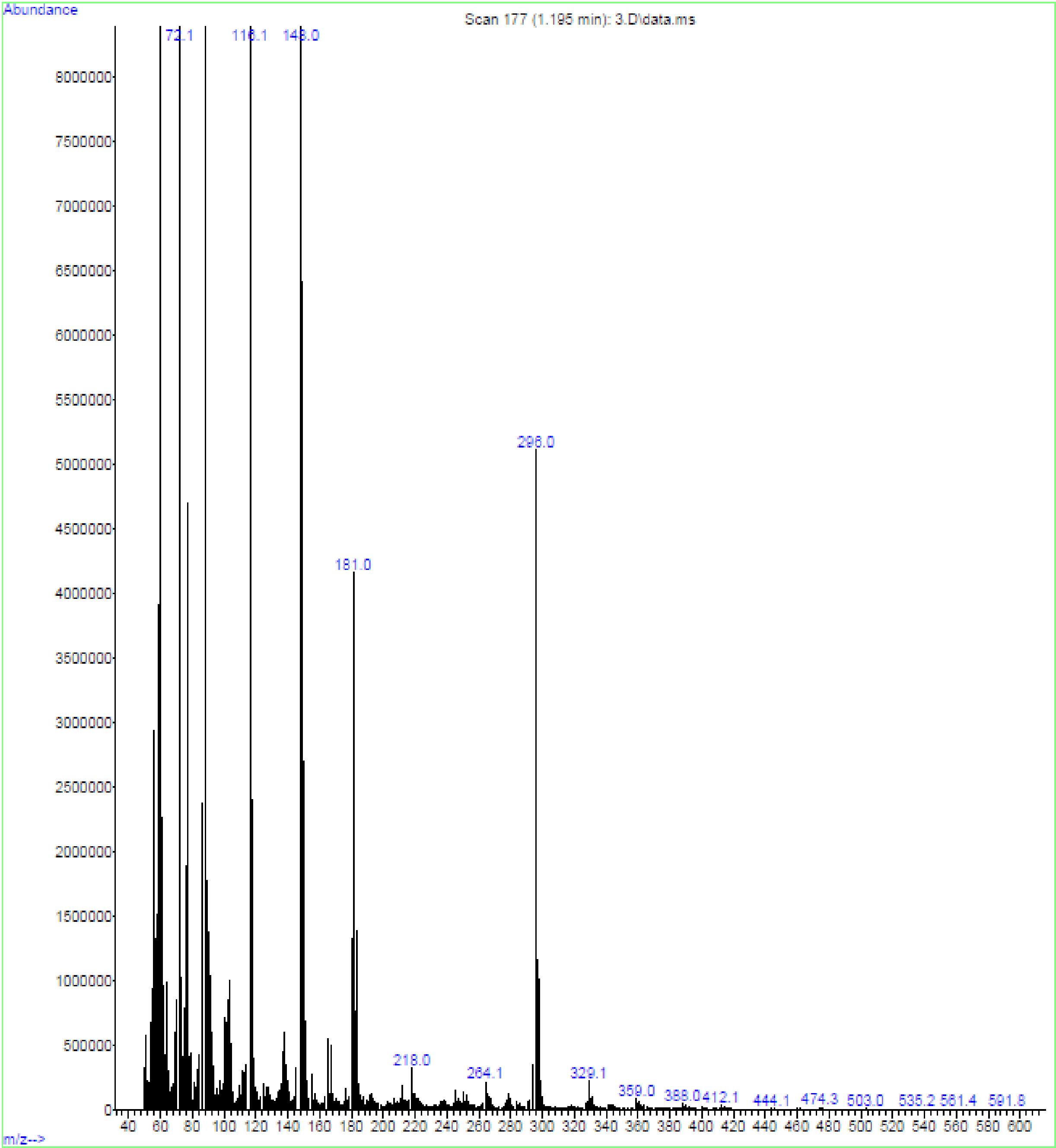

Mass spectrum of compound **6n**

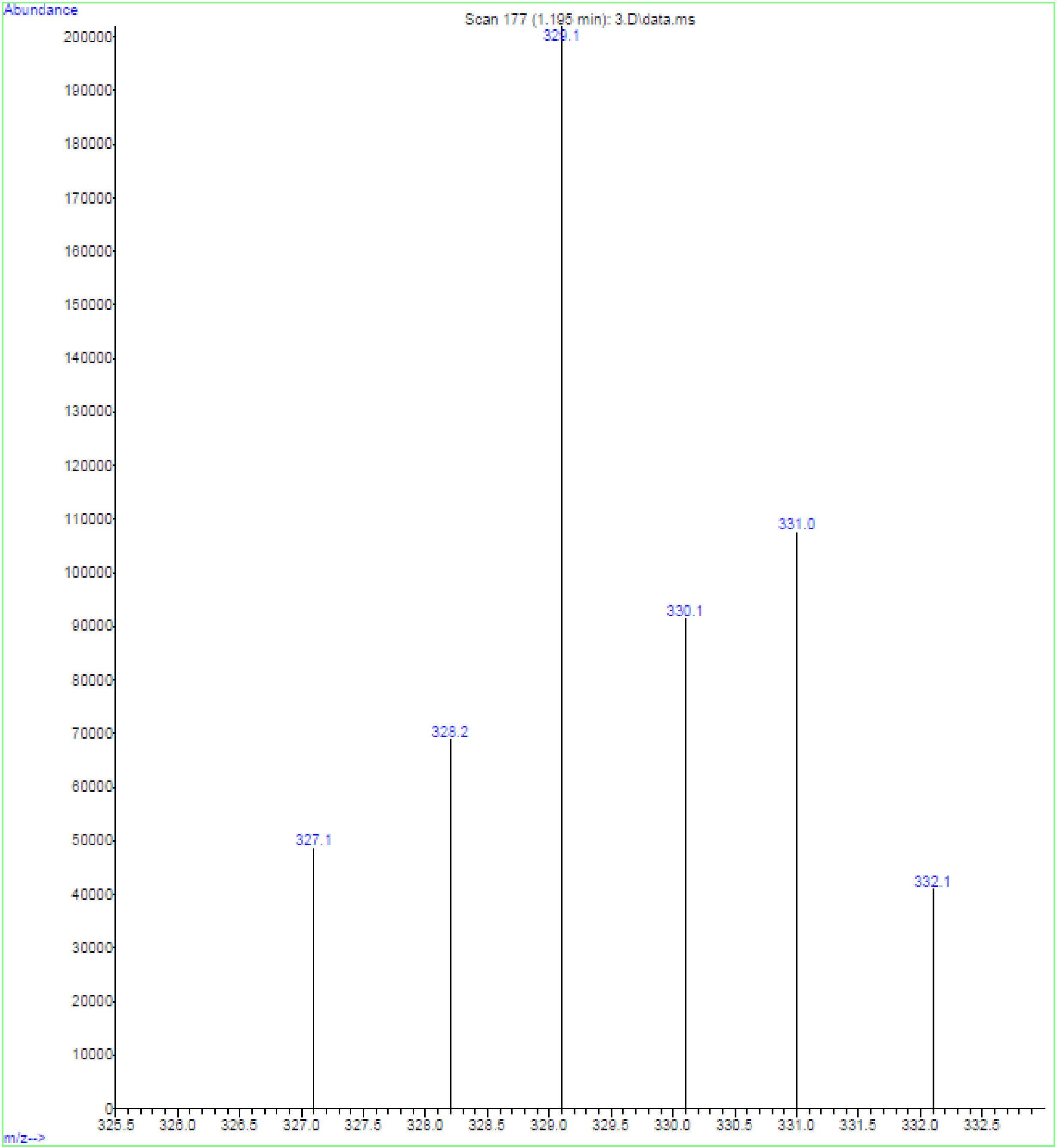

Mass spectrum of compound **6n**

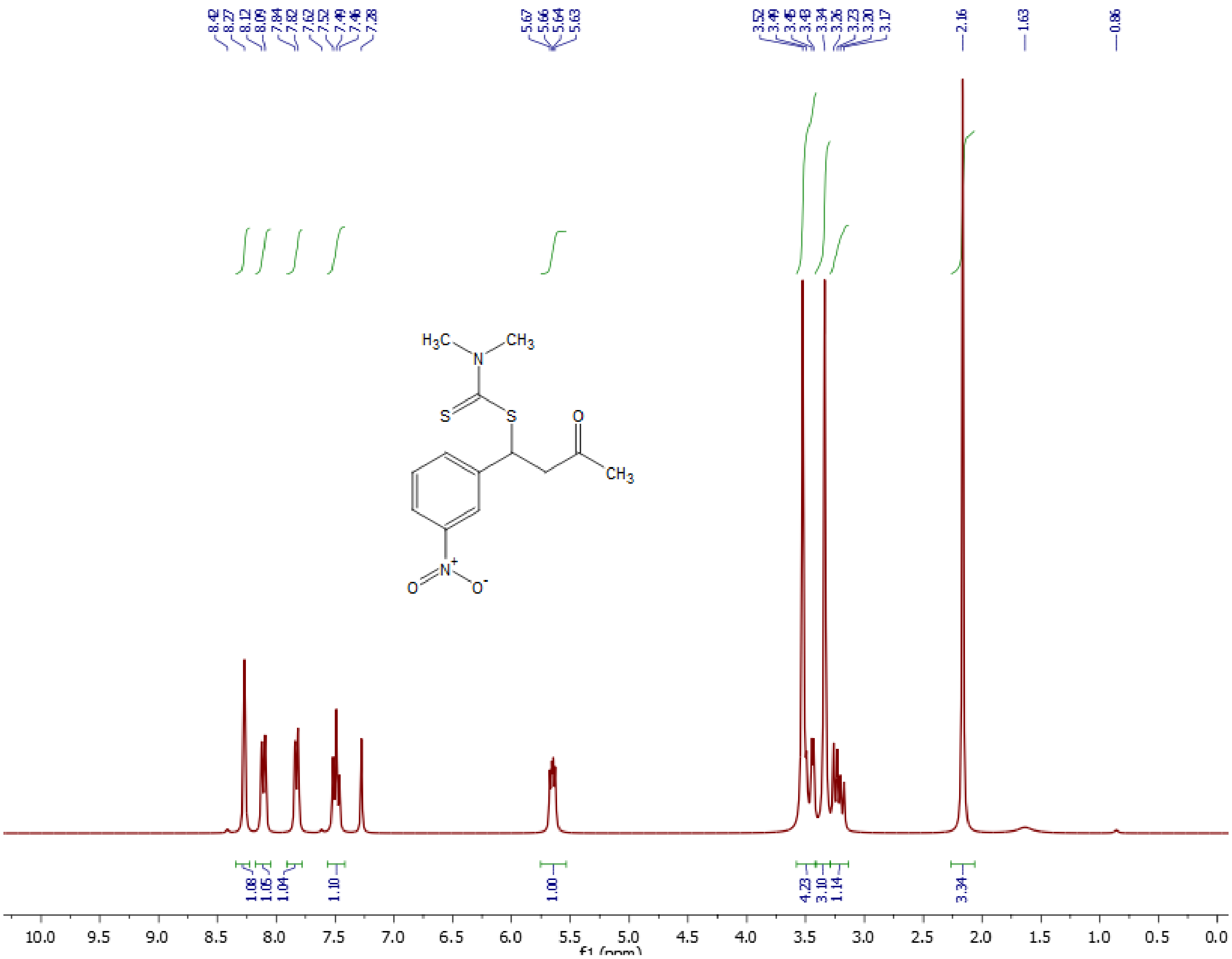

^{1}H NMR (300 MHz, chloroform-*d*) of compound **6o**

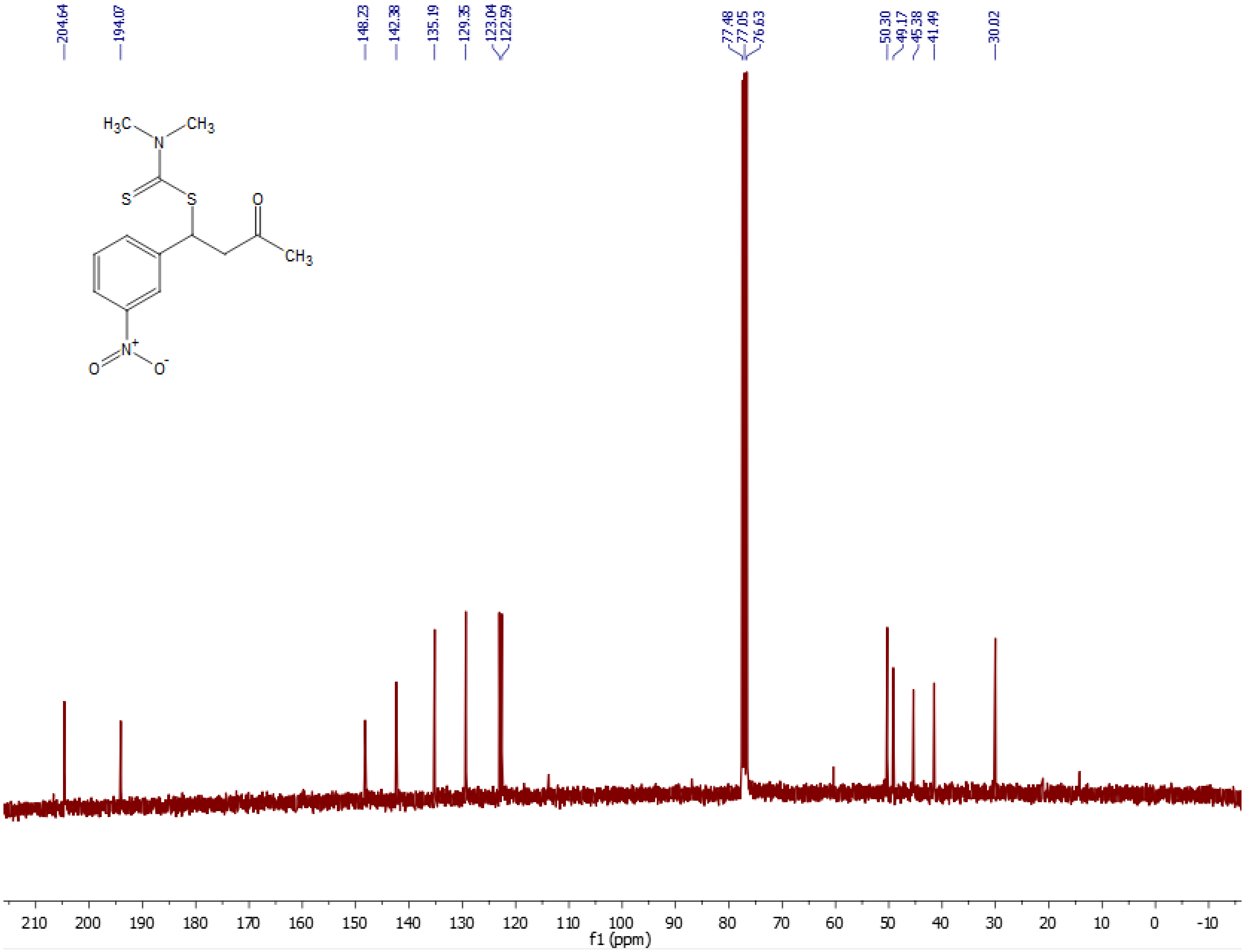

^{13}C NMR {^{1}H} (75 MHz, chloroform-*d*) of compound **6o**

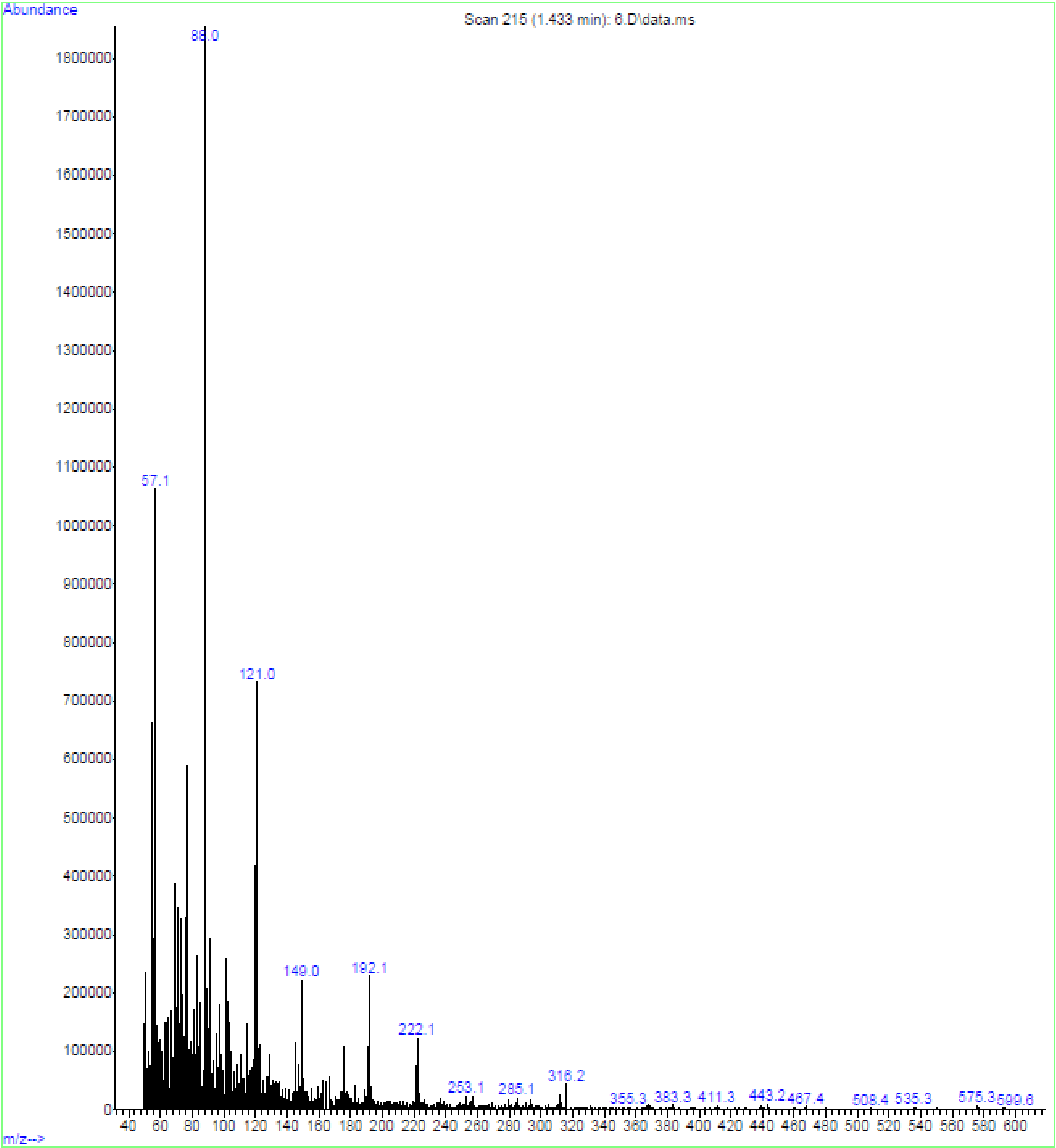

Mass spectrum of compound **6o**
